# Pd-catalyzed dehydrogenative arylation of arylhydrazines to access non-symmetric azobenzenes, including tetra-*ortho* derivatives

**DOI:** 10.3762/bjoc.21.170

**Published:** 2025-10-22

**Authors:** Loris Geminiani, Kathrin Junge, Matthias Beller, Jean-François Soulé

**Affiliations:** 1 Univ Rennes, CNRS, ISCR-UMR 6226, F-35000 Rennes, France; 2 Leibniz Institut für Katalyse, Albert Einstein Straße 18059 Rostock, Germany; 3 Chimie ParisTech, PSL University, CNRS, Institute of Chemistry for Life and Health Sciences, 75005 Paris, France

**Keywords:** azo compounds, cross-coupling, domino catalysis, palladium, phosphine ligands

## Abstract

Azobenzenes are photoresponsive compounds widely used in molecular switches, light-controlled materials, and sensors, but despite extensive studies on symmetric derivatives, efficient methods for synthesizing non-symmetric analogues remain scarce due to regioselectivity issues, multistep procedures, and limited applicability to tetra-*ortho*-substituted structures. Herein, we describe a direct, one-pot Pd-catalyzed dehydrogenative C–N coupling between aryl bromides and arylhydrazines to access non-symmetric azobenzenes. The use of bulky phosphine ligands and sterically tuned substrates promotes selective N-arylation at the terminal nitrogen. The protocol tolerates a wide range of functional groups and enables the synthesis of well-decorated azobenzenes, including tetra-*ortho*-substituted derivatives. Notably, the reaction proceeds under an O_2_ atmosphere and in the presence of water, highlighting its robustness.

## Introduction

**Figure 1:**
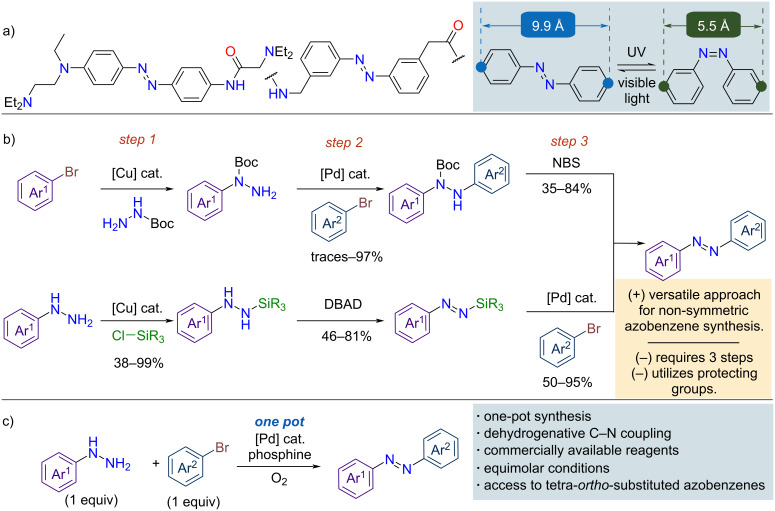
General overview of azobenzene chemistry. a) Selected examples and photoisomerization of azobenzenes; b) previous Pd-catalyzed methods for the synthesis of non-symmetric azobenzenes; c) this work: Pd-catalyzed dehydrogenative C–N coupling of arylhydrazine.

Azobenzenes are a widely studied class of compounds known for their distinctive photoresponsive properties, rendering them valuable in a variety of applications, including molecular switches, sensors, and light-controlled materials [1–8]. The photoswitching behavior arises from the reversible photoisomerization between the *E*- and *Z*-forms of the azobenzene chromophore, driven by the isomerization of the N–N double bond. This photoswitching event involves a molecular size reduction between the *E-* and *Z* isomers, thereby driving structural changes that enable applications in molecular machines, biological allosteric modulators, and advanced functional materials (Figure 1a) [9–11]. Despite the widespread interest in azobenzenes, most synthetic methods have focused on the preparation of symmetric derivatives [12–13]. Traditional approaches, such as oxidative coupling of anilines [14–19], reductive coupling of nitroarenes [20–23], or cross-coupling between anilines and nitroarenes have proven efficacious but face significant challenges when applied to non-symmetric systems [24], particularly in achieving regioselectivity. These methods frequently require a particular reagent pair or an excess of one reactant, which limits their efficiency and versatility. In contrast, Baeyer–Mills reactions, which rely on nitroso-aniline couplings, provide a route for the synthesis of non-symmetric azobenzenes, but their substrate specificity and use of hazardous precursors limit their practical applicability [25–28]. An alternative approach involves the S_E_Ar reaction, which utilizes potentially hazardous diazonium salts and electron-rich arenes (mainly limited to phenols) [29–31], including metalated arenes [32–33].

The growing demand for structurally complex compounds across diverse applications has rendered the synthesis of non-symmetrical azoarenes with differently substituted azo bonds intricate and inefficient using these standard synthetic protocols. The transition-metal-catalyzed C–N bond formation has emerged as a viable route to access non-symmetric azobenzenes, owing to the broad functional group tolerance of Buchwald–Hartwig amination reactions [34–40]. Kong and co-workers developed a Chan–Evans–Lam-type oxidative cross-coupling reaction between *N*-arylphthalic hydrazides and arylboronic acids using copper catalysis [41]. Similarly, in 2003, Lee and co-workers introduced a desymmetrization approach employing simpler N=N precursors, specifically N-protected hydrazines. Their method involved a three-step process comprising Cu and Pd-catalyzed C–N bond formations followed by a dehydrogenative deprotection step (Figure 1b, top) [42]. This desymmetric approach was further employed by Oestreich and co-workers in 2022, who introduced silicon-masked diazenyl anions in a Pd-catalyzed three-step sequence to access a wide range of non-symmetric azobenzenes (Figure 1b, bottom) [43–44].

Inspired by these approaches and building on recent advances in the dehydrogenation of 1,2-diarylhydrazines to azobenzenes [45–50], we developed a one-pot strategy for synthesizing non-symmetric azobenzenes via a Pd-catalyzed cascade involving C–N coupling of arylhydrazines with aryl bromides, followed by oxidative dehydrogenation (Figure 1c). As our study was nearing completion, Váňa and co-workers disclosed a related Buchwald–Hartwig approach employing the Pd(OAc)₂/BINAP catalytic system [51], which enabled selective C–N coupling at the terminal nitrogen and suppressed denitrogenative by-products [52–54]. Although the general strategy is similar, our method uses the [PdCl(C_3_H_5_)]_2_/*t-*BuXPhos catalytic system, which provided higher yields and broader substrate scope (functional group tolerances), including challenging tetra-*ortho*-substituted azobenzenes.

## Results and Discussion

We started our investigation by using phenylhydrazine (**1a**) and 2-bromotoluene (**2a**) as model substrates. In the beginning, standard Buchwald amination conditions were tested ([PdCl(allyl)]_2_, XPhos, *t-*BuONa in 1,2-dimethoxyethane (DME)) [55]. The reaction outcomes were analyzed with both GC/MS and GC/FID analysis. Under these conditions, we were pleased to find that 1-phenyl-2-(*o*-tolyl)diazene (**3a**) was obtained with a GC yield of 50% (Table 1, entry 1). While all starting materials were consumed, various impurities, including biphenyls, diarylamines, aniline, and toluidine, were formed in varying amounts. Since a final dehydrogenation step is required to complete the reaction, the experiment was repeated with various oxidants tested as additives. The use of di-*tert*-butyl peroxide as an oxidant, combined with NaH as the base, increased the formation of **3a**, achieving GC yields of up to 65% (Table 1, entry 3). Control experiments demonstrated that palladium, phosphine, and base were all essential for this reaction (Table S4 in Supporting Information File 1). However, under identical conditions with other aryl bromides like 4-bromotoluene, the reaction failed to form azobenzene **3b**, instead yielding 1-phenyl-1-(*p*-tolyl)hydrazine, resulting from arylation of the central nitrogen (Table 1, entry 4 and Supporting Information File 1). This unexpected result prompted us to further optimize the reaction conditions, with particular focus on the choice of ligand, especially for non-*ortho*-substituted aryl bromides as substrates. Notably, the use of bulkier phosphines, such as P(*t*-Bu)_3_ and *t*-BuXPhos, was found to promote the reaction regardless of the substitution pattern of the bromotoluene (Table 1, entries 5–8). For subsequent optimizations, we selected *t*-BuXPhos for practical reasons, as it is less sensitive to oxidative conditions. Further optimization revealed that Cs_2_CO_3_ is more efficient than NaH for this reaction (Table 1, entry 9). However, reactions conducted with a new batch of Cs_2_CO_3_ showed a dramatic reduction in product yield (Table 1, entry 12). Control experiments with varying amounts of water (0–10 equiv) demonstrated that a small amount of water is crucial for the reaction (Supporting Information File 1, Table S7 and Table 1, entry 11). This effect, previously reported in Buchwald–Hartwig reactions, enhances yield by facilitating the reduction of Pd(II) to Pd(0) and improving the solubility of the base [56]. To ensure the generality of the conditions, 1-bromo-2-(trifluoromethoxy)benzene was also tested, yielding the desired azobenzene **3c** in 69% yield with the addition of 2 equivalents of water (Table 1, entries 12 and 13). Finally, the oxidant *t-*Bu-OO-*t-*Bu could be replaced by O_2_, yielding compound **3a** with GC yields of up to 79% (Table 1, entry 14, 75% isolated yield). In reactions giving <70% isolated yield, no single side-product dominates; the missing mass is apportioned among several minor by-products (and small handling losses), with no evidence for a favored competing pathway. Notably, this work led to the development of a reaction protocol that operates without the need for inert conditions and tolerates small amounts of water, simplifying practical implementation. We finally evaluated 1.5 equivalents of either aryl bromide or phenylhydrazine and observed no gain in yield (differences within experimental error) (Table 1, entries 15 and 16); accordingly, we retained a 1:1 stoichiometry, which maximizes atom economy, lowers PMI/E-factor by avoiding excess reagent, and simplifies purification and scale-up.

**Table 1:** Selected optimization of conditions for the synthesis of azobenzene from phenylhydrazine and aryl bromides. The gray box provides a detailed list of all identified side-products.

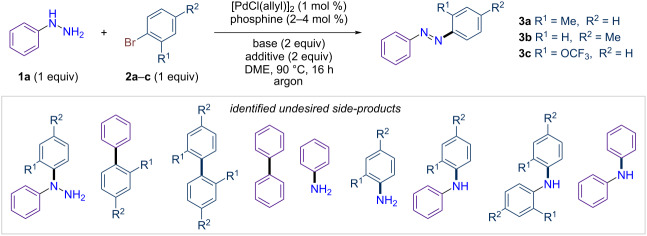						

Entry	**2a, 2b** or **2c**	Phosphine	Base	Additive	H_2_O (equiv)	Yield [%] in **3**^a^

1^b^	**2a**	XPhos	*t-*BuONa	no additive	0	50
2^b^	**2a**	XPhos	NaH 60%	no additive	0	62
3^b^	**2a**	XPhos	NaH 60%	*t-*Bu-O-O-*t-*Bu	0	65
4^b^	**2b**	XPhos	NaH 60%	*t-*Bu-O-O-*t-*Bu	0	0
5^b^	**2a**	P(*t-*Bu)_3_	NaH 60%	*t-*Bu-O-O-*t-*Bu	0	51
6^b^	**2b**	P(*t-*Bu)_3_	NaH 60%	*t-*Bu-O-O-*t-*Bu	0	19
7^b^	**2a**	*t-*BuXPhos	NaH 60%	*t-*Bu-O-O-*t-*Bu	0	27
8^b^	**2b**	*t-*BuXPhos	NaH 60%	*t-*Bu-O-O-*t-*Bu	0	26
9^b^	**2a**	*t-*BuXPhos	Cs_2_CO_3_	*t-*Bu-O-O-*t-*Bu	0	62
10^c,d^	**2a**	*t-*BuXPhos	Cs_2_CO_3_	*t-*Bu-O-O-*t-*Bu	0	64
11^c,d^	**2a**	*t-*BuXPhos	Cs_2_CO_3_	*t-*Bu-O-O-*t-*Bu	2	71
12^c,d^	**2c**	*t-*BuXPhos	Cs_2_CO_3_	*t-*Bu-O-O-*t-*Bu	0	4
13^c,d^	**2c**	*t-*BuXPhos	Cs_2_CO_3_	*t-*Bu-O-O-*t-*Bu	2	69
14^c,d,e,f^	**2a**	*t-*BuXPhos	Cs_2_CO_3_	no additive	2	up to 77 (75%)
15 ^c,d,e,f,g^	**2a**	*t-*BuXPhos	Cs_2_CO_3_	no additive	2	71
16 ^c,d,e,f,h^	**2a**	*t-*BuXPhos	Cs_2_CO_3_	no additive	2	72

^a^Yields determined by GC/FID analysis using tetradecane as an internal standard, with isolated yields shown in parentheses. ^b^Reactions performed on a 0.5 mmol scale in 1 mL of DME. ^c^Reactions performed in 1 mL of DME (1 M). ^d^New batch of Cs_2_CO_3_ dry. ^e^Under O_2_ atmosphere (100 mL, 0.84 mmol). ^f^2 h. ^g^Using 1.5 equiv of **1a**.^. h^Using 1.5 equiv of **2a**.

**Scheme 1:**
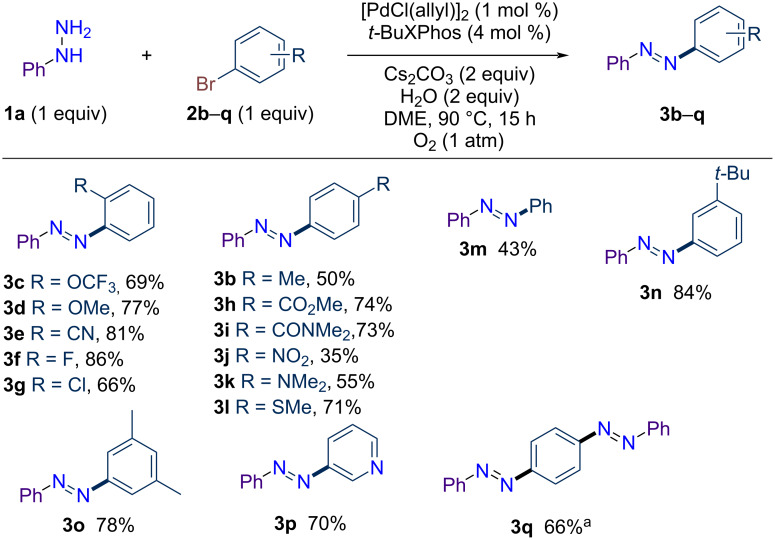
Scope of aryl bromides in palladium-catalyzed dehydrogenative C–N coupling with phenylhydrazine (**1a**). ^a^Using 2 equiv of **1a**.

With the most suitable reaction conditions in hand, the substrate scope was examined. First, the reactivity of different aryl bromides with phenylhydrazine (**1a**) to form non-symmetric azobenzene was explored (Scheme 1). Most of the azobenzenes were obtained in purified yields between 70 and 85%. Various functional groups at the *ortho*- or *para*-position were well tolerated in this reaction, including electron-withdrawing groups such as trifluoromethoxy (**3c**, 69%), nitrile (**3e**, 81%), methyl ester (**3h**, 74%), *N,N*-dimethylamide (**3i**, 73%) and nitro (**3j**, 35%), as well as electron-donating groups such as methoxy (**3d**, 77%), dimethylamino (**3k** 55%) , and thiomethoxy (**3l**, 71%). Moreover, the reaction tolerated C–F (**3f**, 86%) and C–Cl (**3g**, 66%) bonds, enabling orthogonal functionalization. It should be noted that, as a general trend – and in contrast to classic Buchwald–Hartwig couplings – aryl bromides with substituents at the *ortho*-position (**3a** and **3c**–**g**) are more reactive than those with substituents at the *para*-position (**3b** and **3h**–**l**). This difference might be explained by steric repulsion, which may favor C–N-bond coupling with the terminal nitrogen over the internal nitrogen. From phenylhydrazine and phenyl bromide, azobenzene (**3m**) was isolated in 43% yield. Substituent placed at the *meta*-position did not affect the reaction yield, as azobenzenes **3n** and **3o** are isolated in 84% and 78% yield, respectively. Interestingly, 3-bromopyridine also proved to be a suitable coupling partner, enabling the preparation of azoarene **3p** in 70% yield. Moreover, starting from 1,4-dibromobenzene and 2 equivalents of **1a**, a double reaction occurred, enabling the one-step synthesis of 1,4-bis[(*E*)-2-phenyldiazenyl]benzene (**3q**) in high yield.

**Scheme 2:**
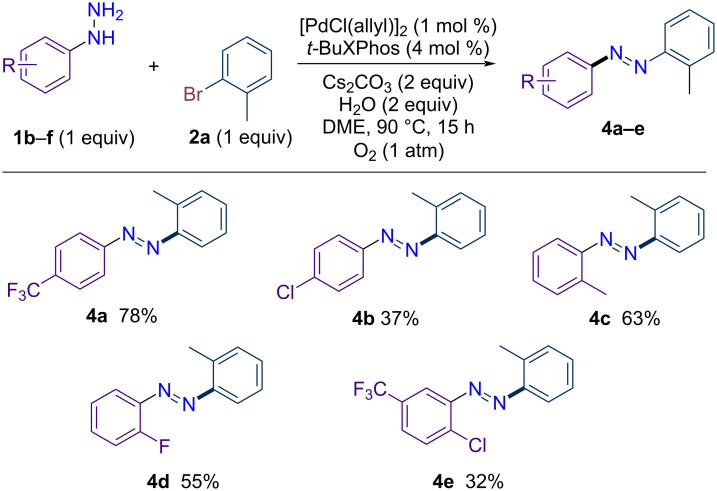
Scope of arylhydrazines in palladium-catalyzed dehydrogenative C–N coupling with 2-bromotoluene (**2a**).

We next investigated the scope of arylhydrazines to assess their compatibility under the optimized reaction conditions (Scheme 2). While some arylhydrazines are commercially available, it was observed that their hydrochloride salts were ineffective, even with the addition of excess base. Examining the substituent effects, *para*-substituted hydrazines such as *p*-(trifluoromethyl)phenylhydrazine (**1b**) and *p*-chlorophenylhydrazine (**1c**) reacted efficiently with 2-bromotoluene to deliver the corresponding azobenezenes **4a** and **4d** in 78% and 37% yield, respectively. Notably, 2-tolylhydrazine (**1d**) exhibited good reactivity, yielding **4c** in 63%. Additionally, 2-fluorophenylhydrazine provided the product in 55% yield, while 2-chloro-5-(trifluoromethyl)phenylhydrazine furnished the desired compound **4e** in a moderate yield of 32%. The lower yields observed with substrates containing a C–Cl bond may be attributed to the competitive Pd-catalyzed side-reaction.

**Scheme 3:**
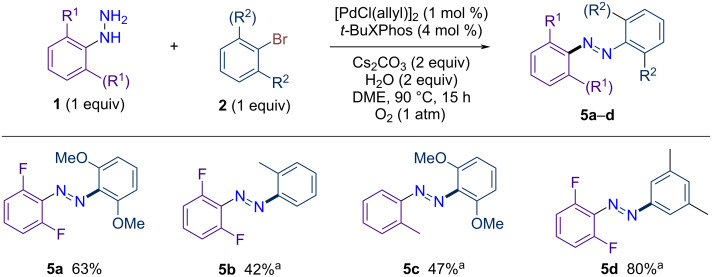
Application to the synthesis of tetra-, tri or di-*ortho*-substituted azobenzenes via palladium-catalyzed dehydrogenative C–N coupling. ^a^Isolated with 85–95% purity.

As shown above, the steric hindrance plays a significant role in driving the reaction. For this reason, we have applied our novel Pd methodology to synthesize a series of tetra-, tri-, and di-*ortho*-substituted azobenzenes (Scheme 3). Notably, this method demonstrates remarkable efficiency, as the reaction proceeds smoothly even when both coupling partners possess sterically hindered substituents at the 2,6-positions. For example, the coupling of 2,6-difluorophenylhydrazine with 1-bromo-2,6-dimethoxybenzene yielded tetra-*ortho*-substituted azobenzene **5a** in a notable yield of 63%, surpassing the performance of traditional approaches in similar contexts. Similarly, tri-*ortho*-substituted azobenzenes **5b** and **5c** were prepared in 42% and 47% yields, respectively. However, the isolation of these compounds in pure form remains challenging due to contamination with biphenyl side-products. Finally, di-*ortho*-substituted azobenzene **5d** was also successfully prepared using this approach, achieving an excellent yield of 80%.

**Figure 2:**
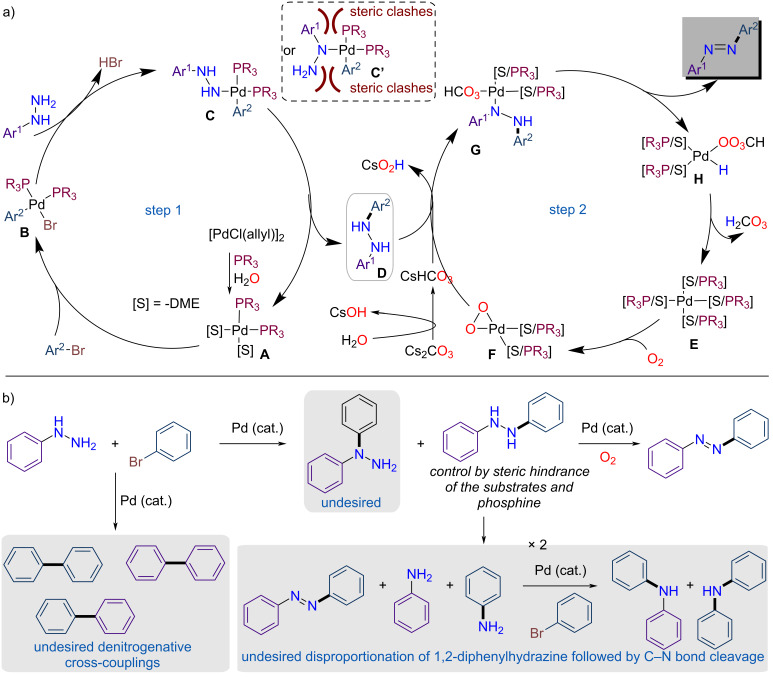
a) Proposed catalytic cycle for the one-pot palladium-catalyzed dehydrogenative C–N coupling for the synthesis of azobenzene from arylhydrazine and aryl bromide. b) Proposed chemical pathway for azobenzene synthesis, including side-product formation as determined by GC/MS analysis.

The mechanism of the reaction was further outlined to explain the formation of all identified main and side-products (Figure 2). A two-step cascade Pd-catalyzed reaction is proposed. In the first step a C–N-coupling reaction with phenylhydrazine occurs [57]. The catalytic cycle starts by the formation of Pd(0) in situ through reduction facilitated by phosphine and water [58]. This is followed by the oxidative addition of aryl bromides, leading to the formation of the Pd(II)–aryl intermediate (**B**). Subsequently, ligand exchange occurs, generating hydrazido complexes **C** and **C'**. When bulky substituents are present on the phosphine ligand and/or (both) coupling partner(s) has *ortho*-substituent(s), the hydrazido complex **C**, chelating on the terminal nitrogen, is preferentially formed to minimize steric clashes. Finally, reductive elimination leads to the formation of *N*,*N*-diarylhydrazine (**D**), which has been identified and characterized during the optimization process (see Supporting Information File 1 for details). In a second catalytic cycle, the *N*,*N*-diarylhydrazine (**D**) undergoes dehydrogenation via a mechanism involving Pd and O_2_, similar to the process reported by Huang and co-workers [59]. Initially, Pd(0) species **E** is oxidized to Pd(II) by O_2_, forming a Pd-peroxo complex **F** [60]. Subsequently, ligand exchange occurs between the deprotonated *N*,*N*-diarylhydrazine and carbonate, yielding the Pd(II) intermediate **G**. This intermediate then undergoes β–H elimination to afford the desired azobenzene product, along with a Pd(II) species **H**. Finally, reductive elimination regenerates Pd(0), completing the catalytic cycle. Then, a general reaction pathway for the formation of product and side-products is presented in Figure 2B. The first reaction to be inhibited is the Pd-catalyzed denitrogenative cross-coupling, which leads to the formation of an array of biphenyl products [52–54]. This can be controlled by selecting appropriate catalysts and solvents. For instance, PdCl(allyl)_2_ in DME with strong base (Cs_2_CO_3_) favors C–N-bond coupling, which may yield products resulting from arylation at either the terminal or internal nitrogen atoms. The selectivity can be influenced by the steric hindrance of the phosphine ligands and/or the substrates. Once *N*,*N*-diarylhydrazine is formed, minimizing the disproportionation side-reaction [61] becomes crucial, as this reaction produces the desired azobenzene along with equimolar amounts of aniline partners. These aniline derivatives can further participate in Pd-catalyzed cross-coupling, generating a range of diarylamines as side-products. The presence of oxidants such as O_2_ mitigates this pathway by promoting oxidative dehydrogenation as the dominant pathway.

## Conclusion

In summary, we have developed robust and efficient conditions for the preparation of azobenzenes via C–N coupling and dehydrogenation, employing [PdCl(C_3_H_5_)]_2_ with *t-*BuXPhos to promote selective C–N-bond formation. This approach makes use of commercially available starting materials and displays broad functional group tolerance. Notably, it enables access to sterically demanding tetra-*ortho*-substituted azobenzenes in moderate to good yields. The results demonstrate that arylhydrazines can serve as practical amine partners in Pd-catalyzed C–N coupling reactions, with regioselectivity toward arylation of the less nucleophilic terminal nitrogen governed by the steric profile of the substrates and the choice of phosphine ligand. These conditions represent a valuable addition to existing methodologies and open further opportunities for applications in molecular probe design, functional materials, and photoresponsive systems, where *ortho*-substitution is often critical.

## Supporting Information

File 1 Details of optimization experiments, full characterization data, and NMR spectra (^1^H, ^13^C, ^19^F) for all products.

## Data Availability

All data that supports the findings of this study is available in the published article and/or the supporting information of this article.

## References

[1] Yesodha S K, Sadashiva Pillai C K, Tsutsumi N (2004). Prog Polym Sci.

[2] Bandara H M D, Burdette S C (2012). Chem Soc Rev.

[3] Marturano V, Ambrogi V, Bandeira N A G, Tylkowski B, Giamberini M, Cerruti P (2017). Phys Sci Rev.

[4] Tylkowski B, Trojanowska A, Marturano V, Nowak M, Marciniak L, Giamberini M, Ambrogi V, Cerruti P (2017). Coord Chem Rev.

[5] Crespi S, Simeth N A, König B (2019). Nat Rev Chem.

[6] Jerca F A, Jerca V V, Hoogenboom R (2022). Nat Rev Chem.

[7] Dudek M, Kaczmarek-Kędziera A, Deska R, Trojnar J, Jasik P, Młynarz P, Samoć M, Matczyszyn K (2022). J Phys Chem B.

[8] Xu X, Feng J, Li W-Y, Wang G, Feng W, Yu H (2024). Prog Polym Sci.

[9] Beharry A A, Woolley G A (2011). Chem Soc Rev.

[10] Szymański W, Beierle J M, Kistemaker H A V, Velema W A, Feringa B L (2013). Chem Rev.

[11] Cheng H-B, Zhang S, Qi J, Liang X-J, Yoon J (2021). Adv Mater (Weinheim, Ger).

[12] Merino E (2011). Chem Soc Rev.

[13] Kurup S S, Groysman S (2022). Dalton Trans.

[14] Zhang C, Jiao N (2010). Angew Chem, Int Ed.

[15] Takeda Y, Okumura S, Minakata S (2012). Angew Chem, Int Ed.

[16] Cai S, Rong H, Yu X, Liu X, Wang D, He W, Li Y (2013). ACS Catal.

[17] Singh S, Chauhan P, Ravi M, Taneja I, Wahajuddin W, Yadav P P (2015). RSC Adv.

[18] Wang M, Ma J, Yu M, Zhang Z, Wang F (2016). Catal Sci Technol.

[19] Damiano C, Cavalleri M, Panza N, Gallo E (2022). Eur J Org Chem.

[20] Hu L, Cao X, Shi L, Qi F, Guo Z, Lu J, Gu H (2011). Org Lett.

[21] Sakai N, Asama S, Anai S, Konakahara T (2014). Tetrahedron.

[22] Zhang Y-F, Mellah M (2017). ACS Catal.

[23] Ma Y, Wu S, Jiang S, Xiao F, Deng G-J (2021). Chin J Chem.

[24] Grirrane A, Corma A, García H (2008). Science.

[25] Mills C (1895). J Chem Soc, Trans.

[26] Griwatz J H, Kunz A, Wegner H A (2022). Beilstein J Org Chem.

[27] Griwatz J H, Campi C E, Kunz A, Wegner H A (2024). ChemSusChem.

[28] Kunz A, Oberhof N, Scherz F, Martins L, Dreuw A, Wegner H A (2022). Chem – Eur J.

[29] Haghbeen K, Tan E W (1998). J Org Chem.

[30] Merrington J, James M, Bradley M (2002). Chem Commun.

[31] Esguerra K V N, Lumb J-P (2017). Chem – Eur J.

[32] Barbero M, Degani I, Dughera S, Fochi R, Perracino P (1998). Synthesis.

[33] Hansen M J, Lerch M M, Szymanski W, Feringa B L (2016). Angew Chem, Int Ed.

[34] Kunz K, Scholz U, Ganzer D (2003). Synlett.

[35] Schlummer B, Scholz U (2004). Adv Synth Catal.

[36] Beletskaya I P, Cheprakov A V (2012). Organometallics.

[37] Bariwal J, Van der Eycken E (2013). Chem Soc Rev.

[38] Ruiz-Castillo P, Buchwald S L (2016). Chem Rev.

[39] Forero-Cortés P A, Haydl A M (2019). Org Process Res Dev.

[40] Emadi R, Bahrami Nekoo A, Molaverdi F, Khorsandi Z, Sheibani R, Sadeghi-Aliabadi H (2023). RSC Adv.

[41] Wang Y, Xie R, Huang L, Tian Y-N, Lv S, Kong X, Li S (2021). Org Chem Front.

[42] Lim Y-K, Lee K-S, Cho C-G (2003). Org Lett.

[43] Finck L, Oestreich M (2022). Angew Chem, Int Ed.

[44] Chauvier C, Finck L, Hecht S, Oestreich M (2019). Organometallics.

[45] Xu Y, Gao C, Andréasson J, Grøtli M (2018). Org Lett.

[46] Lv H, Laishram R D, Yang Y, Li J, Xu D, Zhan Y, Luo Y, Su Z, More S, Fan B (2020). Org Biomol Chem.

[47] Tuck J R, Tombari R J, Yardeny N, Olson D E (2021). Org Lett.

[48] Lin Y, Wu H, Liu Z, Li J, Cai R, Hashimoto M, Wang L (2022). Tetrahedron Lett.

[49] Orvoš J, Pančík F, Fischer R (2023). Eur J Org Chem.

[50] Phadnis N, Molen J A, Stephens S M, Weierbach S M, Lambert K M, Milligan J A (2024). J Org Chem.

[51] Kocúrik M, Konopáčová P, Kolman L, Kryl P, Růžička A, Bartáček J, Hanusek J, Váňa J (2024). ACS Omega.

[52] Rao H, Jin Y, Fu H, Jiang Y, Zhao Y (2006). Chem – Eur J.

[53] Huang Y, Choy P Y, Wang J, Tse M-K, Sun R W-Y, Chan A S-C, Kwong F Y (2020). J Org Chem.

[54] Sudharsan M, Thirumoorthy K, Nethaji M, Suresh D (2019). ChemistrySelect.

[55] Ikawa T, Barder T E, Biscoe M R, Buchwald S L (2007). J Am Chem Soc.

[56] Fors B P, Krattiger P, Strieter E, Buchwald S L (2008). Org Lett.

[57] Wang J Y, Choi K, Zuend S J, Borate K, Shinde H, Goetz R, Hartwig J F (2021). Angew Chem, Int Ed.

[58] DeAngelis A J, Gildner P G, Chow R, Colacot T J (2015). J Org Chem.

[59] Gao W, He Z, Qian Y, Zhao J, Huang Y (2012). Chem Sci.

[60] Steinhoff B A, Fix S R, Stahl S S (2002). J Am Chem Soc.

[61] Karnbrock S B H, Golz C, Mata R A, Alcarazo M (2022). Angew Chem, Int Ed.

