# Supplementary material for: Pd-catalyzed dehydrogenative arylation of arylhydrazines to access non-symmetric azobenzenes, including tetra-ortho derivatives
Source: Beilstein J Org Chem. 2025 Oct 22;21:2234–42. doi: 10.3762/bjoc.21.170 (PMC12557439; doi:10.3762/bjoc.21.170)
Supplement: File 1 — Details of optimization experiments, full characterization data, and NMR spectra (1H, 13C, 19F) for all products. [file Beilstein_J_Org_Chem-21-2234-s001.pdf]

BEILSTEIN JOURNAL OF ORGANIC CHEMISTRY

# Supporting Information

for

# Pd-catalyzed dehydrogenative arylation of arylhydrazines to access non-symmetric azobenzenes, including tetra-*ortho* derivatives

Loris Geminiani, Kathrin Junge, Matthias Beller and Jean-François Soulé

*Beilstein J. Org. Chem.* **2025,** *21,* 2234–2242. doi:10.3762/bjoc.21.170

# Details of optimization experiments, full characterization data, and NMR spectra ($^1$H, $^{13}$C, $^{19}$F) for all products

License and Terms: This is a supporting information file under the terms of the Creative Commons Attribution License (https://creativecommons.org/licenses/by/4.0). Please note that the reuse, redistribution and reproduction in particular requires that the author(s) and source are credited and that individual graphics may be subject to special legal provisions.
The license is subject to the *Beilstein Journal of Organic Chemistry* terms and conditions: (https://www.beilstein-journals.org/bjoc/terms)

## Table of contents

I. General information ........ S2

II. Additional experiments on reaction optimization ........ S3

III. Experimental procedures and characterization data ........ S8

IV. $^{1}H$, $^{13}C$ and $^{19}F$ NMR spectra: ........ S23

# I. General information

Air-sensitive syntheses were performed under argon atmosphere, air- and moisture-sensitive syntheses were performed under argon atmosphere in heating gun vacuum-dried glassware. Chemicals were purchased from Aldrich, TCI, BLDpharm, ABCR, enamine, activate scientific and Strem chemicals. Argon was provided by Linde Europe Solvents and conditions under argon conditions were degassed prior to use.

The products were characterized by $^{1}$H NMR, $^{13}$C and $^{19}$F spectroscopy. $^{1}$H, $^{13}$C and $^{19}$F NMR spectra were recorded on a Bruker Avance 300 (300 MHz), 400 (400 MHz) Fourier-300 (300 MHz) NMR spectrometer. Chemical shifts δ (ppm) are given relative to solvent: references for $CDCl_3$ were 7.26 ppm ($^{1}$H NMR) and 77.16 ppm ($^{13}$C NMR); references for MeOH-$d_4$ were 3.31 ppm ($^{1}$H NMR) and 49.00 ppm ($^{13}$C NMR); references for DMSO-$d_6$ were 2.50 ppm ($^{1}$H NMR) and 39.52 ($^{13}$C). $^{13}$C NMR spectra were acquired in broad band-decoupled mode. Multiplets were assigned as s (singlet), d (doublet), t (triplet), q (quadruplet) and m (multiplet).

GC/MS analyses were performed using an Agilent 8890 GC instrument and a 5977B GC/MSD instrument using an Agilent HP-5MS column. The oven program was as follows: 50 °C for 0 min, then 8 °C/min to 120 °C, then 15 °C/min to 200 °C, then 25 °C/min to 300 °C, then 15 min at 300 °C.

GC/FID and GC/MS yields for optimization were calculated considering that all azo-compounds have the same answer factor than azobenzene using tetradecane as an internal standard.

According to GC/MS analysis, the following products are formed but in trace amounts in most of the reactions even if not reported:

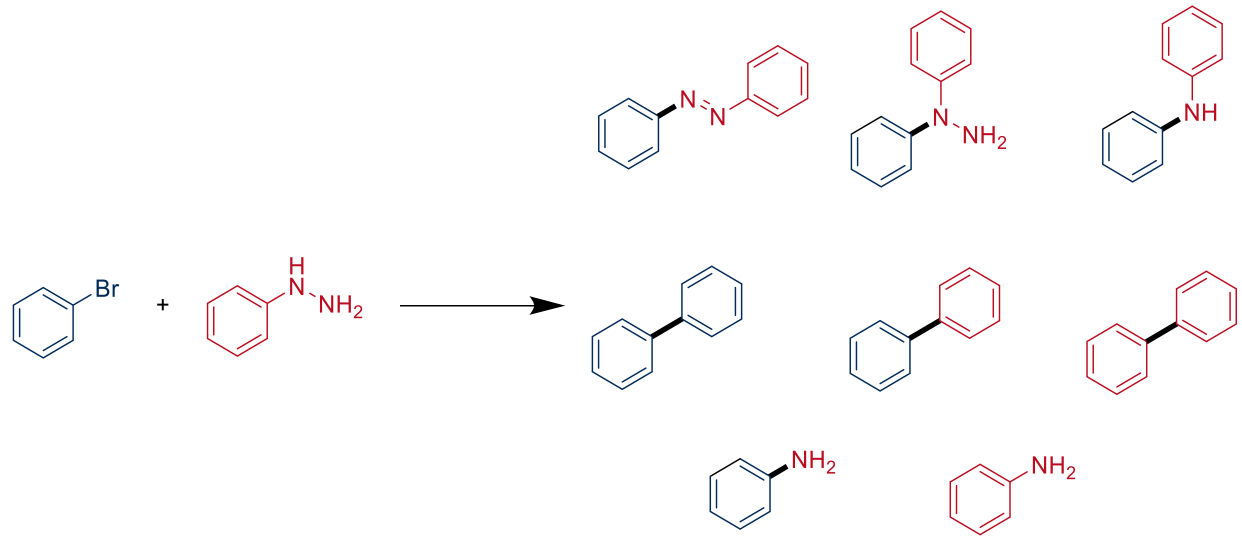

## II. Additional experiments on reaction optimization

**Table S1. Choice of phosphines (1)**

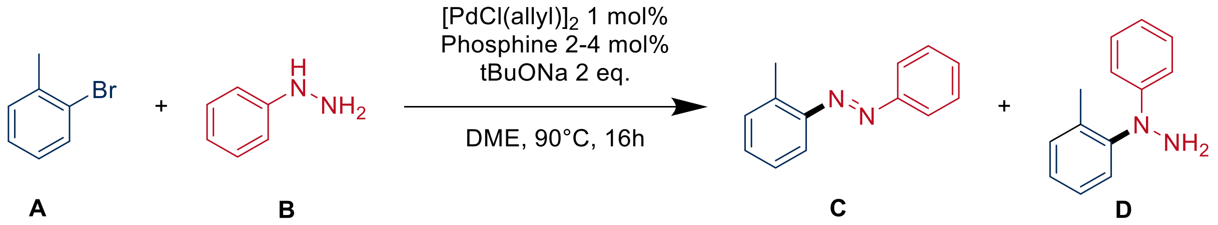


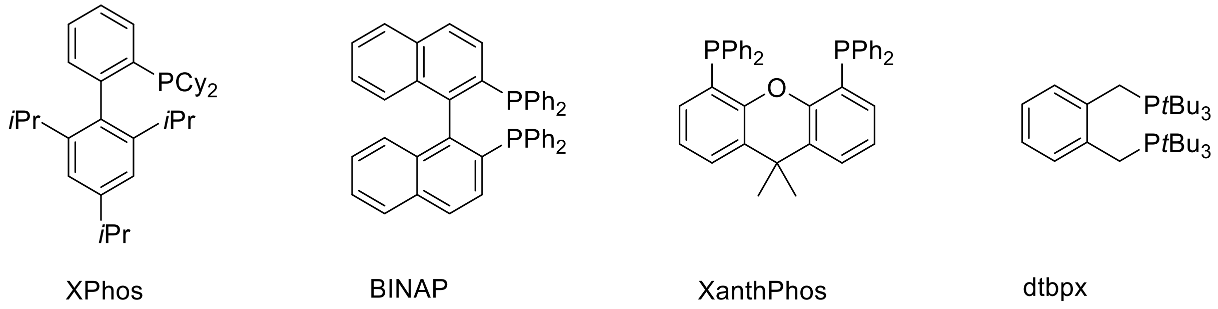


| Entry | Phosphine | A azobenzene / A dodecane (internal standard), GC/MS |
|---|---|---|
| **1** | **XPhos 4 mol %** | **0.67 / 1** |
| **2** | **BINAP 2 mol %** | **0.66 / 1** |
| 3 | XanthPhos | 0.24 / 1 |
| 4 | dtbpx | 0.14 / 1 |
| Reaction conditions : 2-bromotoluene (0.5 mmol, 60 µL), phenylhydrazine (0.5 mmol (49 µL), $[PdCl(allyl)]_2$ (5 µmol, 1.9 mg), phosphine (20–40 µmol), *t*-BuONa (1 mmol), DME (1 mL), 90 °C, 16 h | | |

**Table S2. Choice of base (1)**

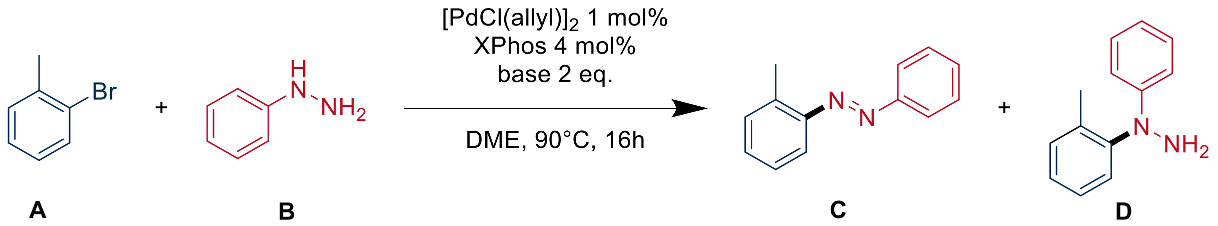


| Entry | Base | GC/MS yield of C | Area of C(GC/MS)/ Area of D (GC/MS) |
|---|---|---|---|
| 5 | *t*-BuONa | 50 | 0042 |
| 6 | $K_2CO_3$ | 0 | Not determined |
| 7 | KOH | 0 | Not determined |
| 8 | $K_3PO_4$ | 25 | Not determined |
| **9** | **NaH 60 wt %** | **62** | **0.068** |
| 10 | KOAc | 1 | Not determined |
| 11 | AgOAc | 0 | Not determined |
| Reaction conditions : 2-bromotoluene (0 .5 mmol, 60 µL), phenylhydrazine (0.5 mmol) (49 µL), $[PdCl(allyl)]_2$ (5 µmol, 1.9 mg), XPhos (20 µmol, 9.5 mg), base (1 mmol), DME (1 mL), 90 °C, 16 h | | | |

**Table S3. Test of additives**

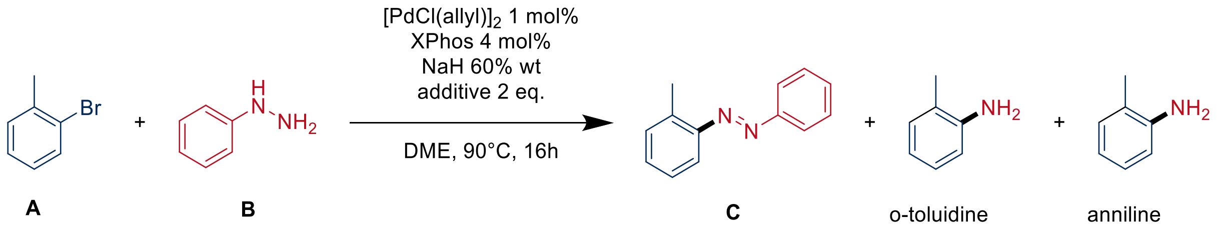


| Entry | Additive | GC/FID yield of C | GC/FID yield of *o*-toluidine | GC/FID yield of anniline |
|---|---|---|---|---|
| 12 | No additive | 31 | 34 | 44 |
| 13 | cyclohexene | 52 | 45 | 46 |
| **14** | ***t*-Bu-O-O-*t*-Bu** | **65** | **2** | **3** |
| **15** | **DCC** | **65** | **8** | **5** |
| 16 | Ph-$CO_2$-O-*t*Bu | 30 | 24 | 26 |
| Reaction conditions : 2-bromotoluene (0 .5 mmol, 60 µL), phenylhydrazine (0.5 mmol (49 µL), $[PdCl(allyl)]_2$ (5 µmol, 1.9 mg), XPhos (20 µmol, 9.5 mg), *t*-BuONa (1 mmol, 96 mg), additive (1 mmol) DME (1 mL), 90 °C, 16 h | | | | |

**Table S4. Importance of all reagents**

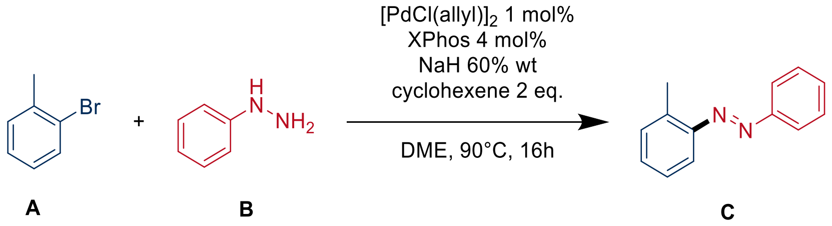


| Entry | $[PdCl(allyl)]_2$ | XPhos | NaH 60 wt % | Cyclohexene | Yield of C |
|---|---|---|---|---|---|
| **17** | ✓ | ✓ | ✓ | ✓ | **46** |
| 18 | ✗ | ✓ | ✓ | ✓ | 1 |
| 19 | ✗ | ✗ | ✓ | ✓ | 2 |
| 20 | ✓ | ✓ | ✗ | ✓ | 0 |
| 21 | ✗ | ✗ | ✓ | ✗ | 0 |
| Reaction conditions : 2-bromotoluene (0 .5 mmol, 60 µL), phenylhydrazine (0.5 mmol(49 µL), $[PdCl(allyl)]_2$ (5 µmol, 1.9 mg), XPhos (20 µmol, 9.5 mg), *t*-BuONa (1 mmol, 96 mg), cyclohexene (1 mmol) DME (1 mL), 90 °C, 16 h | | | | | |

**Table S5. Optimization of phosphine (2), comparison with different aryl bromides**

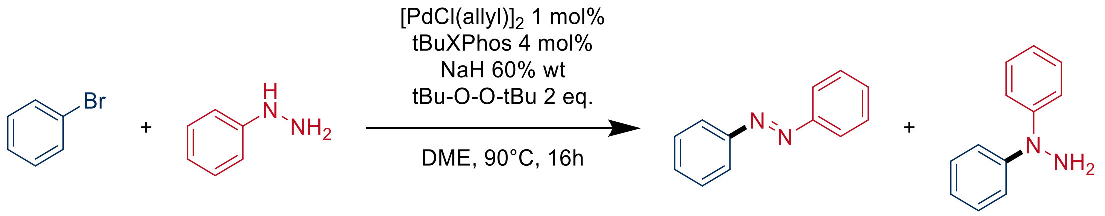


A B C D

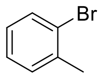


2-bromotoluene

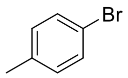


4-bromotoluene

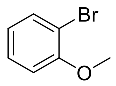


2-bromoanisole

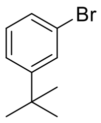


3-bromotertbutylbenzene

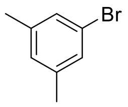


3,5-dimethylbromobenzene

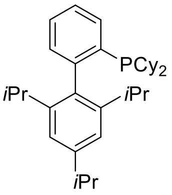


XPhos

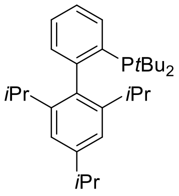


tBuXPhos

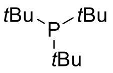


$PtBu_3$

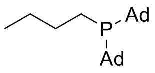


Cataxcium

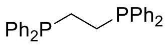


dppe

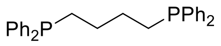


dppb

| Entry | Aryl bromide | Phosphine | GC/FID yield of azobenzene |
|---|---|---|---|
| **22** | **2-bromotoluene** | **XPhos** | **54** |
| 23 | 2-bromotoluene | *t*-BuXPhos | 27 |
| **24** | **2-bromotoluene** | **P*t*-Bu$_3$** 10 wt % in hexane | **51** |
| 25 | 4-bromotoluene | XPhos | **0** |
| **26** | **4-bromotoluene** | ***t*-BuXPhos** | **26** |
| 27 | 4-bromotoluene | CatacXium | 0 |
| 28 | 4-bromotoluene | Dppb | 0 |
| 29 | 4-bromotoluene | Dppe | 0 |
| 30 | 4-bromotoluene | P-*t*Bu$_3$ 10 wt % in hexane | 19 |
| 31 | 2-bromoanisole | XPhos | 0 |
| **32** | **2-bromoanisole** | ***t*-BuXPhos** | **64** |
| 33 | 2-bromoanisole | P-*t*Bu$_3$ 10 wt % in hexane | 33 |
| 34 | 3-bromo-*tert*-butylbenzene | XPhos | 0 |
| **35** | **3-bromo-*tert*-butylbenzene** | ***t*-BuXPhos** | **45** |
| 36 | 3-bromo-*tert*-butylbenzene | P*t*Bu$_3$ 10 wt % in hexane | 28 |
| 37 | 3,5-dimethylbromobenzene | XPhos | 0 |
| **38** | **3,5-dimethylbromobenzene** | ***t*-BuXPhos** | **25** |
| 39 | 3,5-dimethylbromobenzene | P*t*-Bu$_3$ 10 wt % in hexane | 24 |

Reaction conditions : aryl bromide (0.5 mmol, 60 µL), phenylhydrazine (0.5 mmol) (49 µL), [PdCl(allyl)]$_2$ (5 µmol, 1.9 mg), phosphine (20 µmol, 9.5 mg), NaH 60% wt (0.5 mmol, 40 mg), *t*-Bu-O-O-*t*-Bu (1 mmol, 184 µL) DME (1 mL), 90 °C, 16 h

**Table S6. Scope of base (2)**

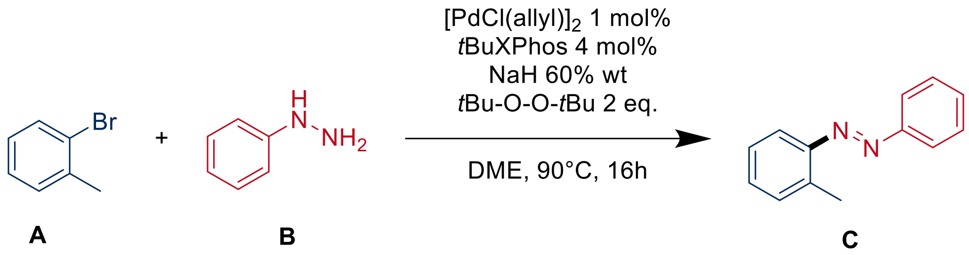


| Entry | Base | GC/MS yield of C |
|---|---|---|
| 40 | NaH 60 wt % | 38 |
| 41 | *t*-BuONa | 9 |
| 42 | *t*-BuOK | 10 |
| 43 | AcONa | 0 |
| 44 | $K_2CO_3$ | 7 |
| **45** | **$Cs_2CO_3$** | **62** |
| 46 | $K_3PO_4$ | 43 |
| Reaction conditions : 2-bromotoluene (0 .5 mmol, 60 µL), phenylhydrazine (0.5 mmol (49 µL), $[PdCl(allyl)]_2$ (5 µmol, 1.9 mg), *t*-BuXPhos (20 µmol, 8.5 mg), base (1 mmol), DME (1 mL), 90 °C, 16 h | | |

**Table S7 Role of water**

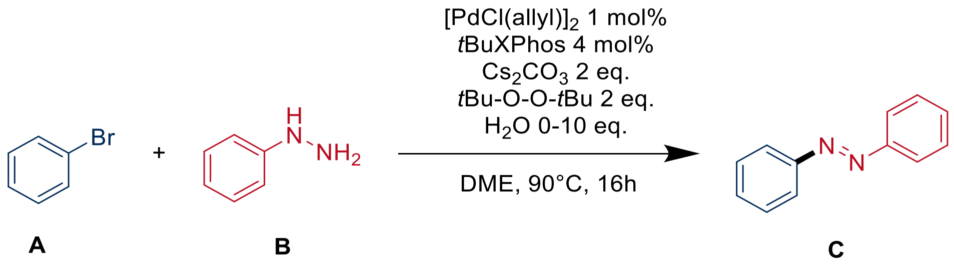


| Entry | Aryl bromide | Amount of $H_2O$ | GC/FID yield of azobenzene |
|---|---|---|---|
| 47 | 2-bromotoluene | 0 | 64 |
| 48 | 2-bromotoluene | 0.5 | 70 |
| **49** | **2-bromotoluene** | **1** | **74** |
| **50** | **2-bromotoluene** | **2** | **71** |
| 51 | 2-bromotoluene | 10 | 13 |
| **52** | 1-bromo-2(trifluoromethoxy)benzene | 0 | 4 |
| **53** | 1-bromo-2(trifluoromethoxy)benzene | 0.5 | 28 |
| **54** | 1-bromo-2(trifluoromethoxy)benzene | 1 | 51 |
| **55** | **1-bromo-2(trifluoromethoxy)benzene** | **2** | **69** |
| **56** | 1-bromo-2(trifluoromethoxy)benzene | 10 | 10 |
| Reaction conditions : aryl bromide (1 mmol, 120 µL), phenylhydrazine (1 mmol, 98 µL), $[PdCl(allyl)]_2$ (10 µmol, 3.7 mg), *t*-BuXPhos (20 µmol, 17 mg), $Cs_2CO_3$ (2 mmol, 652mg), -*t*Bu-O-O-*t*-Bu (2 mmol, 368 µL), $H_2O$ (0–10 mmol) DME (1 mL), 90 °C, 16 h | | | |

**Table S8. Shorter reaction time**

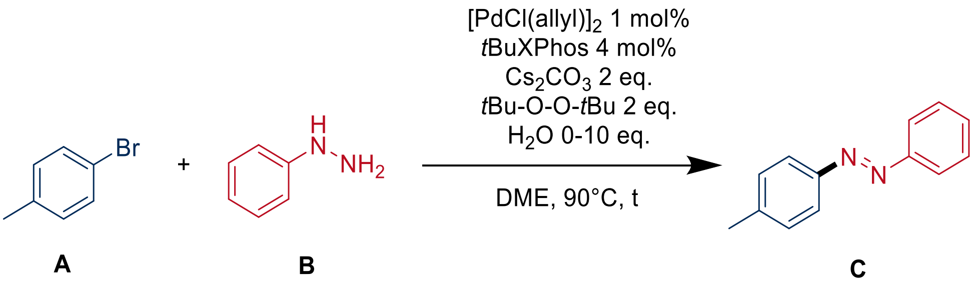


| Entry | Time | GC/FID yield of azobenzene |
|---|---|---|
| 57 | 2 minutes | 11 |
| 58 | 5 minutes | 13 |
| 59 | 2 hours | 42 |
| **60** | 16 hours | 46 |
| Reaction conditions : 2-bromotoluene (1 mmol, 120 µL), phenylhydrazine (1 mmol, 98 µL), $[PdCl(allyl)]_2$ (10 µmol, 3.7 mg), *t*-BuXPhos (20 µmol, 17 mg), $Cs_2CO_3$ (2 mmol, 652mg), *t*-Bu-O-O-*t*-Bu (2 mmol, 368 µL), $H_2O$ (2 mmol, 36 µL), DME (1 mL), 90 °C, *t* | | |

**Table S9. Comparison of aryl halides**

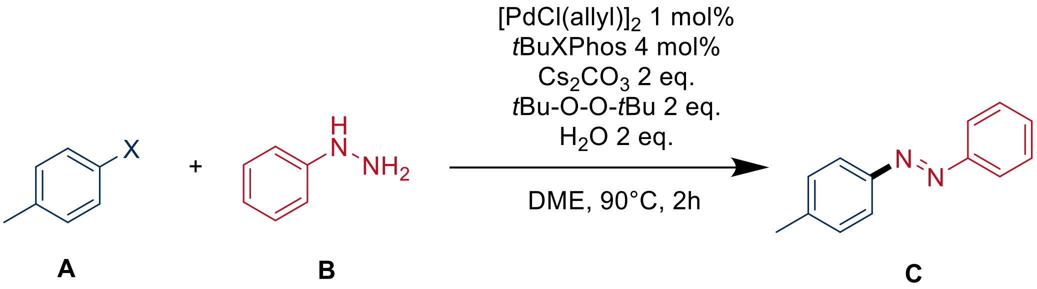


| Entry | X | GC/FID yield of azobenzene |
|---|---|---|
| 61 | Cl | 26 |
| **62** | **Br** | **52** |
| 63 | I | 22 |
| Reaction conditions : 2-halogenotoluene (1 mmol, 120 µL), phenylhydrazine (1 mmol, 98 µL), $[PdCl(allyl)]_2$ (10 µmol, 3.7 mg), *t*-BuXPhos (20 µmol, 17 mg), $Cs_2CO_3$ (2 mmol, 652mg), *t*-Bu-O-O-*t*-Bu (2 mmol, 368 µL), $H_2O$ (2 mmol, 36 µL), DME (1 mL), 90 °C, 2 h | | |

**Table S10. Role of dioxygen in the reaction, no influence of additive in presence of $O_2$**

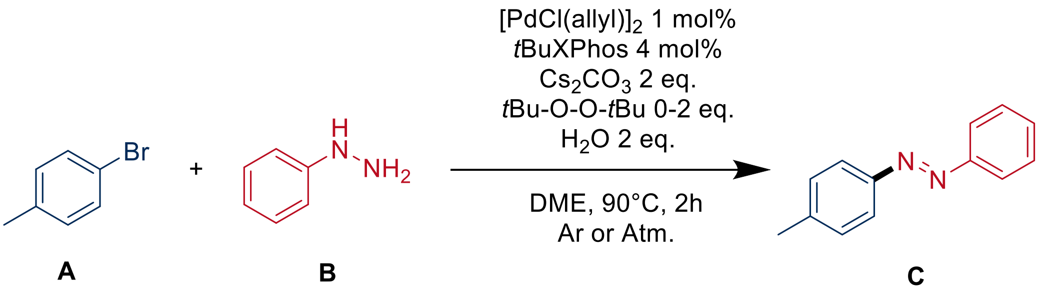


| Entry | *t*-Bu-O-O-*t*-Bu ? | Atmosphere | Volume of the schlenk (mL) | Amount of 02 (considering 25 °C, 1 atm.) | GC/FID yield of azobenzene |
|---|---|---|---|---|---|
| 64 | Yes | Ar | 25 | 0.21 | 13 |
| 65 | Yes | Atm | 25 | 0.21 | 47 |
| 66 | No | Ar | 25 | 0.21 | 43 |
| 67 | No | Atm | 25 | 0.21 | 43 |
| **68** | **No** | **Atm** | **50** | **0.42** | **53-78** |
| **69** | **No** | **Atm** | **100** | **0.84** | **69-77** |
| Reaction conditions : 2-bromotoluene (1 mmol, 120 µL), phenylhydrazine (1 mmol, 98 µL), [PdCl(allyl)]$_2$ (10 µmol, 3.7 mg), *t*-BuXPhos (20 µmol, 17 mg), $Cs_2CO_3$ (2 mmol, 652 mg), *t*-Bu-O-O-*t*-Bu (0–2 mmol), $H_2O$ (0–10 mmol) DME (1 mL), 90 °C, 16 h | | | | | |

# III. Experimental procedures and characterization data

**General procedure for the synthesis of azo compounds:**

In a 50 mL Schlenk flask open to ambient atmosphere (air, without inert gas) – *as the presence of oxygen is essential for the reaction* – equipped with a magnetic stirring bar, are added 3.7 mg of [PdCl(allyl)]$_2$ (0.01 mmol), 17 mg of *t*-BuXPhos (0.04 mmol), 652 mg of $Cs_2CO_3$. 1 mL of 1,2-dimethoxyethane, 36 µL of water (2 mmol), 1 mmol of aryl bromide and 1 mmol of phenylhydrazine derivatives are added. The Schlenk flask is then closed and the mixture stirred for 2 hours in an oil bath at 90 °C. After cooling to rt, water is added and the organic phase is extracted with ethyl acetate. The organic phase is then dried with $MgSO_4$, concentrated in vacuo and the crude product is purified on a flash chromatographic column (12 g $SiO_2$, "gold" quality, pentane/AcOEt) to afford the desired azo-compound.

In some case the *Z* azobenzene isomer is present and observed by NMR analysis.

All compounds are analyzed by $^{1}H$ and $^{13}C$ NMR ($^{19}F$ NMR analysis has also been performed for fluorine-containing molecules) and GC/MS.

HRMS were performed for the non-described compounds.

**Purification of phenylhydrazine**

In a 250 mL Erlenmeyer flask are added 29 mmol of phenylhydrazine hydrochloride derivative. 58 mmol of potassium hydroxide, 30 mL of dichloromethane and 30 mL of water are added and the solution is stirred for 15 minutes. The organic phase is recovered, dried over $MgSO_4$ and concentrated in vacuo to afford the pure phenylhydrazine derivative.

**Figure S1: Failed experiments**

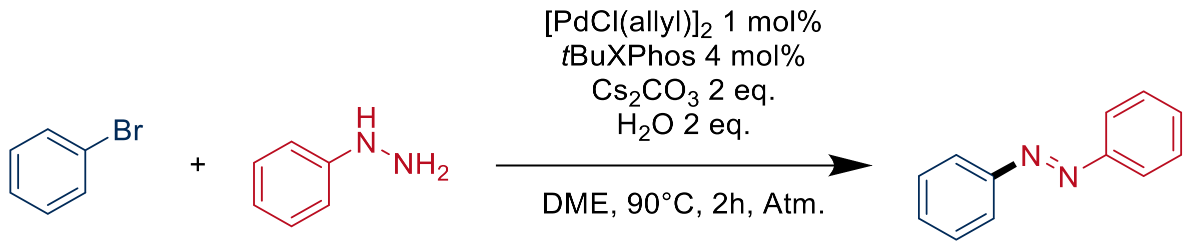


**A** **B** **C (not identified)**

Failed reactions, azobenzenes not identified

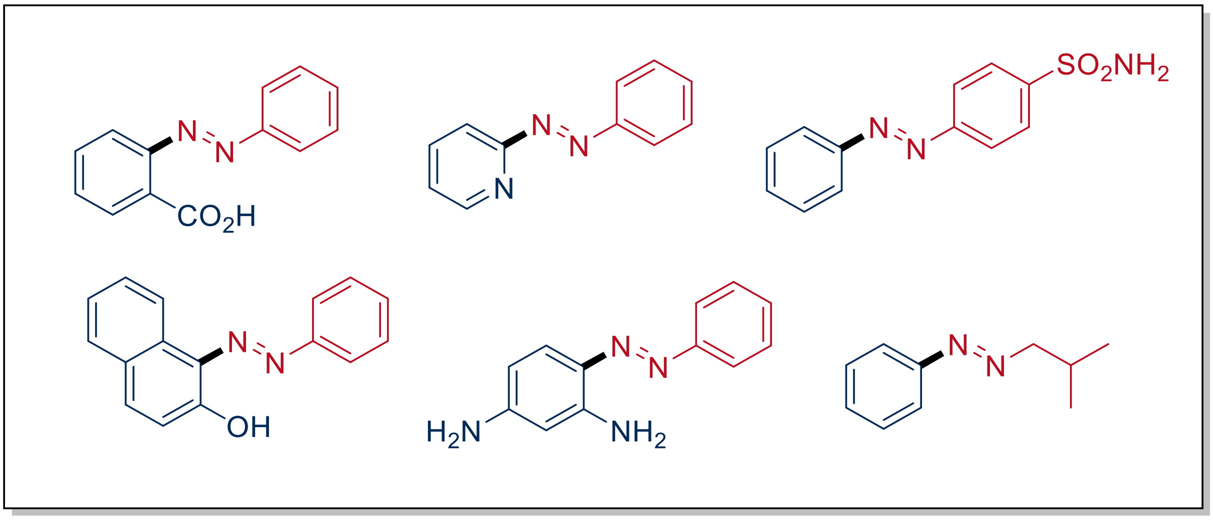

**(*E*)-1-Phenyl-2-(*o*-tolyl)diazene (3a)**

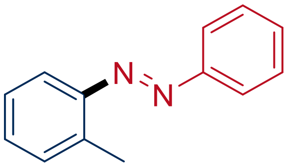


Prepared according to general procedure from phenylhydrazine (108.1 mg, 1 mmol, 1 equiv) and 2-bromotoluene (171.0 mg, 1 mmol, 1 equiv). After purification, 148 mg of **3a** are obtained (0.75 mmol, 75%). Around 5% of the *Z* isomer are observed according to $^1$H NMR.

**$^1$H NMR** (300 MHz, MeOD) δ 7.87 – 7.76 (m, 2H), 7.59 – 7.50 (m, 1H), 7.48 – 7.32 (m, 3H), 7.32 – 7.19 (m, 2H), 7.19 – 7.09 (m, 1H), 2.61 (s, 3H).

**{$^1$H}$^{13}$C NMR** (75 MHz, MeOD) δ 154.2, 151.7, 139.2, 132.2, 132.0, 131.9, 130.1, 127.3, 123.8, 116.2, 17.6.

The NMR data are consistent with those reported for the known compound (CAS 6676-90-0).

**(*E*)-1-Phenyl-2-(*p*-tolyl)diazene (3b)**

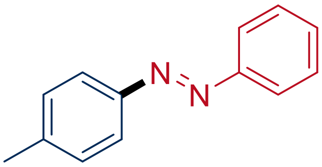


Prepared according to general procedure from phenylhydrazine (108.1 mg, 1 mmol, 1 equiv) and 4-bromotoluene (171.0 mg, 1 mmol, 1 equiv). After purification, 99 mg of **3b** was obtained (0.50 mmol, 50%). An additional 41 mg of **3b** (0.28 mmol, 28%) was recovered in a fraction containing biphenyl as an impurity. Around 5% of the *Z* isomer are observed according to $^1$H NMR.

**$^1$H NMR** (300 MHz, MeOD) δ 7.91 – 7.83 (m, 2H), 7.83 – 7.76 (m, 2H), 7.56 – 7.45 (m, 3H), 7.37 – 7.27 (m, 2H), 2.40 (s, 3H).

**{$^1$H}$^{13}$C NMR** (75 MHz, MeOD) δ 154.0, 152.0, 143.1, 131.9, 130.8, 130.2, 123.8, 123.6, 21.5.

The NMR data are consistent with those reported for the known compound (CAS 949-87-1).

**(*E*)-1-Phenyl-2-(2-(trifluoromethoxy)phenyl)diazene (3c)**

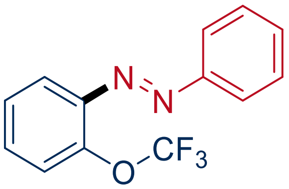


Prepared according to general procedure from phenylhydrazine (108.1 mg, 1 mmol, 1 equiv) and 2-trifluoromethoxybromobenzene (241.0 mg, 1 mmol, 1 equiv). After purification, 184 mg of **3c** are obtained (0.69 mmol, 69%). Around 10% of the *Z* isomer are observed according to $^{1}$H NMR.

**$^{1}$H NMR** (300 MHz, MeOD) δ 7.97 – 7.83 (m, 2H), 7.75 (dd, *J* = 8.0, 1.7 Hz, 1H), 7.59 – 7.37 (m, 6H).

**{$^{1}$H, $^{19}$F}$^{13}$C NMR** (75 MHz, MeOD) δ 154.1, 148.0, 148.0, 146.2, 133.4, 132.9, 130.3, 129.1, 124.2, 124.1, 118.4.

**{$^{1}$H}$^{19}$F NMR** (282 MHz, MeOD) δ -59.0.

**HRMS** (ESI) theoretical mass for [M+H]: 267.0740; found: 267.0746.

**(*E*)-1-(2-Methoxyphenyl)-2-phenyldiazene (3d)**

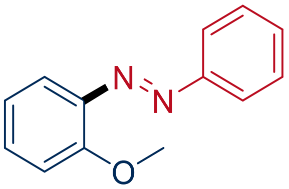


Prepared according to general procedure from phenylhydrazine (108.1 mg, 1 mmol, 1 equiv) and 2-bromoanisole (187.0 mg, 1 mmol, 1 equiv). After purification, 163 mg of **3d** was obtained (0.77 mmol, 77%). An additional 50 mg of **3d** (0.23 mmol, 23%) was recovered in a fraction containing biphenyl as an impurity. Around 5% of the *Z* isomer are observed according to $^{1}$H NMR.

**$^{1}$H NMR** (300 MHz, MeOD) δ 7.92 – 7.82 (m, 2H), 7.61 (ddd, *J* = 8.0, 1.7, 0.4 Hz, 1H), 7.57 – 7.39 (m, 4H), 7.21 – 7.16 (m, 1H), 7.00 (ddd, *J* = 8.0, 7.3, 1.2 Hz, 1H), 3.98 (s, 3H).

**{$^{1}$H}$^{13}$C NMR** (75 MHz, MeOD) δ 157.1, 153.1, 142.0, 132.5, 130.6, 128.8, 122.5, 120.4, 116.3, 112.9, 55.4.

The NMR data are consistent with those reported for the known compound (CAS 6319-21-7).

**(*E*)-2-(Phenyldiazenyl)benzonitrile (3e)**

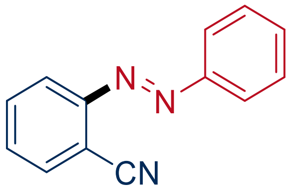


Prepared according to general procedure from phenylhydrazine (108.1 mg, 1 mmol, 1 equiv) and 2-bromobenzonitrile (182.0 mg, 1 mmol, 1 equiv). After purification, 167 mg of **3e** are obtained (0.81 mmol, 81%).

**$^1$H NMR** (400 MHz, MeOD) δ 7.97 – 7.91 (m, 2H), 7.86 (dddd, *J* = 15.6, 8.2, 1.3, 0.6 Hz, 2H), 7.73 (ddd, *J* = 8.2, 7.4, 1.4 Hz, 1H), 7.60 (td, *J* = 7.5, 1.2 Hz, 1H), 7.57 – 7.48 (m, 3H).

**{$^1$H}$^{13}$C NMR** (101 MHz, MeOD) δ 154.3, 153.6, 134.9, 134.9, 133.7, 132.5, 130.4, 124.5, 118.1, 117.6, 113.8.

The NMR data are consistent with those reported for the known compound (CAS 38302-59-9).

**(*E*)-1-(2-Fluorophenyl)-2-phenyldiazene (3f)**

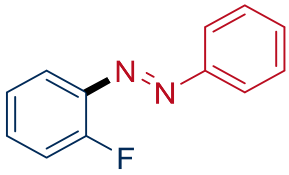


Prepared according to general procedure form phenylhydrazine (108.1 mg, 1 mmol, 1 equiv) and 1-bromo-2-fluorobenzene (175.0 mg, 1 mmol, 1 equiv). After purification, 173 mg of **3f** was obtained (0.86 mmol, 86%). Traces of 2-fluoro-*N*-phenylaniline were still detected, as confirmed by GC/MS and NMR analysis. Around 10% of the *Z* isomer are observed according to $^1$H NMR.

**$^1$H NMR** (400 MHz, MeOD) δ 7.91 (dd, *J* = 7.1, 1.6 Hz, 2H), 7.74 (t, *J* = 7.9 Hz, 1H), 7.58 – 7.45 (m, 4H), 7.31 (dd, *J* = 10.5, 9.0 Hz, 1H), 7.24 (t, *J* = 8.1 Hz, 1H).

**{$^1$H}$^{13}$C NMR** (101 MHz, MeOD) δ 161.4 (d, *J* =256 Hz), 154.1, 141.8 (d, *J* = 7.9 Hz), 134.0 (d, *J* = 8.1 Hz), 132.7, 130.3, 125.6 (d, J = 4.0Hz), 124.0, 118.6, 118.1 (d, *J* = 20.1 Hz).

**{$^1$H}$^{19}$F NMR** (376 MHz, MeOD) δ -126.5.

The NMR data are consistent with those reported for the known compound (CAS 68196-71-4).

**(*E*)-1-(2-Chlorophenyl)-2-phenyldiazene (3g)**

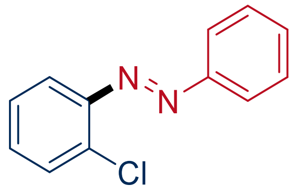


Prepared according to general procedure form phenyl hydrazine (108.1 mg, 1 mmol, 1 equiv) and 1-bromo-2-chlorobenzene (191.4 mg, 1 mmol, 1 equiv). After purification, 144 mg of **3g** are obtained (0.66 mmol, 66%). Around 13% of the *Z* isomer are observed according to $^1$H NMR.

**$^1$H NMR** (400 MHz, MeOD) δ 7.93 – 7.83 (m, 2H), 7.60 (dd, *J* = 7.9, 1.7 Hz, 1H), 7.51 (dd, *J* = 7.9, 1.4 Hz, 1H), 7.49 – 7.41 (m, 3H), 7.38 – 7.32 (m, 1H), 7.28 (ddd, *J* = 7.9, 7.3, 1.4 Hz, 1H).

**{$^1$H}$^{13}$C NMR** (101 MHz, MeOD) δ 153.9, 149.7, 136.3, 133.0, 132.7, 131.7, 130.2, 128.5, 124.2, 118.4.

The NMR data are consistent with those reported for the known compound (CAS 18264-99-8).

**(*E*)-Methyl-4-(phenyldiazenyl)benzoate (3h)**

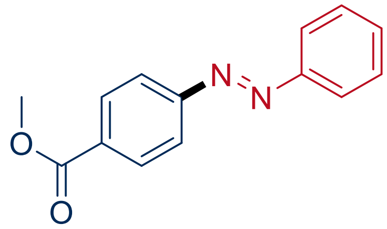


Prepared according to general procedure from phenylhydrazine (108.1 mg, 1 mmol, 1 equiv) and methyl 4-bromobenzoate (215.0 mg, 1 mmol, 1 equiv). After purification, 177 mg of **3h** are obtained (0.74 mmol 74%).

According to $^1$H NMR analysis, this compound was isolated and observed in solution as a mixture of *Z*/*E* isomers in a 25:75 ratio.

**$^1$H NMR** (400 MHz, MeOD) δ 8.24 – 8.16 (m, 2.1H), 8.02 – 7.88 (m, 4.9H), 7.62 – 7.51 (m, 3H), 7.33 – 7.15 (m, 1.4H), 6.96 – 6.80 (m, 1.7H), 3.95 (s, 3H), 3.87 (s, 1.3H).

**{$^1$H}$^{13}$C NMR** (101 MHz, MeOD) δ 133.1, 131.7, 131.4, 130.4, 130.00, 124.1, 123.7, 121.7, 121.1, 52.9.

The NMR data are consistent with those reported for the known compound (CAS 2918-88-9).

**N,N-Dimethyl-4-(phenyldiazenyl)benzamide (3i)**

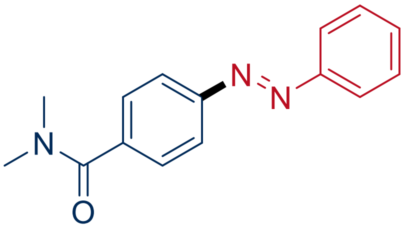

Prepared according to general procedure from phenylhydrazine (108.1 mg, 1 mmol, 1 equiv) and 4-bromo-*N*,*N*-dimethylbenzamide (228.1 mg, 1 mmol, 1 equiv). After purification, 184 mg of **3i** are obtained (0.73 mmol, 73%).

**$^1$H NMR** (400 MHz, MeOD) δ 8.02 – 7.96 (m, 2H), 7.96 – 7.91 (m, 2H), 7.65 – 7.59 (m, 2H), 7.59 – 7.50 (m, 3H), 3.09 (d, *J* = 37.6 Hz, 6H).

**{$^1$H}$^{13}$C NMR** (101 MHz, MeOD) δ 172.9, 154.4, 153.9, 139.7, 132.8, 130.4, 129.1, 124.0, 123.9, 40.00, 35.67.

**HRMS** (EI) theoretical mass for [M+H]: 254.1288; found: 254.1295.

**(*E*)-1-(4-Nitrophenyl)-2-phenyldiazene (3j)**

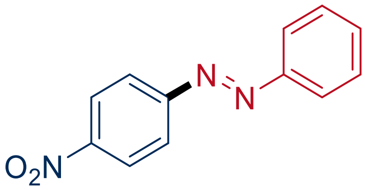

Prepared according to general procedure from phenylhydrazine (108.1 mg, 1 mmol, 1 equiv) and 1-bromo-4-nitrobenzene (202.0 mg, 1 mmol, 1 equiv). After purification, 79 mg of **3j** are obtained (0.35 mmol, 35%).

**$^1$H NMR** (400 MHz, MeOD) δ 8.47 – 8.40 (m, 2H), 8.13 – 8.05 (m, 2H), 8.03 – 7.95 (m, 2H), 7.63 – 7.56 (m, 3H).

**{$^1$H}$^{13}$C NMR** (101 MHz, MeOD) δ 157.2, 153.9, 150.3, 133.6, 130.5, 125.9, 124.5, 124.4.

The NMR data are consistent with those reported for the known compound (CAS 2491-52-3).

**(*E*)-*N*,*N*-Dimethyl-4-(phenyldiazenyl)aniline (3k)**

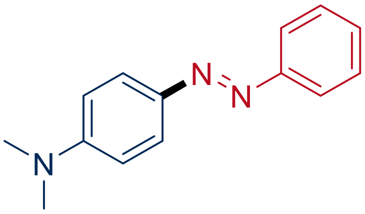

Prepared according to general procedure from phenylhydrazine (108.1 mg, 1 mmol, 1 equiv) and 4-bromo-*N′*,*N′*-dimethylaniline (200.1 mg, 1 mmol, 1 equiv). After purification, 124 mg of **3k** are obtained (0.55 mmol, 55%).

**$^1$H NMR** (400 MHz, MeOD) δ 7.85 – 7.80 (m, 2H), 7.80 – 7.76 (m, 2H), 7.52 – 7.43 (m, 2H), 7.43 – 7.35 (m, 1H), 6.86 – 6.79 (m, 2H), 3.09 (s, 6H).

**{$^1$H}$^{13}$C NMR** (101 MHz, MeOD) δ 154.5, 154.3, 144.8, 130.5, 130.1, 125.9, 123.1, 112.7, 40.4.

The NMR data are consistent with those reported for the known compound (CAS 60-11-7).

**(*E*)-1-(4-(Methylthio)phenyl)-2-phenyldiazene (3l)**

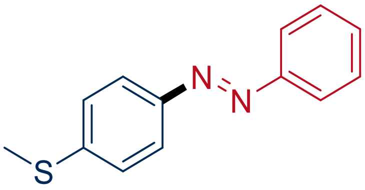

Prepared according to general procedure from phenylhydrazine (108.1 mg, 1 mmol, 1 equiv) and 4-bromothioanisole (203.1 mg, 1 mmol, 1 equiv). After purification 161 mg of **3l** are obtained (0.71 mmol, 71%).

**$^1$H NMR** (400 MHz, MeOD) δ 7.92 – 7.82 (m, 4H), 7.56 – 7.46 (m, 3H), 7.43 – 7.36 (m, 2H), 2.56 (s, 3H).

**{$^1$H}$^{13}$C NMR** (101 MHz, MeOD) δ 154.1, 151.2, 145.1, 132.0, 130.2, 127.0, 124.3, 123.7, 15.1.

**HRMS** (EI) theoretical mass for [M+H]: 229.0794; found: 229.0799.

**(*E*)-Azobenzene (3m)**

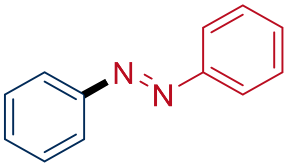


Prepared according to general procedure from phenylhydrazine (108.1 mg, 1 mmol, 1 equiv) and bromobenzene (157.0 mg, 1 mmol, 1 equiv) After purification, 78 mg of **3m** are obtained (0.43 mmol, 43%). Around 5% of the *Z* isomer are observed according to $^{1}$H NMR.

**$^{1}$H NMR** (300 MHz, MeOD) δ 7.95 – 7.84 (m, 4H), 7.59 – 7.42 (m, 6H).

**{$^{1}$H}$^{13}$C NMR** (75 MHz, MeOD) δ 153.9, 132.2, 130.2, 123.8.

The NMR data are consistent with those reported for the known compound (CAS 103-33-3).

**(*E*)-1-(3(*tert*-Butyl)phenyl)-2-phenyldiazene (3n)**

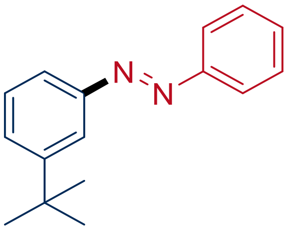


Prepared according to general procedure from phenylhydrazine (108.1 mg, 1 mmol, 1 equiv) and 1-bromo-3-*tert*-butylbenzene (213.1 mg, 1 mmol, 1 equiv). After purification, 201 mg of **3n** are obtained (0.84 mmol, 84%).

**$^{1}$H NMR** (400 MHz, MeOD) δ 7.94 (t, *J* = 2.0 Hz, 1H), 7.89 – 7.83 (m, 2H), 7.66 (ddd, *J* = 7.7, 1.9, 1.1 Hz, 1H), 7.48 – 7.31 (m, 5H), 1.29 (s, 9H).

**{$^{1}$H}$^{13}$C NMR** (101 MHz, MeOD) δ 153.8, 153.7, 153.4, 132.0, 130.1, 129.8, 129.2, 123.7, 121.3, 120.6, 35.6, 31.7.

**HRMS** (EI) theoretical mass for [M+H]: 239.1543; found: 239.1547.

**(*E*)-1-(3,5-Dimethylphenyl)-2-phenyldiazene (3o)**

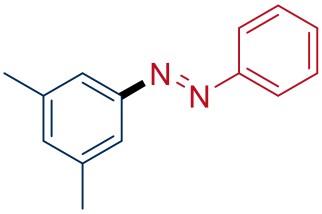


Prepared according to general procedure from phenylhydrazine (108.1 mg, 1 mmol, 1 equiv) and 3,5-dimethylbromobenzene (185.1 mg, 1 mmol, 1 equiv). After purification, 163 mg of **3o** are obtained (0.78 mmol, 78%). Around 10% of the *Z* isomer are observed according to $^1$H NMR.

**$^1$H NMR** (300 MHz, MeOD) δ 7.87 – 7.77 (m, 2H), 7.50 – 7.33 (m, 5H), 7.03 – 6.95 (m, 1H), 2.29 – 2.26 (m, 6H).

**{$^1$H}$^{13}$C NMR** (75 MHz, MeOD) δ 153.9, 153.9, 139.8, 133.6, 131.9, 130.1, 123.1, 121.6, 21.4.

The NMR data are consistent with those reported for the known compound (CAS 77775-95-2).

**(*E*)-3-(Phenyldiazenyl)pyridine (3p)**

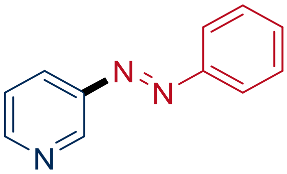


Prepared according to general procedure from phenylhydrazine (108.1 mg, 1 mmol, 1 equiv) and 2-bromopyridine (158.0 mg, 1 mmol, 1 equiv). After purification, 129 mg of **3p** are obtained (0.70 mmol, 70%).

**$^1$H NMR** (400 MHz, MeOD) δ 8.96 (dd, *J* = 2.4, 0.8 Hz, 1H), 8.56 (dd, *J* = 4.8, 1.6 Hz, 1H), 8.08 (ddd, *J* = 8.2, 2.4, 1.5 Hz, 1H), 7.82 (tddd, *J* = 5.9, 4.2, 2.6, 1.4 Hz, 2H), 7.51 – 7.41 (m, 4H).

**{$^1$H}$^{13}$C NMR** (101 MHz, MeOD) δ 153.5, 152.1, 149.2, 147.1, 133.1, 130.3, 128.9, 125.7, 124.1.

The NMR data are consistent with those reported for the known compound (CAS 2569-55-3).

**(*E*)-1,4-Bis(phenyldiazenyl)benzene (1161-45-1) (3q)**

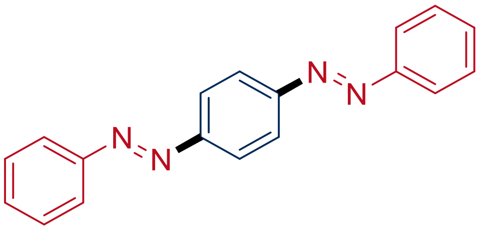


Prepared according to a modified procedure from phenylhydrazine (108.1 mg, 1 mmol, 2 equiv) and 1,4-dibromobenzene (167.9 mg, 0.5 mmol, 1 equiv). 95 mg of **3q** are obtained (0.33 mmol, 66%).

Ethyl acetate present in the NMR spectrums.

**$^{1}$H NMR** (300 MHz, $CDCl_3$) δ 7.93 (s, 4H), 7.86 – 7.79 (m, 4H), 7.45 – 7.32 (m, 6H).

**{$^{1}$H}$^{13}$C NMR** (75 MHz, $CDCl_3$) δ 153.6, 152.6, 131.3, 129.0, 123.6, 122.9.

The NMR data are consistent with those reported for the known compound (CAS 1161-45-1).

**(*E*)-1-(*o*-Tolyl)-2-(4-trifluoromethyl)phenyl)diazene (4a)**

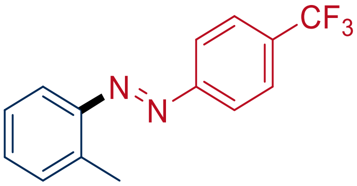


Prepared according to general procedure from 4-trifluoromethylphenylhydrazine (176.1 mg, 1 mmol, 1 equiv) and 2-bromotoluene (171.0 mg, 1 mmol, 1 equiv). After purification, 207 mg of **4a** are obtained (0.78 mmol, 78%).

**$^{1}$H NMR** (300 MHz, MeOD) δ 7.87 – 7.76 (m, 2H), 7.70 – 7.59 (m, 2H), 7.50 (dd, *J* = 8.0, 1.3 Hz, 1H), 7.33 – 7.19 (m, 2H), 7.13 (dddd, *J* = 8.1, 7.0, 1.8, 0.5 Hz, 1H), 2.57 (s, 3H).

**{$^{1}$H, $^{19}$F}$^{13}$C NMR** (75 MHz, MeOD) δ 155.9, 151.4, 140.1, 133.0, 132.4, 127.4, 127.2 (q, *J* = 3.7 Hz), 124.1, 116.2, 17.6.

**{$^{1}$H}$^{19}$F NMR** (282 MHz, MeOD) δ -63.8.

**HRMS** (EI) theoretical mass for [M]: 264.08688; found: 264.08649.

**(*E*)-1-(4-Chlorophenyl)-2-(*o*-tolyl)diazene (4b)**

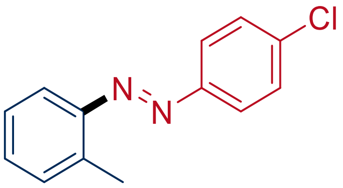


Prepared according to general procedure from (4-chlorophenyl)hydrazine (142.6 mg, 1 mmol, 1 equiv) and 2-bromotoluene (171.0 mg, 1 mmol, 1 equiv). After purification, 80 mg of **4b** are obtained (0.37 mmol, 37%).

**$^1$H NMR** (400 MHz, MeOD) δ 7.90 – 7.82 (m, 2H), 7.60 (dd, *J* = 7.8, 1.0 Hz, 1H), 7.55 – 7.49 (m, 2H), 7.44 – 7.32 (m, 2H), 7.29 – 7.21 (m, 1H), 2.69 (s, 3H).

**{$^1$H}$^{13}$C NMR** (101 MHz, MeOD) δ 152.8, 151.7, 139.6 137.8, 132.5, 132.4, 130.4, 127.5, 125.3, 116.2, 17.6.

The NMR data are consistent with those reported for the known compound (CAS 1992832-16-2).

**(*E*)-1,2-Di-*o*-tolyldiazene (4c)**

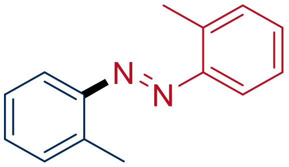


Prepared according to general procedure from (2-methylphenyl)hydrazine (122.2 mg, 1 mmol, 1 equiv) and 2-bromotoluene (171.0 mg, 1 mmol, 1 equiv). After purification, 132 mg of **4c** are obtained (0.63 mmol, 63%).

**$^1$H NMR** (400 MHz, MeOD) $^1$H NMR (400 MHz, MeOD) δ 7.58 (d, *J* = 7.7 Hz, 2H), 7.40 – 7.31 (m, 4H), 7.30 – 7.20 (m, 2H), 2.71 (s, 6H).

**{$^1$H}$^{13}$C NMR** (101 MHz, MeOD) δ 152.3, 139.1, 132.4, 132.0, 127.5, 116.6, 17.7.

The NMR data are consistent with those reported for the known compound (CAS 584-90-7).

**(*E*)-1-(2-Fluorophenyl)-2-(*o*-tolyl)diazene (4d)**

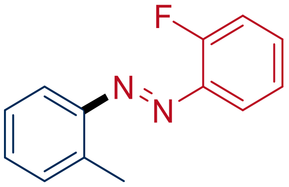


Prepared according to general procedure from (2-fluorophenyl)hydrazine (126.1 mg, 1 mmol, 1 equiv) and 2-bromotoluene (171.0 mg, 1 mmol, 1 equiv). After purification of **4d** are obtained, 110 mg (0.55 mmol, 55%).

**$^1$H NMR** (400 MHz, MeOD) δ 7.71 (td, *J* = 7.8, 1.7 Hz, 1H), 7.61 (dd, *J* = 7.9, 1.2 Hz, 1H), 7.50 (dddd, *J* = 8.3, 7.3, 5.0, 1.8 Hz, 1H), 7.43 – 7.20 (m, 5H), 2.70 (d, *J* = 1.1 Hz, 3H).

**{$^1$H}$^{13}$C NMR** (101 MHz, MeOD) δ 161.4 (d, *J* = 256.6 Hz), 160.1, 152.1, 142.2 (d, *J* = 6.9 Hz), 139.8, 133.7 (d, *J* = 8.4 Hz), 132.6 (d, *J* = 25.6 Hz), 127.5, 125.6 (d, *J* = 3.9 Hz), 118.9, 118.0 (d, *J* = 19.9 Hz), 116.5, 17.6.

**{$^1$H}$^{19}$F NMR** (376 MHz, MeOD) δ -126.8 – -126.9 (m).

The NMR data are consistent with those reported for the known compound (CAS 2479972-17-1).

**(*E*)-1-(2-Chloro-5-(trifluoromethyl)phenyl)-2-(*o*-tolyl)diazene (4e)**

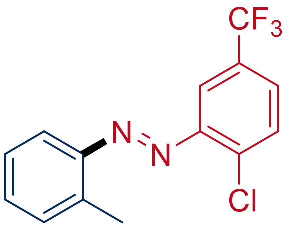


Prepared according to general procedure from [2-chloro-5-(trifluoromethyl)phenyl]hydrazine (210.6 mg, 1 mmol, 1 equiv) and 2-bromotoluene (171.0 mg, 1 mmol, 1 equiv). After purification, 96 mg of **4e** (0.32 mmol, 32%).

**$^1$H NMR** (300 MHz, MeOD) δ 7.88 (dt, *J* = 2.1, 0.7 Hz, 1H), 7.85 – 7.73 (m, 2H), 7.69 (ddd, *J* = 8.1, 0.9, 0.5 Hz, 1H), 7.51 – 7.35 (m, 2H), 7.35 – 7.23 (m, 1H), 2.74 (s, 3H).

**{$^1$H, $^{19}$F}$^{13}$C NMR** (75 MHz, MeOD) δ 151.9, 150.3, 140.5, 139.7, 133.7, 133.0, 132.7, 128.9, 128.9, 127.7, 117.2, 115.7, 115.6, 17.7.

**{$^1$H}$^{19}$F NMR** (282 MHz, MeOD) δ -64.3.

**HRMS** (EI) theoretical mass for [M]: 298.04791; found: 298.04882.

**(*E*)-1-(2,6-Difluorophenyl)-2-(2,6-dimethoxyphenyldiazene) (5a)**

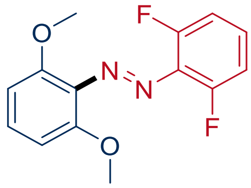


Prepared according to general procedure from (2,6-difluorophenyl)hydrazine (144.1 mg, 1 mmol, 1 equiv) and 2,6-dimethoxybromobenzene (217.1 mg, 1 mmol, 1 equiv). After purification, 174 mg (0.63 mmol, 63%) of **5a** are obtained.

**$^1$H NMR** (300 MHz, DMSO) δ 7.58 – 7.46 (m, 1H), 7.38 (t, *J* = 8.5 Hz, 1H), 7.34 – 7.24 (m, 2H), 6.83 (d, *J* = 8.5 Hz, 2H), 3.78 (s, 6H).

**{$^1$H, $^{19}$F}$^{13}$C NMR** (75 MHz, DMSO) δ 155.9 (d, *J* = 4.6 Hz), 152.5 (d, *J* = 4.7 Hz), 151.9, 133.1, 131.3, 131.1 (t, *J* = 10.3 Hz), 113.2 – 112.7 (m), 105.3, 56.3.

**{$^1$H}$^{19}$F NMR** (282 MHz, DMSO) δ -123.8 (dd, *J* = 9.1, 6.1 Hz).

The NMR data are consistent with those reported for the known compound (CAS 2972643-51-7).

**(*E*)-1-(2,6-Difluorophenyl)-2-(*o*-tolyl)diazene (5b)**

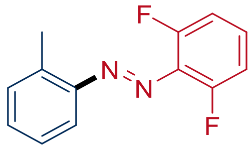


Prepared according to general procedure from (2,6-difluorophenyl)hydrazine (144.1 mg, 1 mmol, 1 equiv) and 2-bromotoluene (171.0 mg, 1 mmol, 1 equiv). After purification, 93 mg of **5b** was obtained (0.40 mmol, 40%). An additional 98 mg of **5b** (0.42 mmol, 42%) was recovered in a second fraction containing approximately 5% biphenyls, which could not be fully separated during purification.

**$^1$H NMR** (300 MHz, MeOD) δ 7.56 (d, *J* = 7.9 Hz, 1H), 7.46 – 7.34 (m, 3H), 7.26 (dddd, *J* = 8.1, 6.6, 2.2, 0.6 Hz, 1H), 7.17 – 7.06 (m, 2H), 2.65 (s, 3H).

**{$^1$H, $^{19}$F}$^{13}$C NMR** (75 MHz, MeOD) δ 158.8 (d, *J* = 4.4 Hz), 155.4 (d, *J* = 4.4 Hz), 152.7, 140.1, 133.2, 132.5, 131.8 (t, *J* = 10.4 Hz), 127.5, 115.7, 113.8 – 113.4 (m), 17.4.

**{$^1$H}$^{19}$F NMR** (282 MHz, MeOD) δ -124.2 (dd, *J* = 9.4, 5.9 Hz).

**(*E*)-1-(2,6-Dimethoxyphenyl)-2-(*o*-tolyl)diazene (5c)**

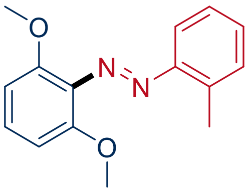


Prepared according to general procedure from 2-methylphenylhydrazine (122.2 mg, 1 mmol, 1 equiv) and 2,6-dimethoxybromonenzene (217.1 mg, 1 mmol, 1 equiv). After purification, 120mg of **5d** are obtained (0.47 mmol, 47%). According to NMR analysis, this compound was isolated and observed in solution as a mixture of *Z*/*E* isomers in a 25:75 ratio. The $^{1}$H NMR peaks correspond exclusively to the *E* isomer, while the $^{13}$C NMR signals include contributions from both the *Z* and *E* isomers.

**$^{1}$H NMR (300 MHz, DMSO)** δ 7.49 – 7.20 (m, 6H), 6.82 (d, *J* = 8.5 Hz, 2H), 3.77 (s, 6H), 2.56 (s, 3H).

**{$^{1}$H}$^{13}$C NMR** (75 MHz, DMSO) δ 151.7, 151.1, 148.1, 136.6, 133.4, 131.2, 130.9, 130.5, 129.5, 128.1, 127.9, 126.5, 125.1, 115.0, 113.8, 105.4, 104.4, 56.3, 55.6, 17.0, 16.6.

**HRMS** (EI) theoretical mass for [M+H]: 257.1290; found: 257.1292.

**(*E*)-1-(2,6-Difluorophenyl)-2-(3,5-dimethylphenyl)diazene (5d)**

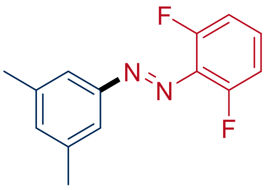


Prepared according to general procedure from (2.6-difluorophenyl)hydrazine (144.1 mg, 1 mmol, 1 equiv) and 3,5-dimethylbromobenzene (185.1 mg, 1 mmol, 1 equiv). After purification, 197 mg of **5c** are obtained (0.80 mmol, 80%) are obtained. According to NMR analysis, this compound was isolated and observed in solution as a mixture of *Z*/*E* isomers in a 20:80 ratio. The $^{1}$H NMR peaks correspond exclusively to the *E* isomer, while the $^{13}$C NMR signals include contributions from both the *Z* and *E* isomers.

**$^{1}$H NMR** (300 MHz, DMSO) δ 7.61 – 7.51 (m, 1H), 7.49 (dq, *J* = 1.9, 0.6 Hz, 2H), 7.39 – 7.23 (m, 3H), 2.38 (q, *J* = 0.7 Hz, 6H).

**{$^{1}$H}$^{13}$C NMR** (75 MHz, DMSO) δ 156.5 (d, *J* = 4.1 Hz), 153.1 (d, *J* = 4.5 Hz), 152.7, 138.9, 138.4, 137.9, 133.9, 131.4 (t, *J* = 10.6 Hz), 130.4, 120.3, 115.8, 114.7, 113.0 (dd, *J* = 20.9, 2.8 Hz), 20.7.

**{$^{1}$H}$^{19}$F NMR** (282 MHz, DMSO) δ -122.7 (dd, *J* = 9.7, 6.1 Hz).

**HRMS** (EI) theoretical mass for [M+H]: 247.1047; found: 247.1049.

# IV. $^{1}$H, $^{13}$C and $^{19}$F NMR spectra:

**$^{1}$H, $^{13}$C NMR of 3a:**

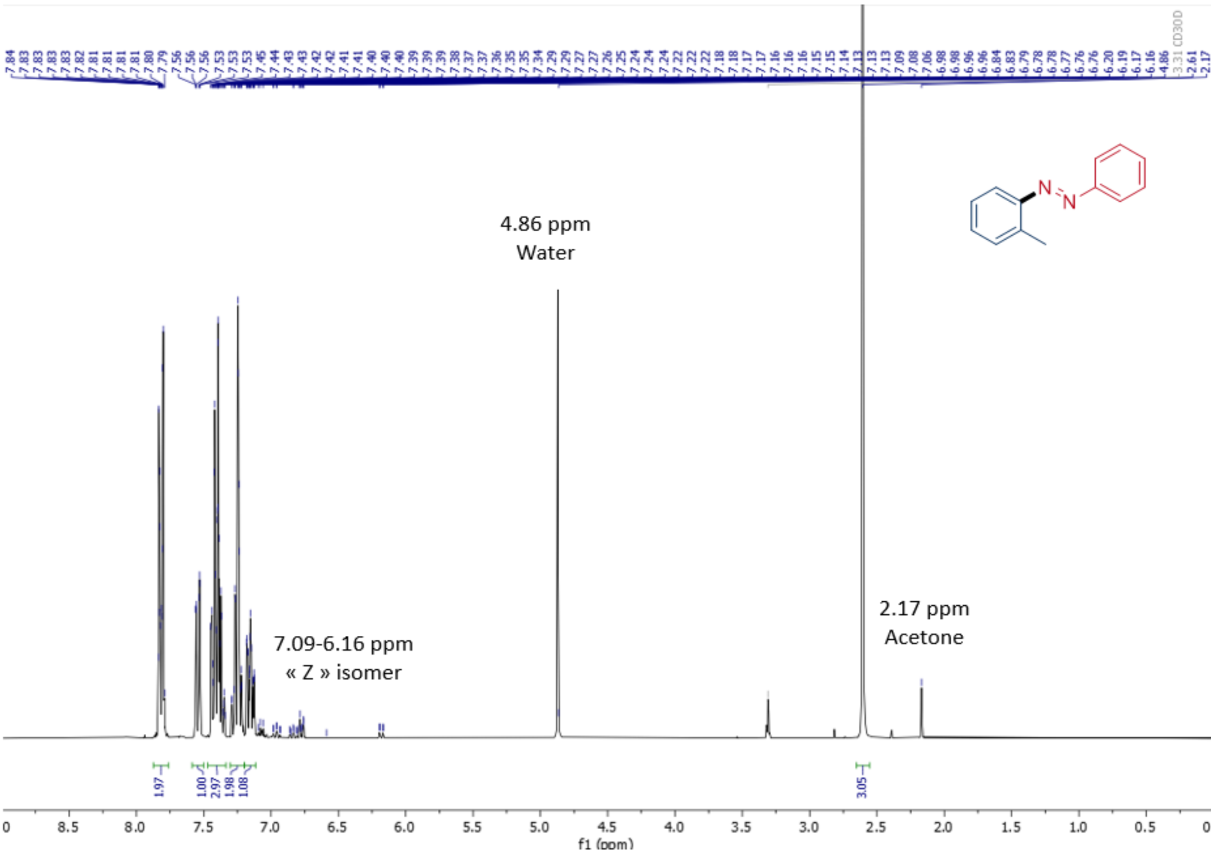


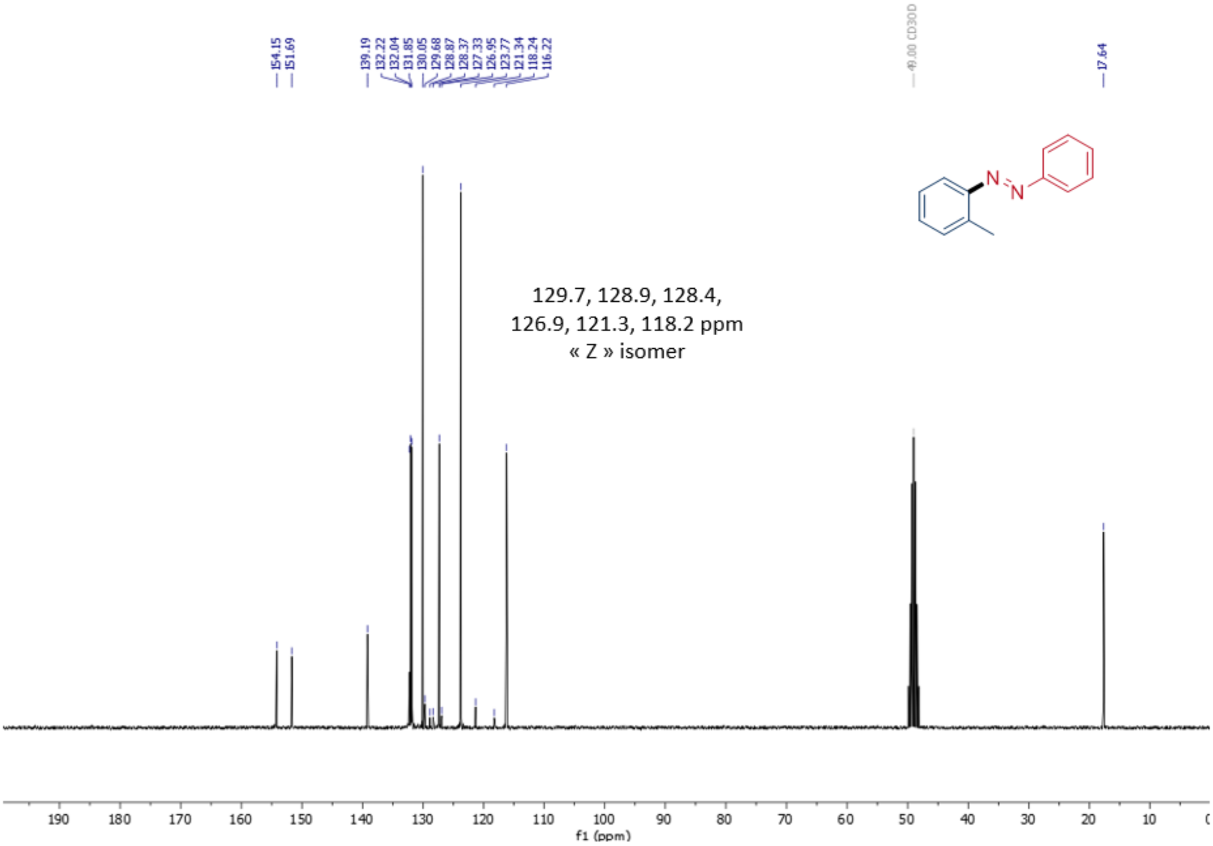

$^{1}H$, $^{13}C$ NMR of **3b**:

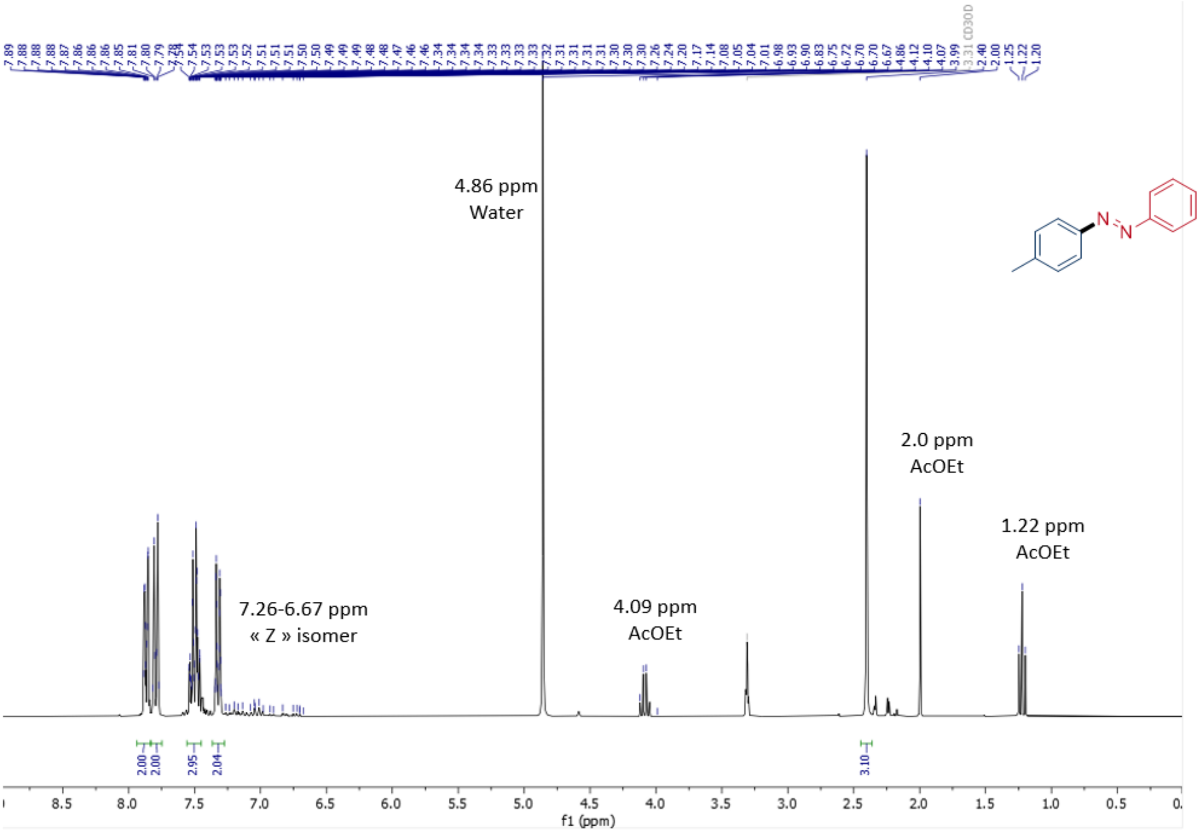


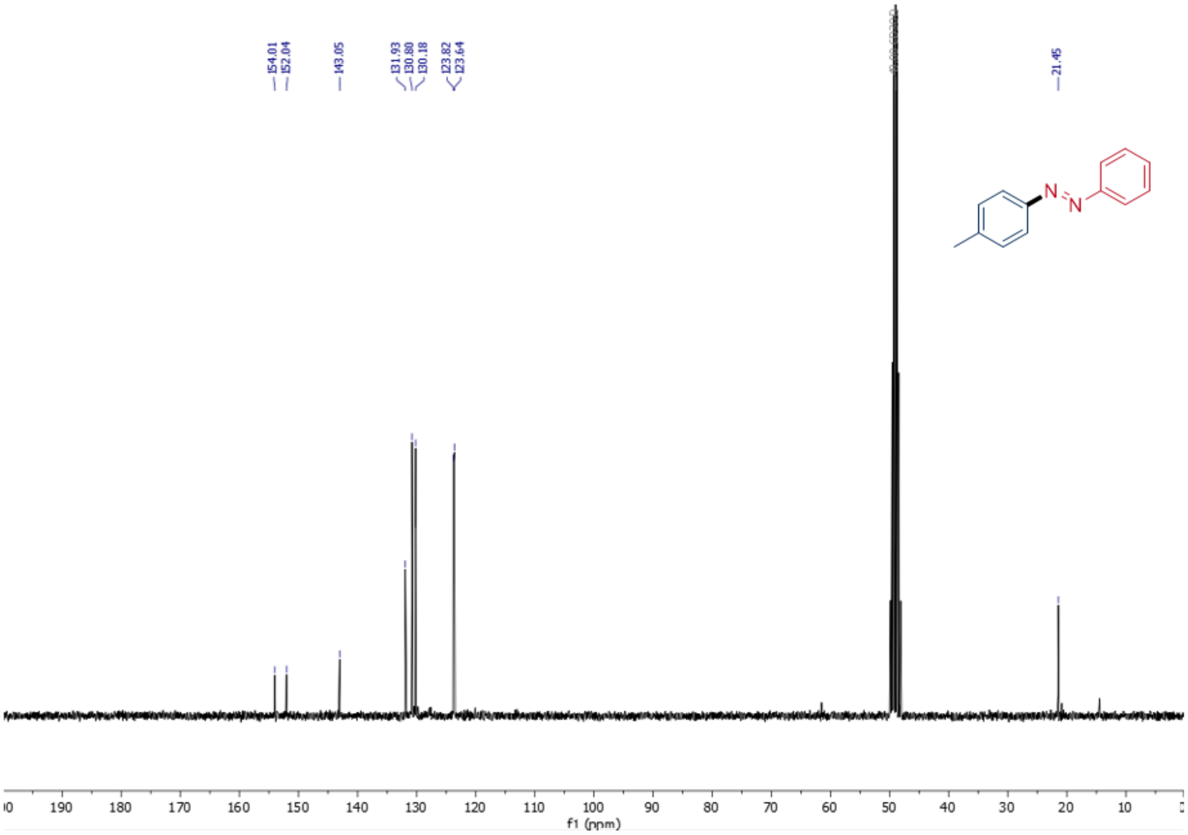

**$^{1}H$, $^{13}C$, $^{19}F$ NMR of 3c:**

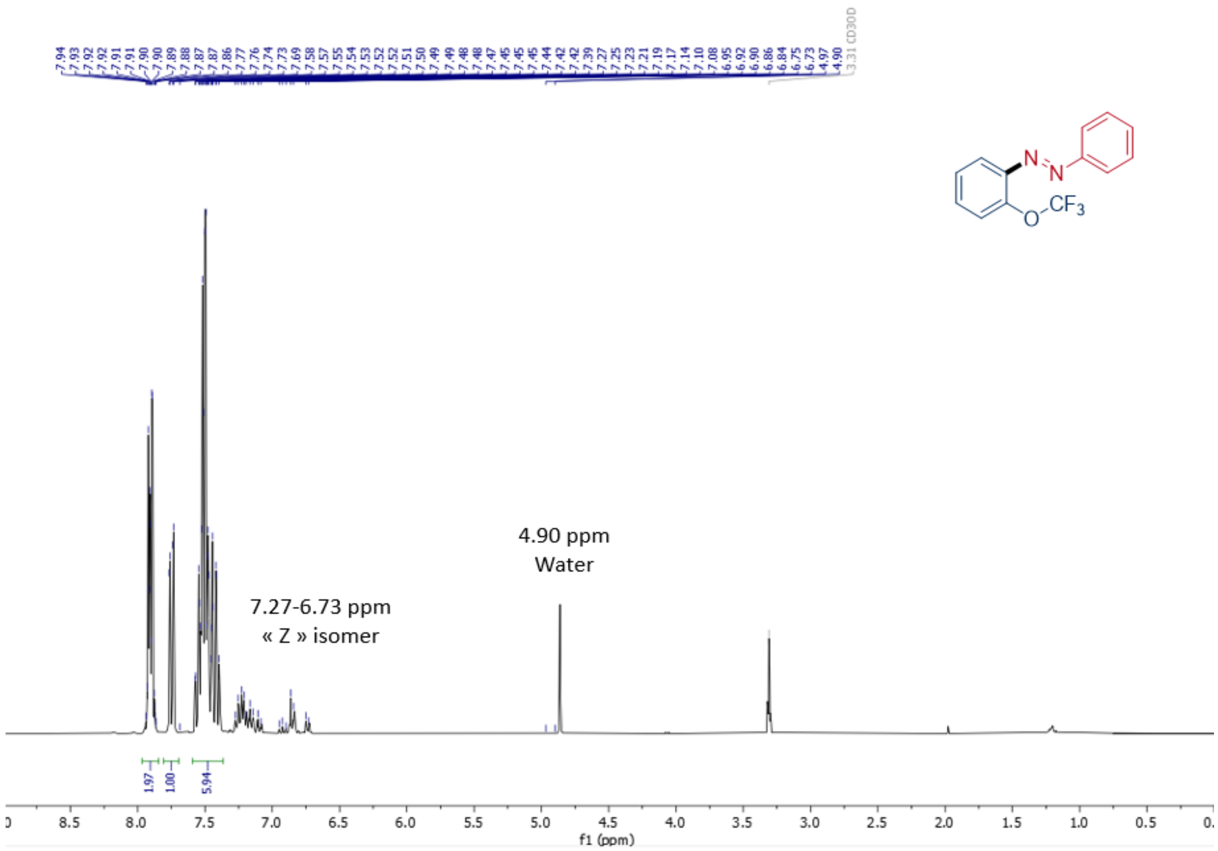


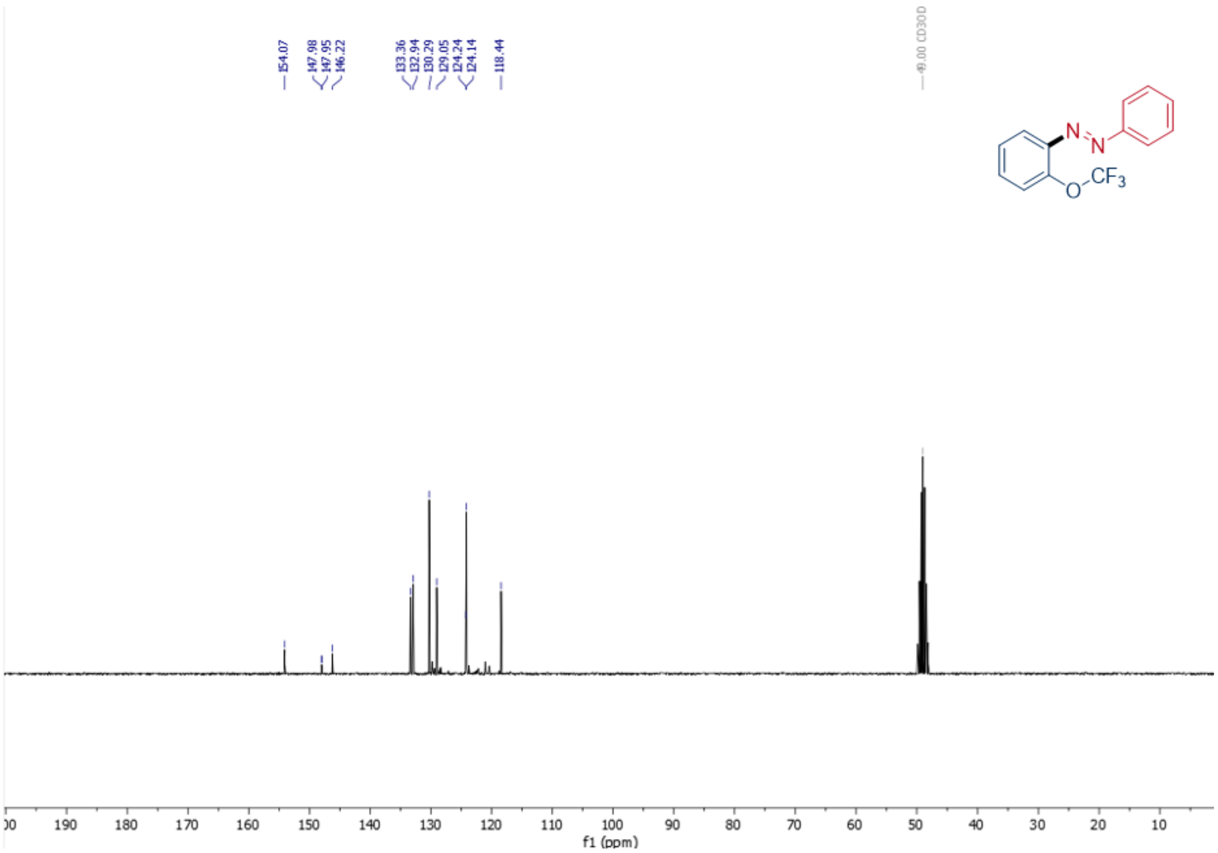

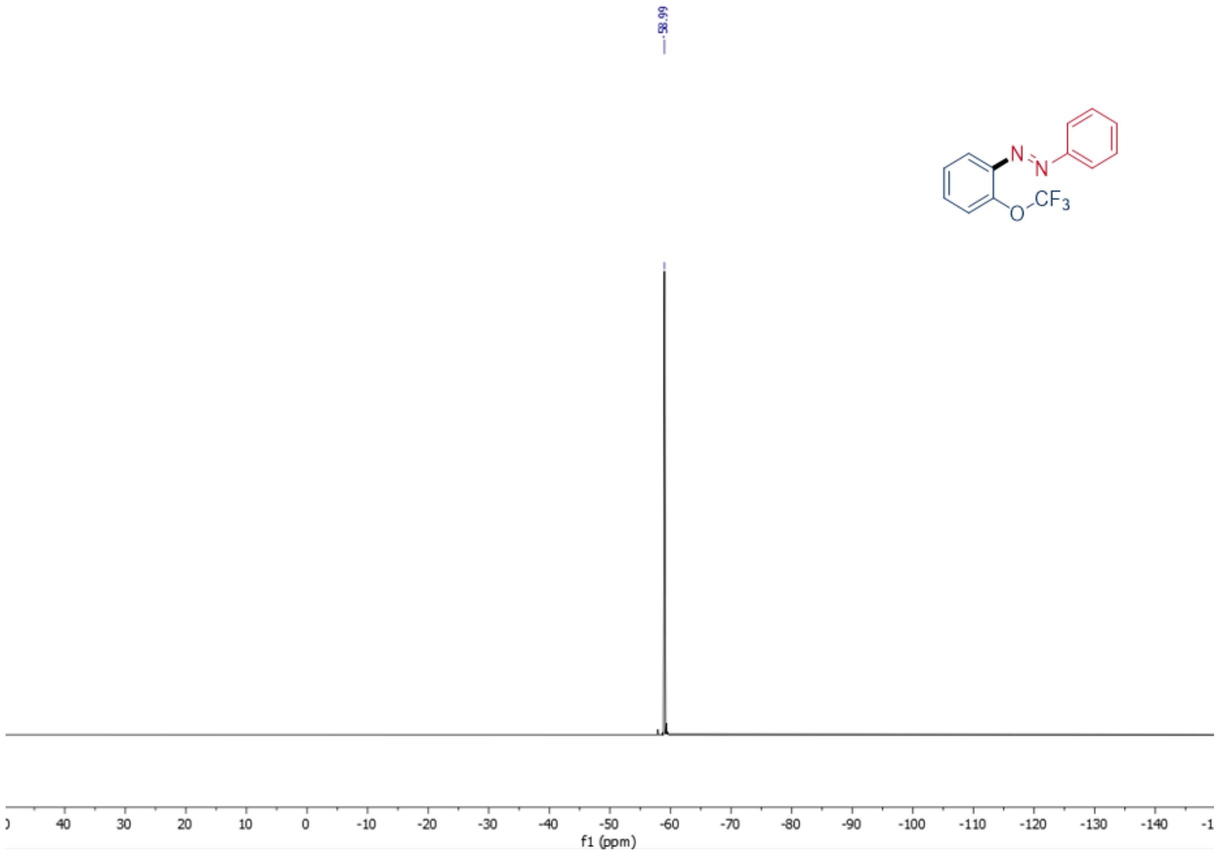

-58.99
N
N
O
CF3
40
30
20
10
0
-10
-20
-30
-40
-50
-60
-70
-80
-90
-100
-110
-120
-130
-140
f1 (ppm)

**$^{1}H$, $^{13}C$ NMR of 3d:**

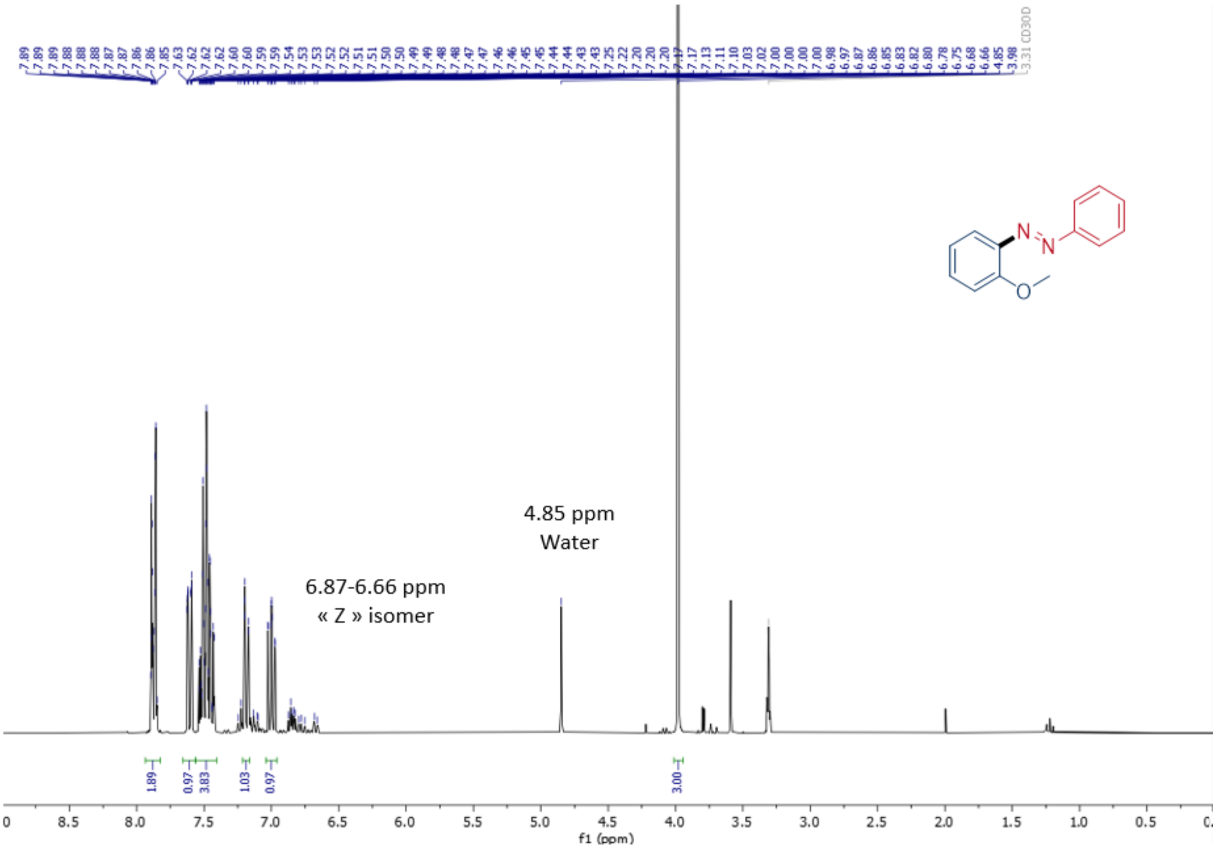


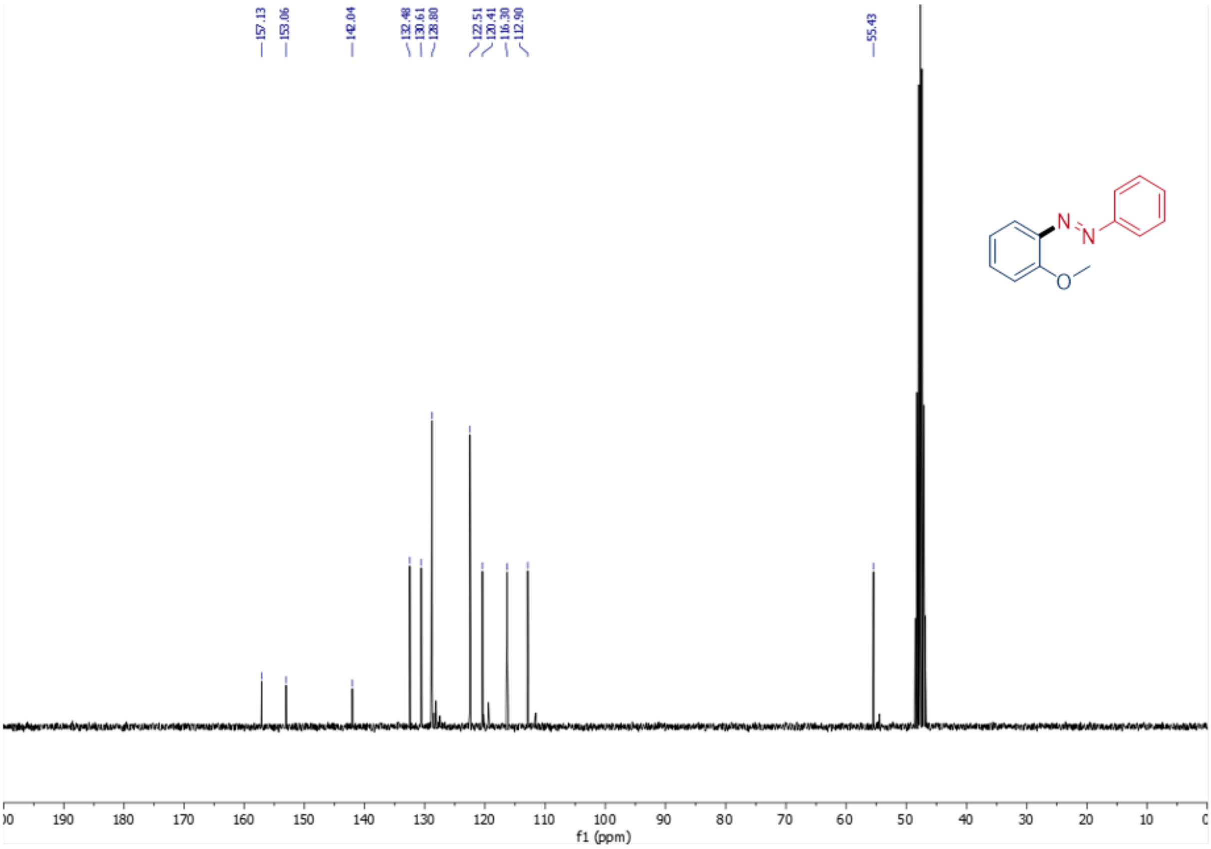

$^{1}$H, $^{13}$C NMR of 3e:

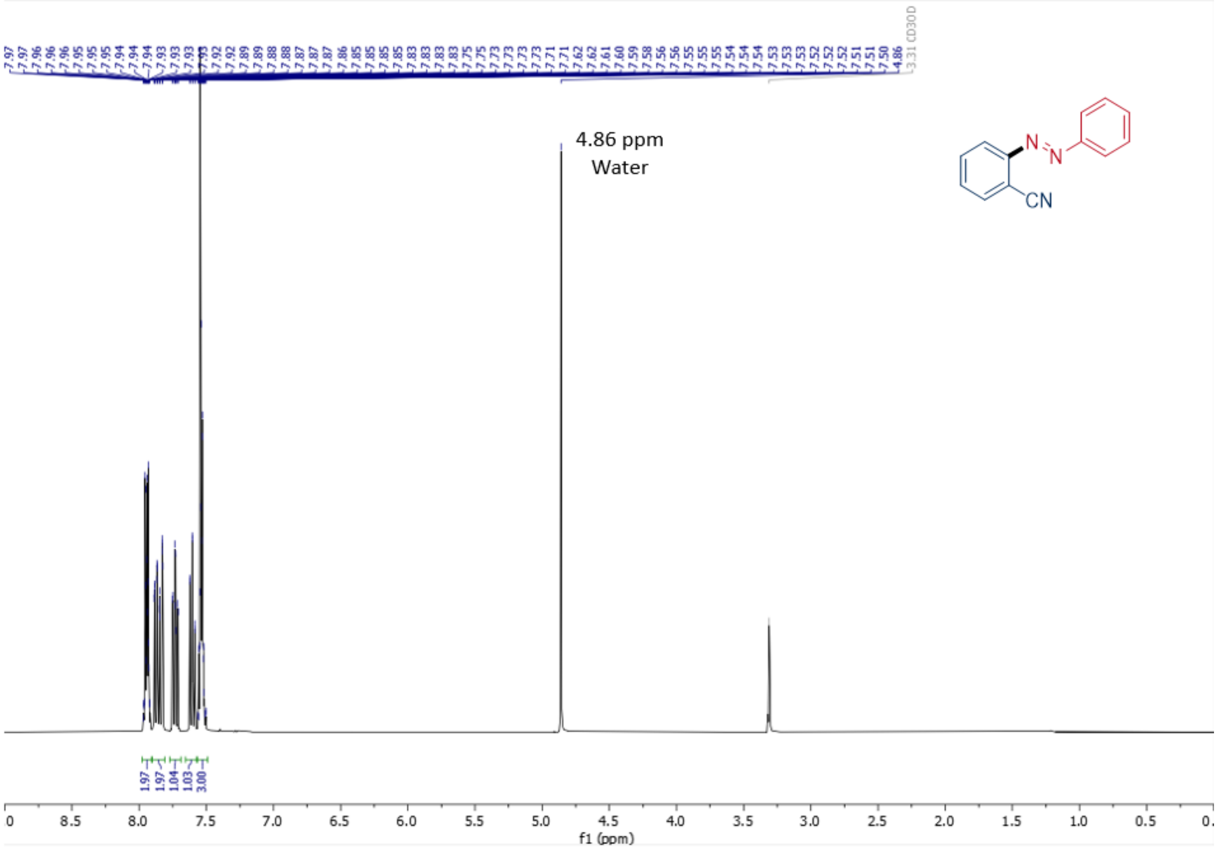


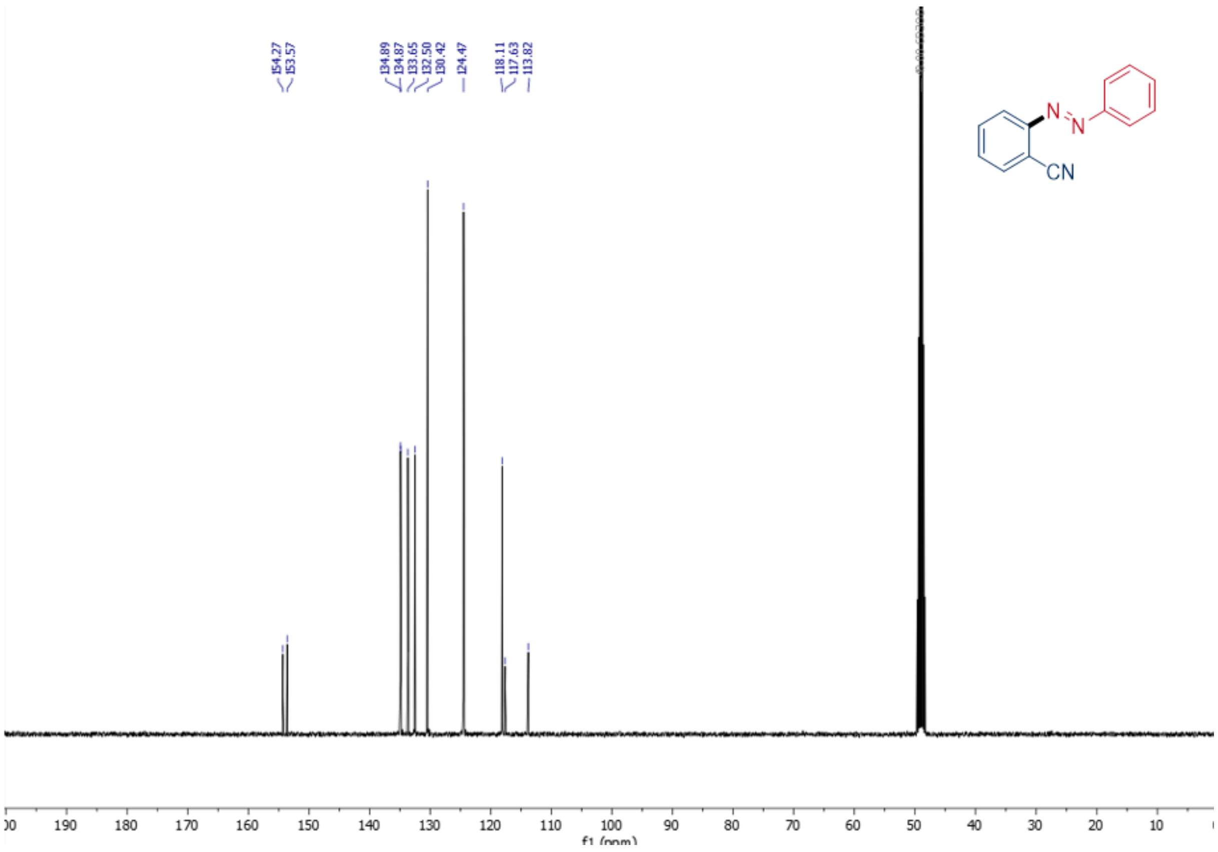

**$^{1}H$, $^{13}C$, $^{19}F$ NMR of 3f:**

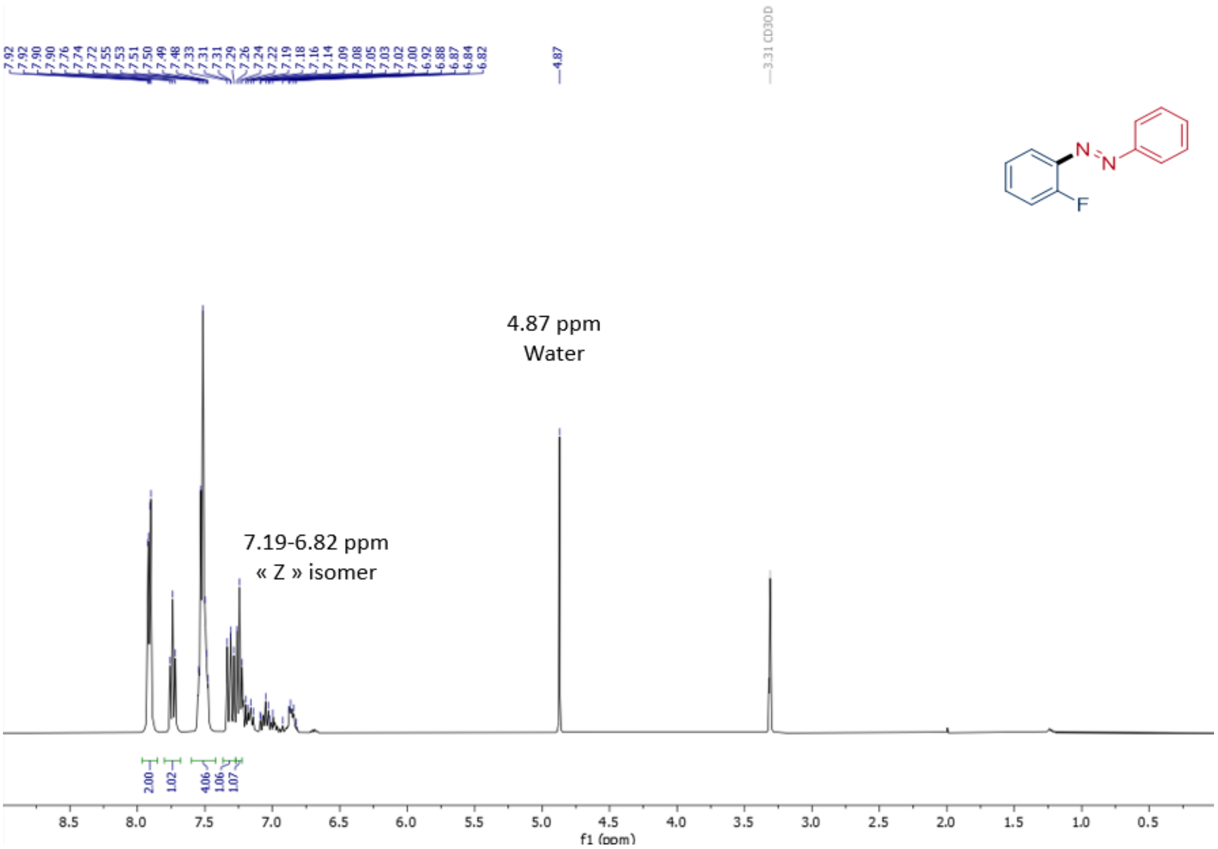


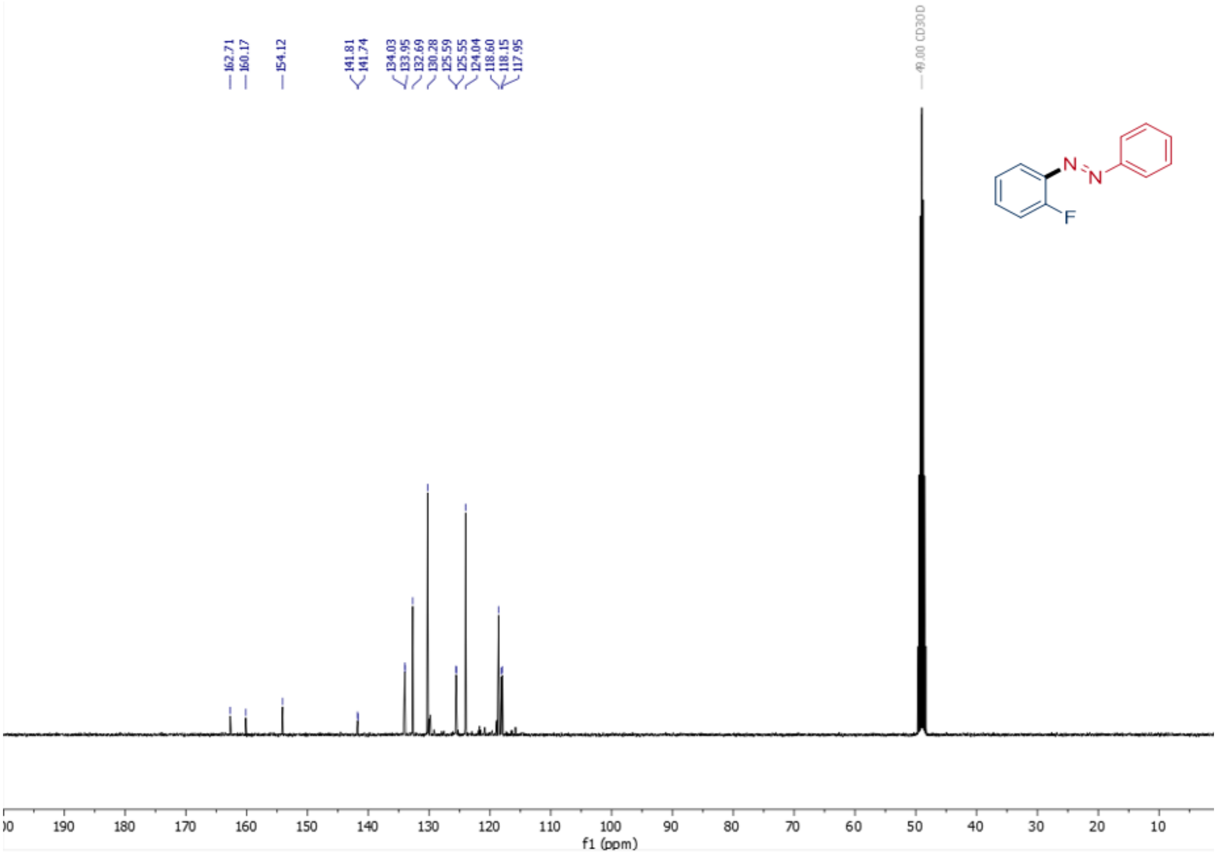

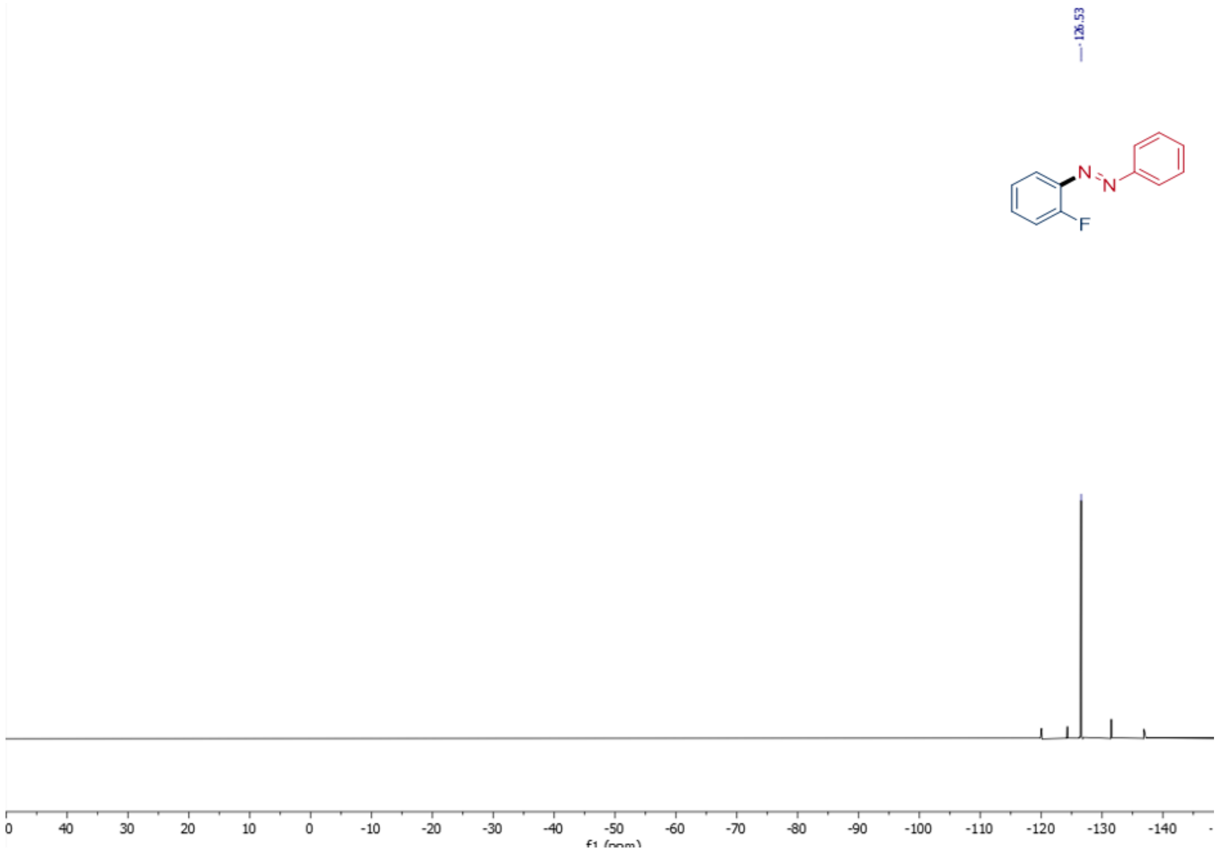

126.53
N
N
F
0
40
30
20
10
0
-10
-20
-30
-40
-50
-60
-70
-80
-90
-100
-110
-120
-130
-140

**$^1$H, $^{13}$C NMR of 3g:**

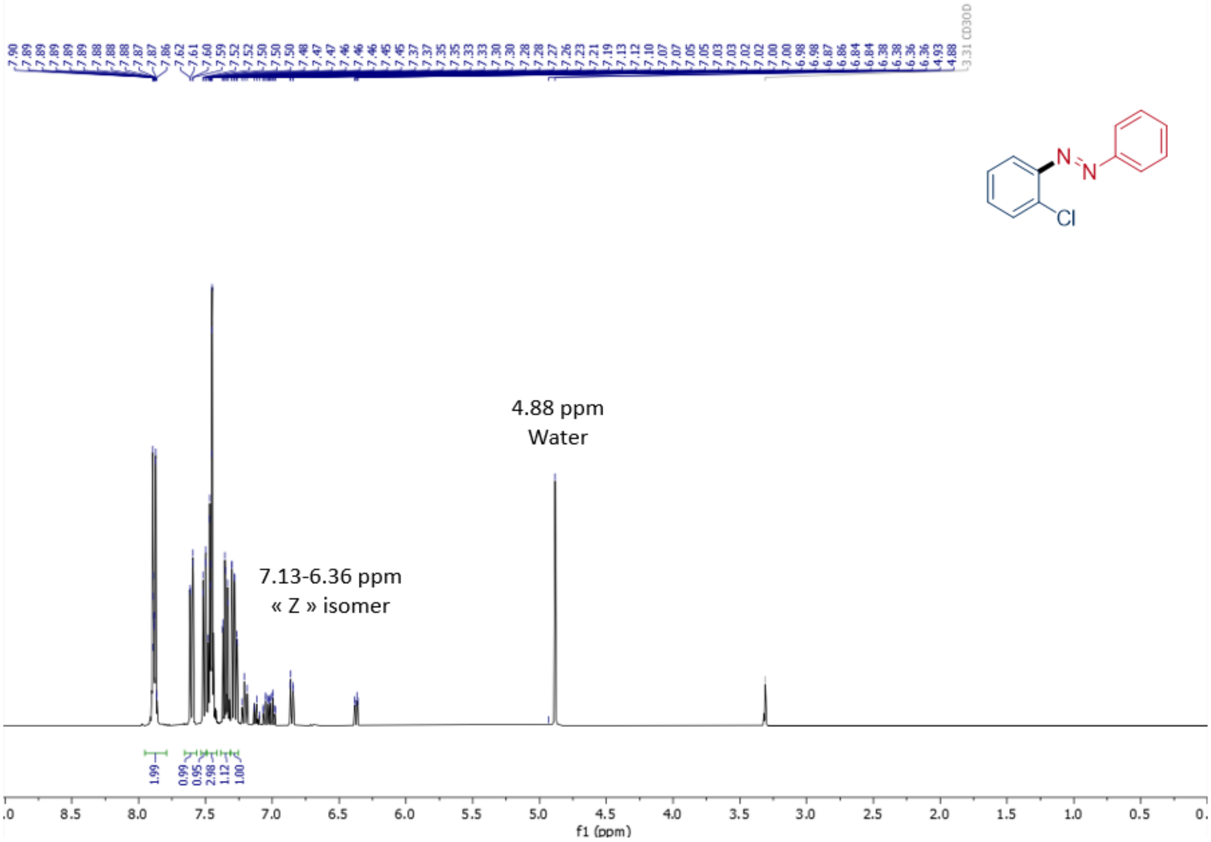


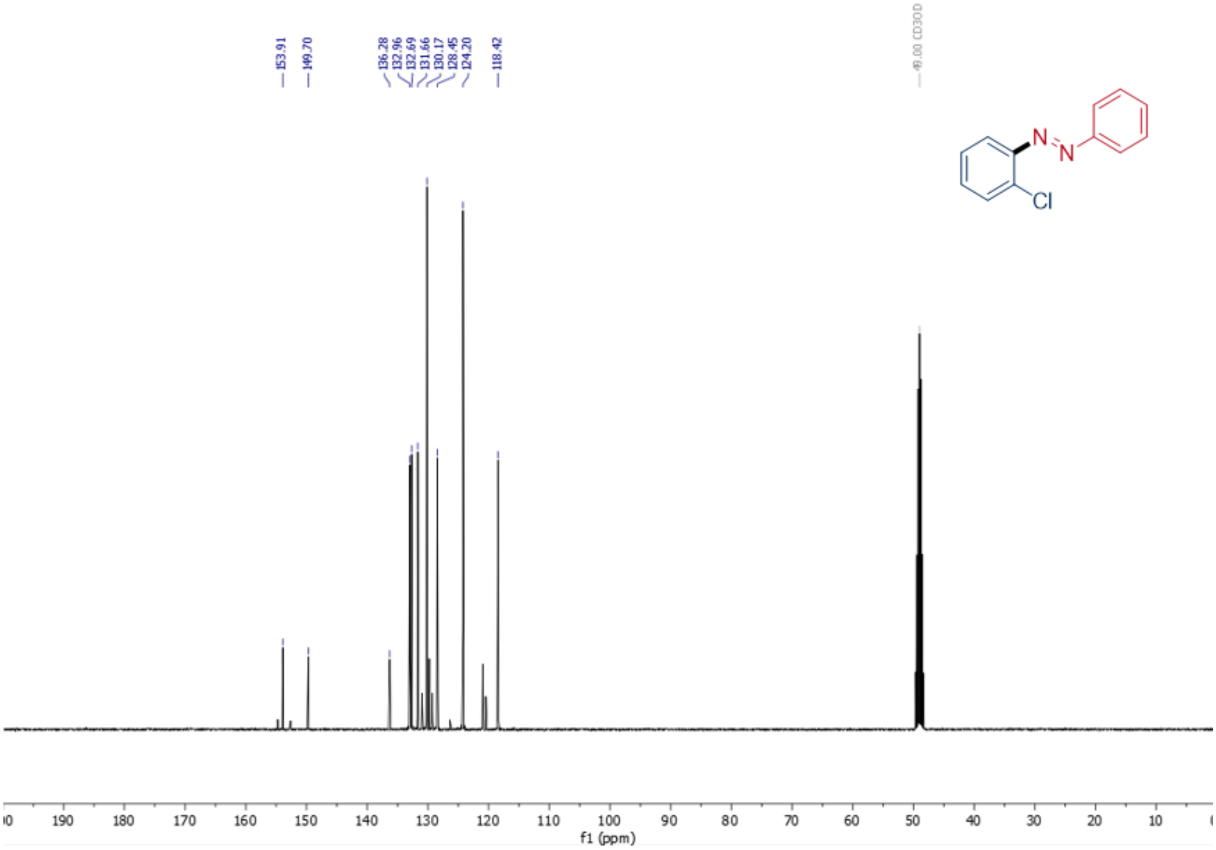

$^1H$, $^{13}C$ NMR of 3h:

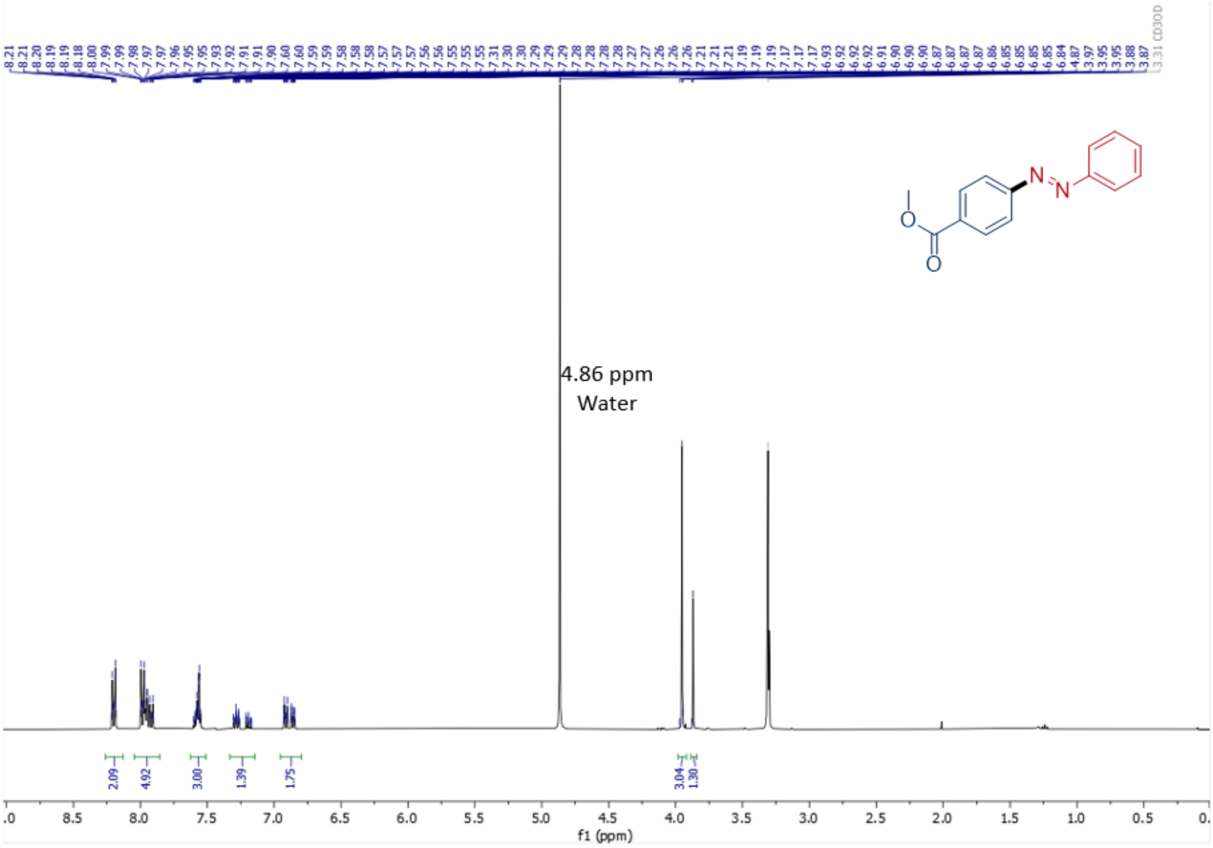


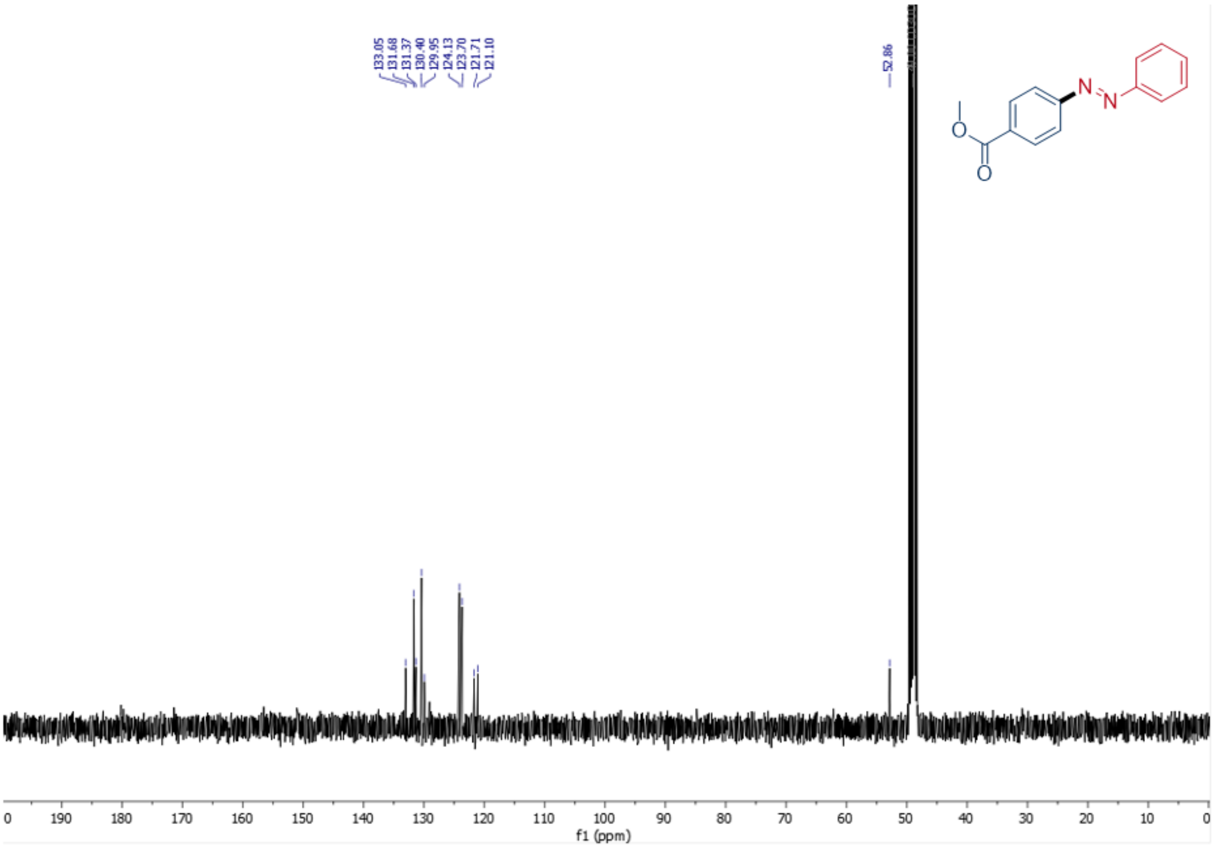

**$^{1}$H, $^{13}$C NMR of 3i:**

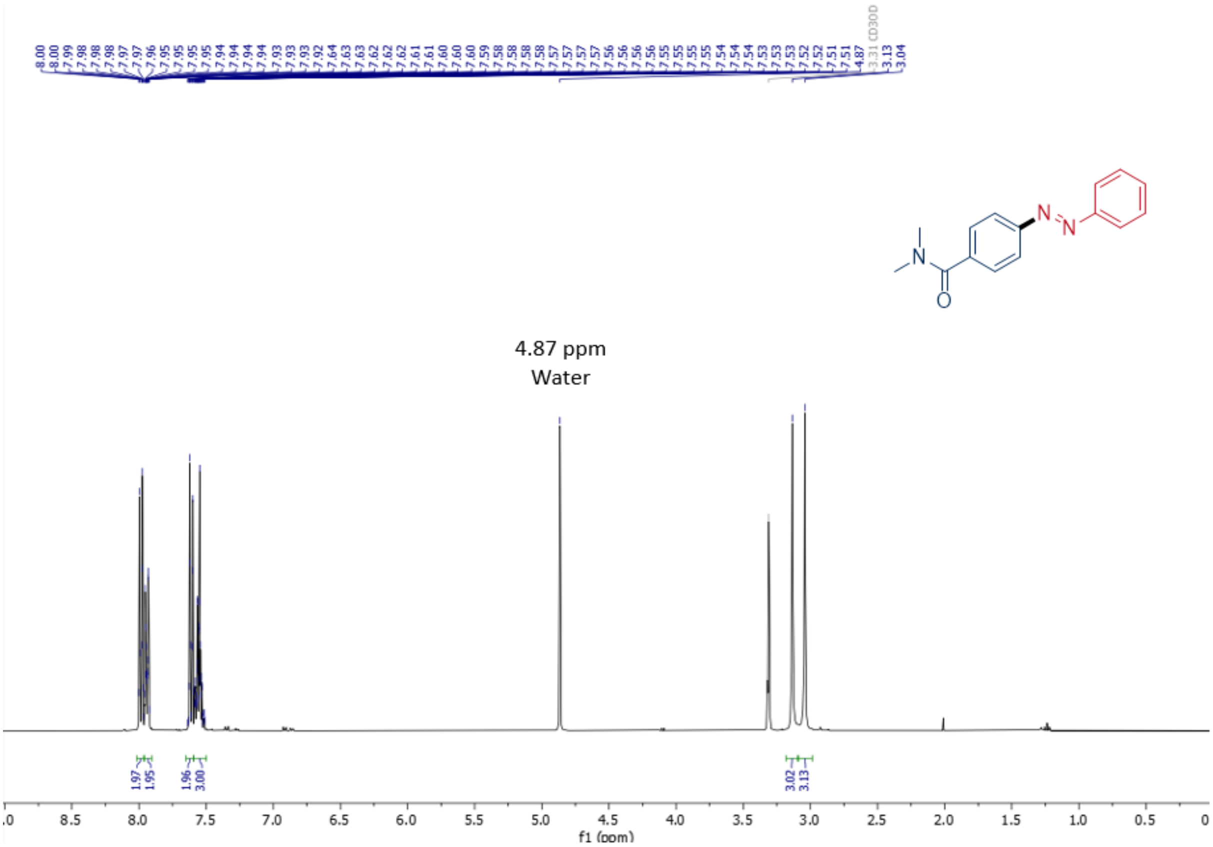


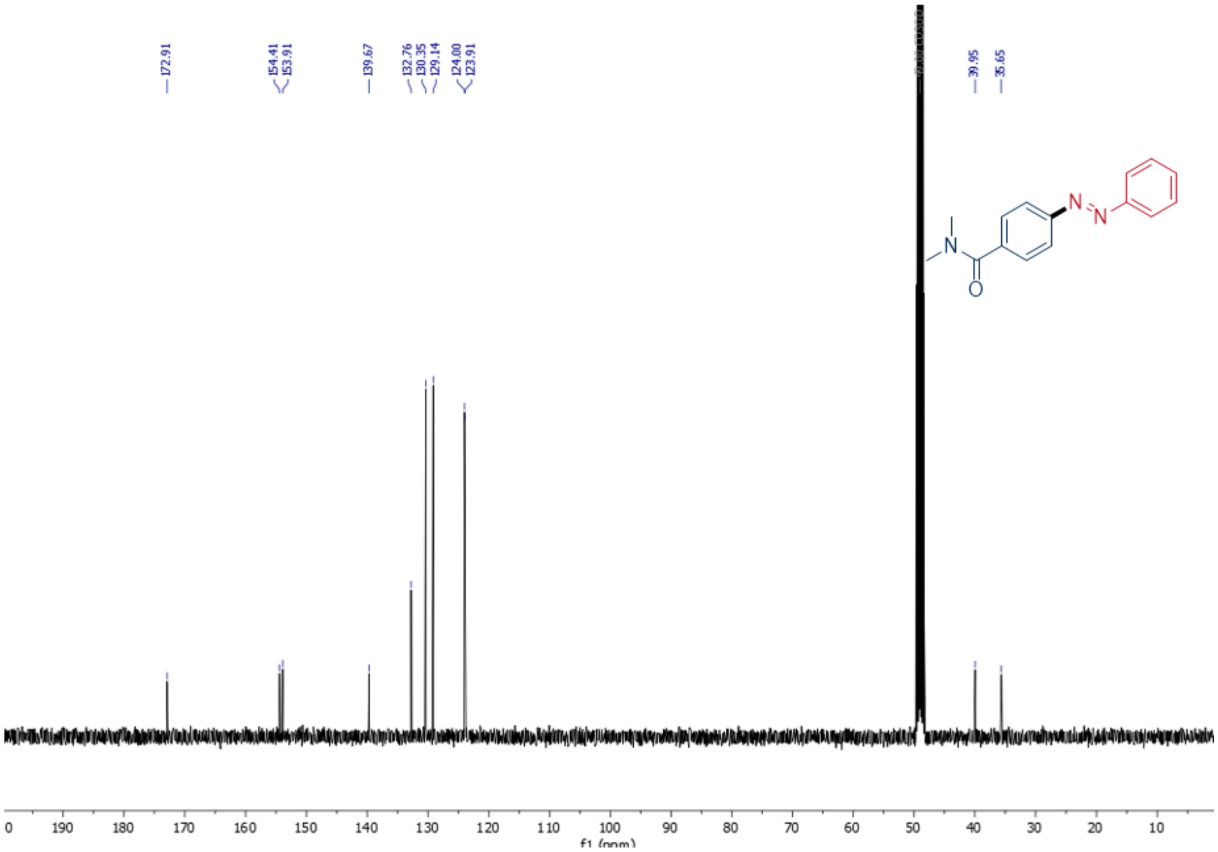

**$^{1}$H, $^{13}$C NMR of 3j:**

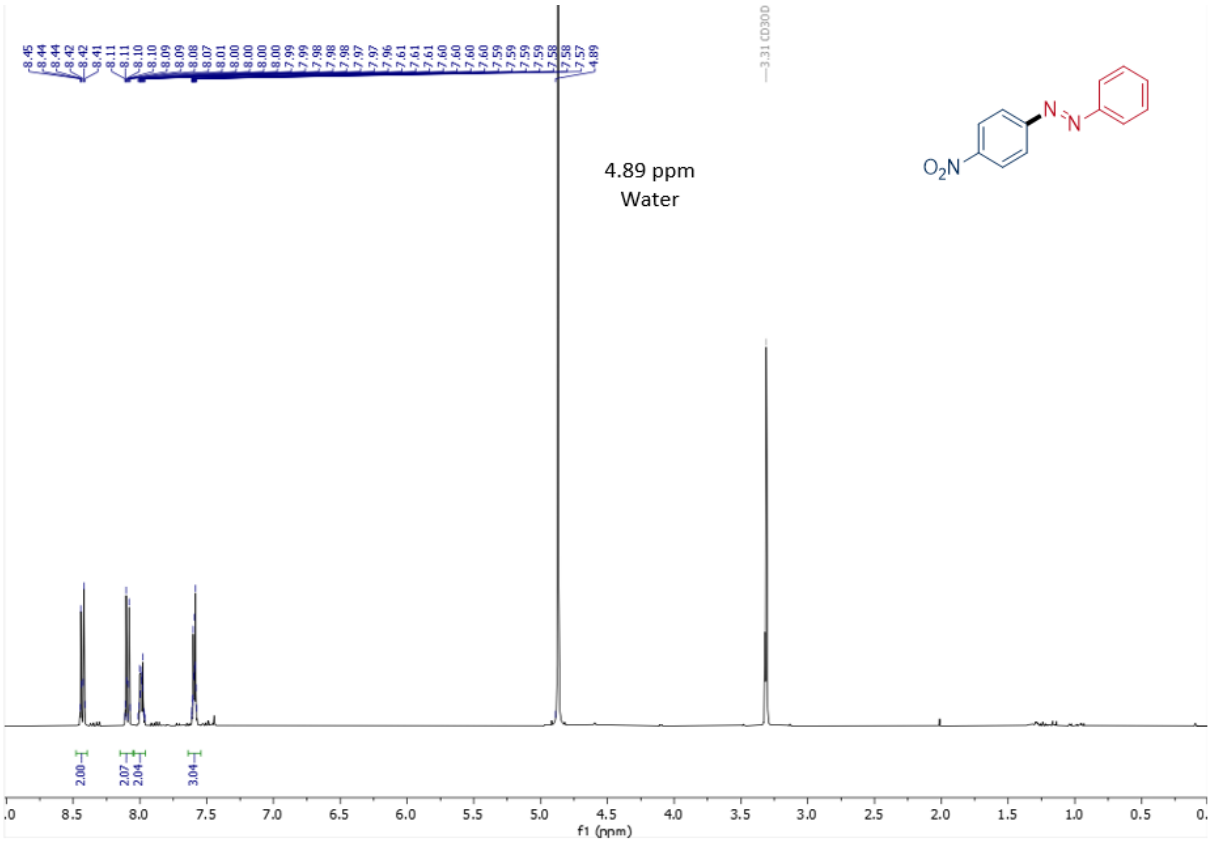


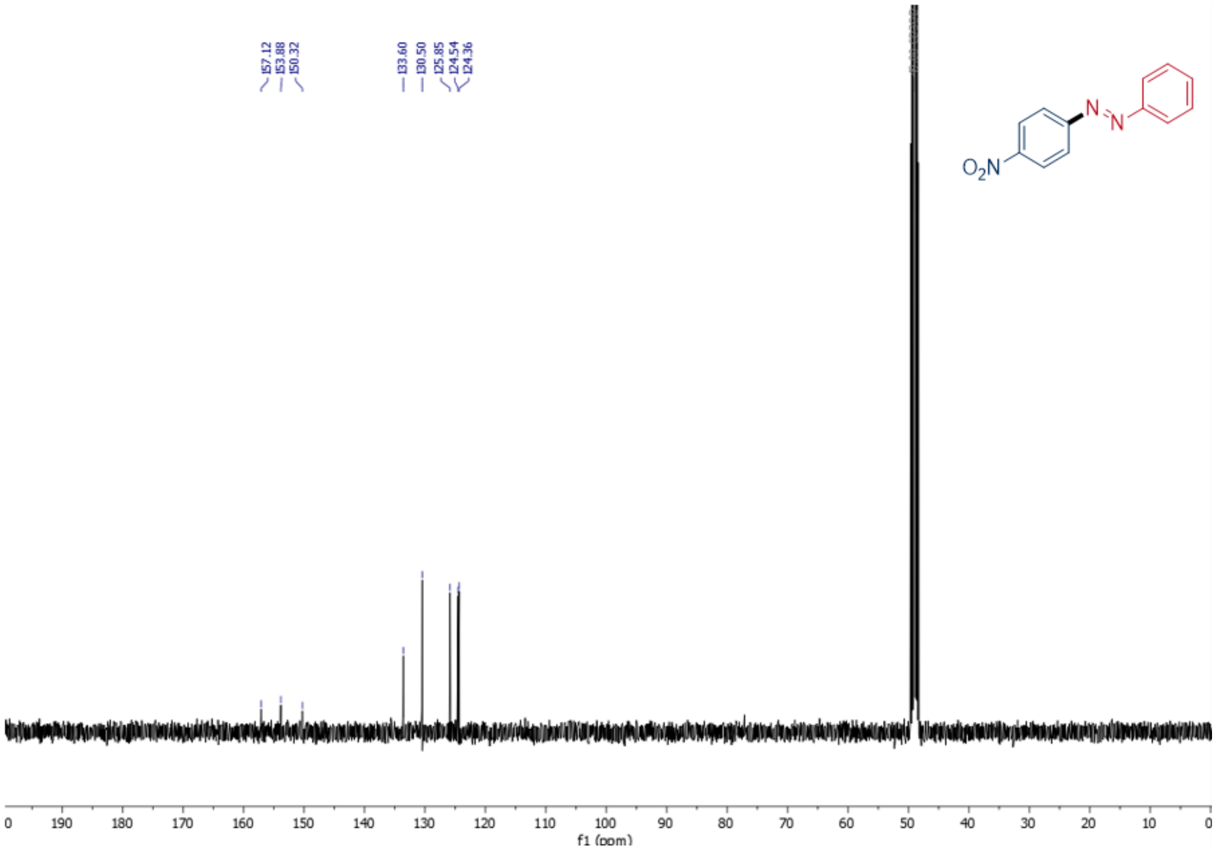

$^{1}H$, $^{13}C$ NMR of 3k:

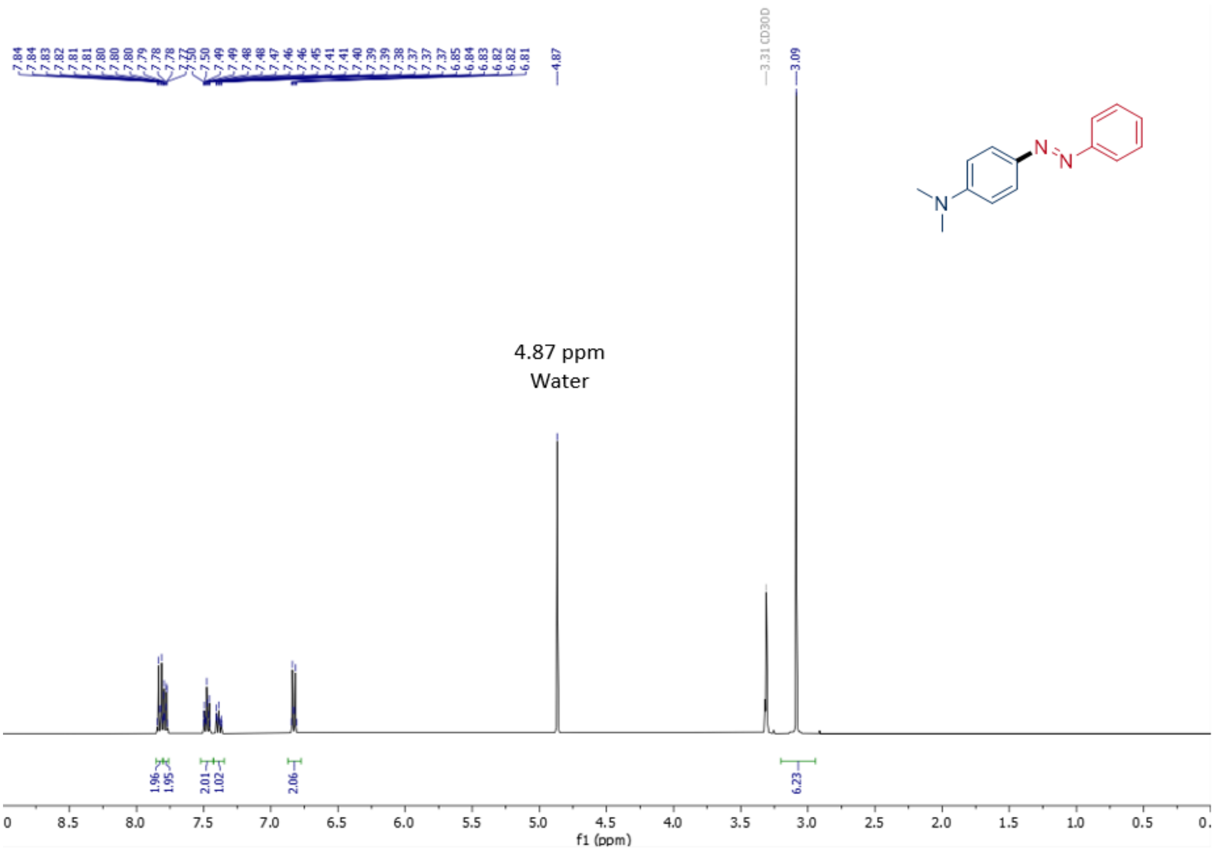


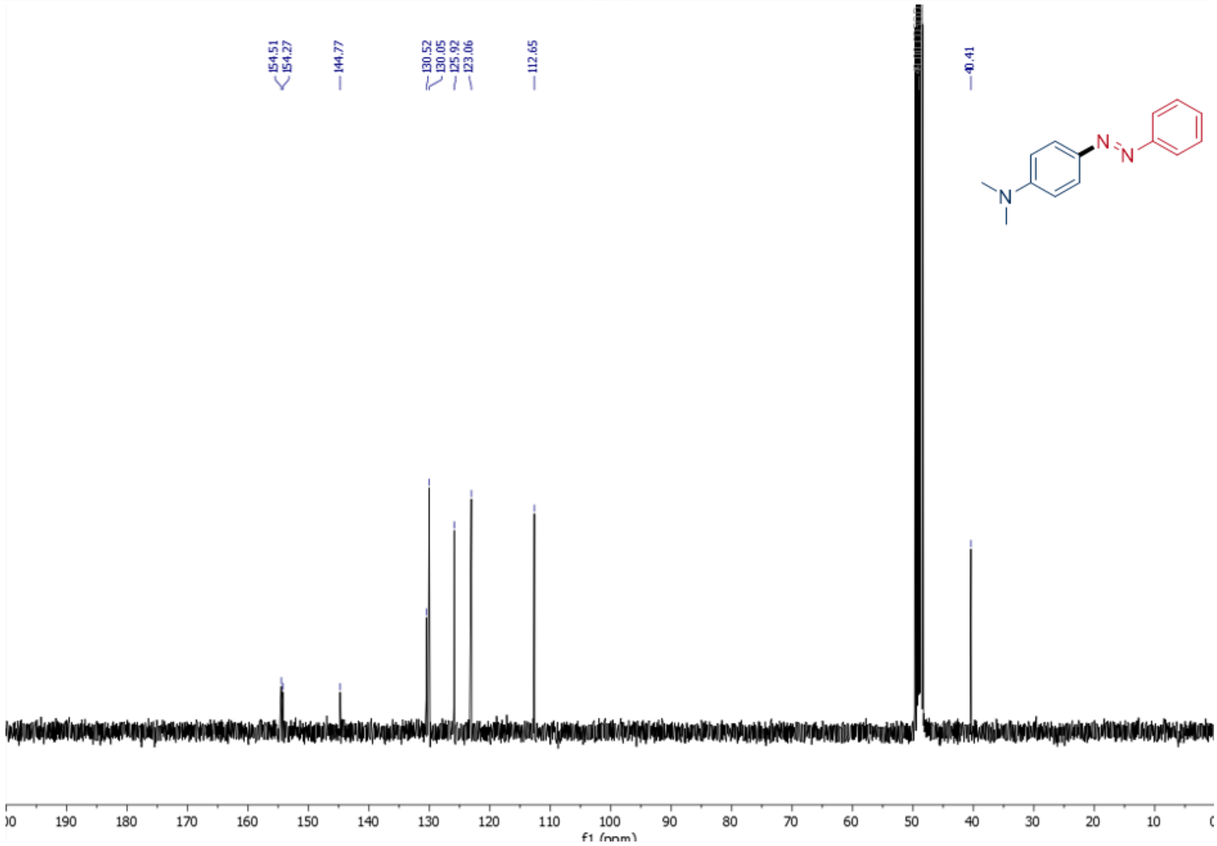

$^{1}H$, $^{13}C$ NMR of 3l:

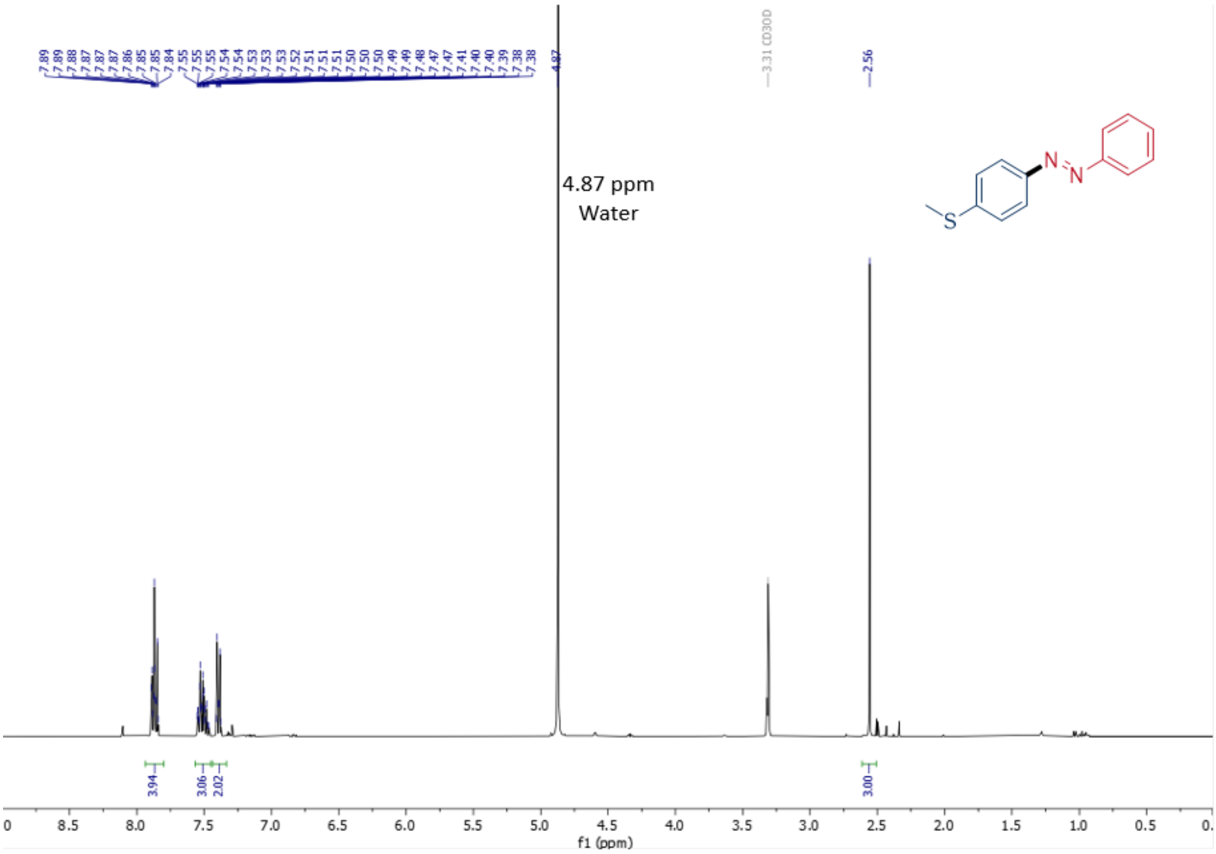


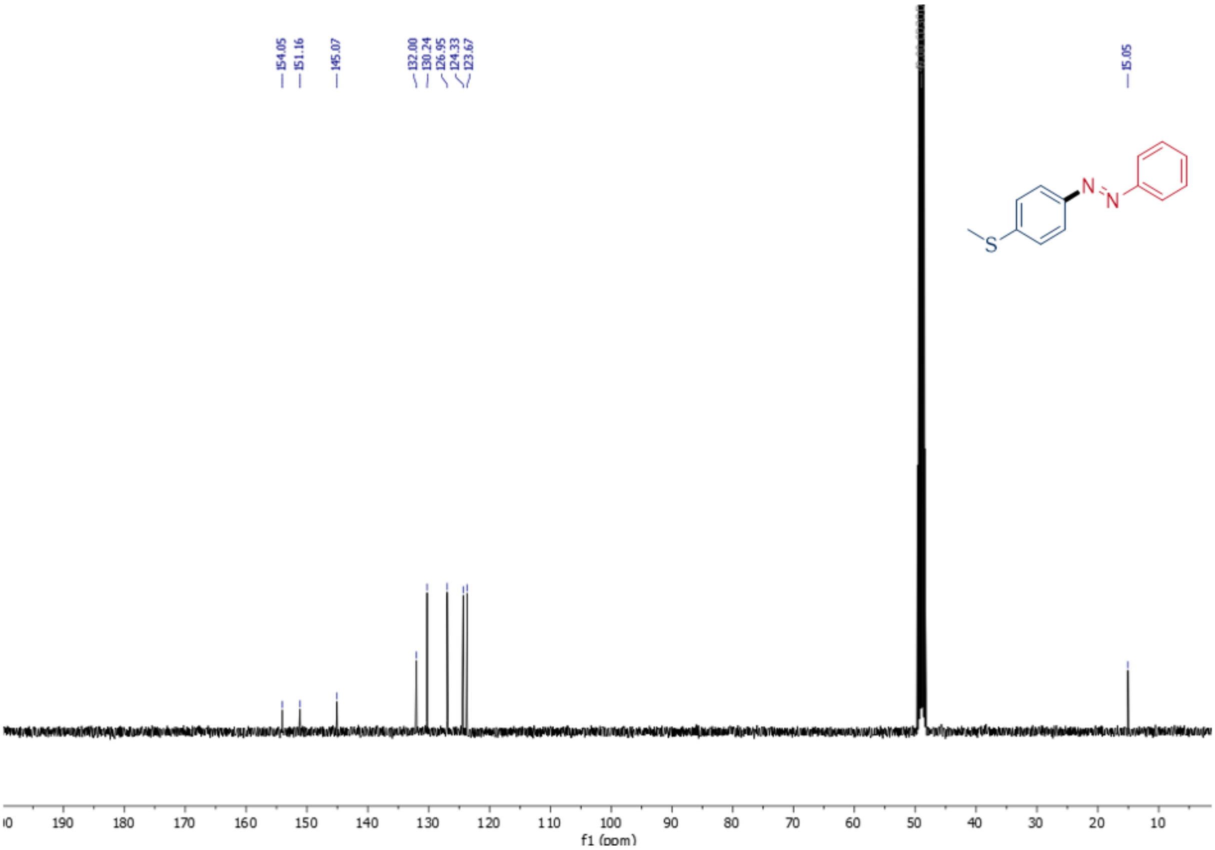

$^{1}H$, $^{13}C$ NMR of 3m:

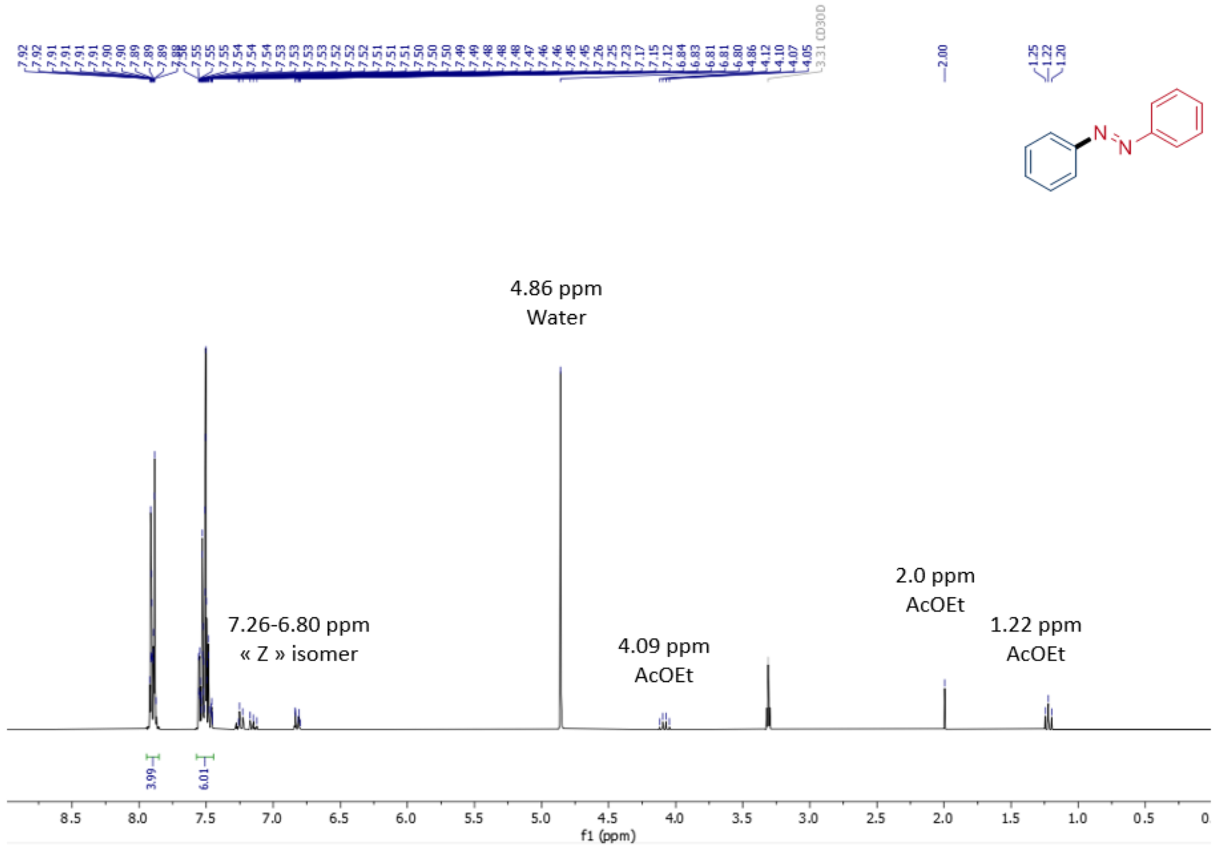


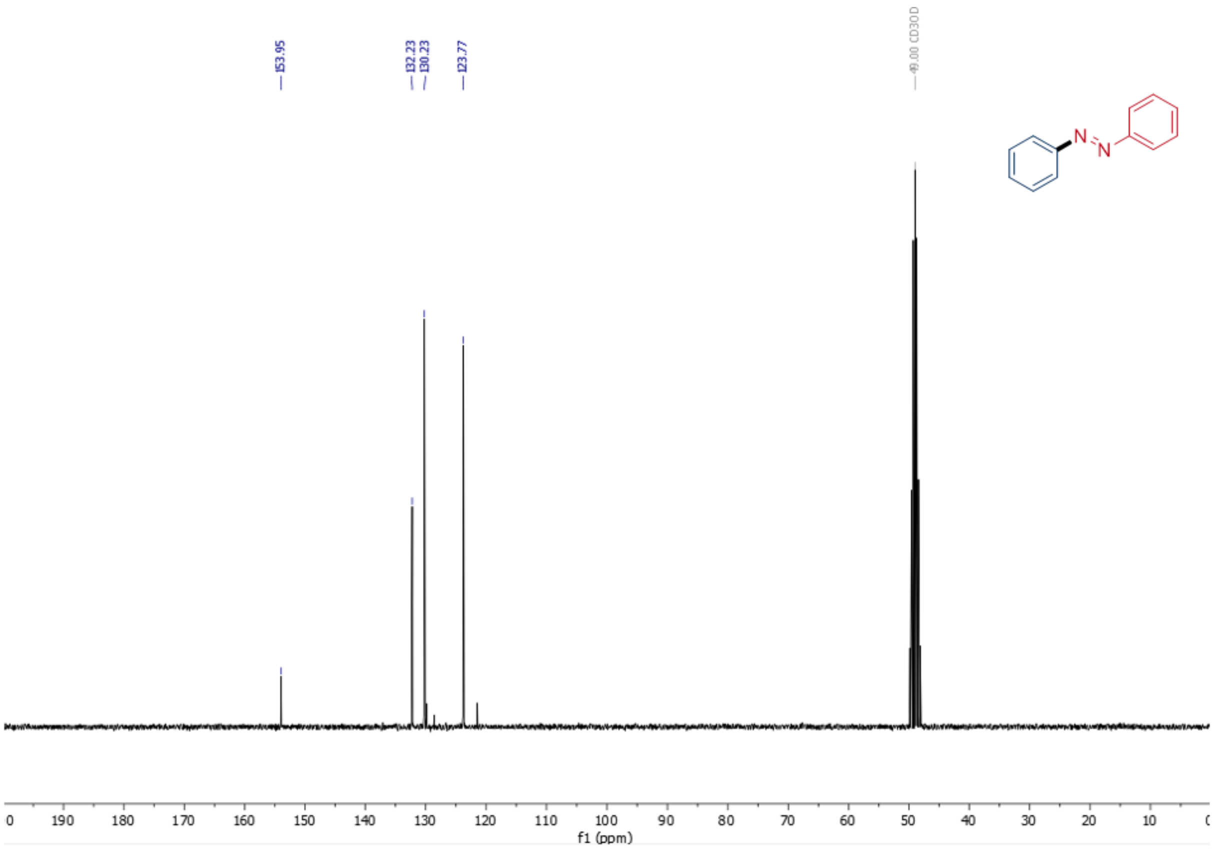

$^{1}$H, $^{13}$C NMR of 3n:

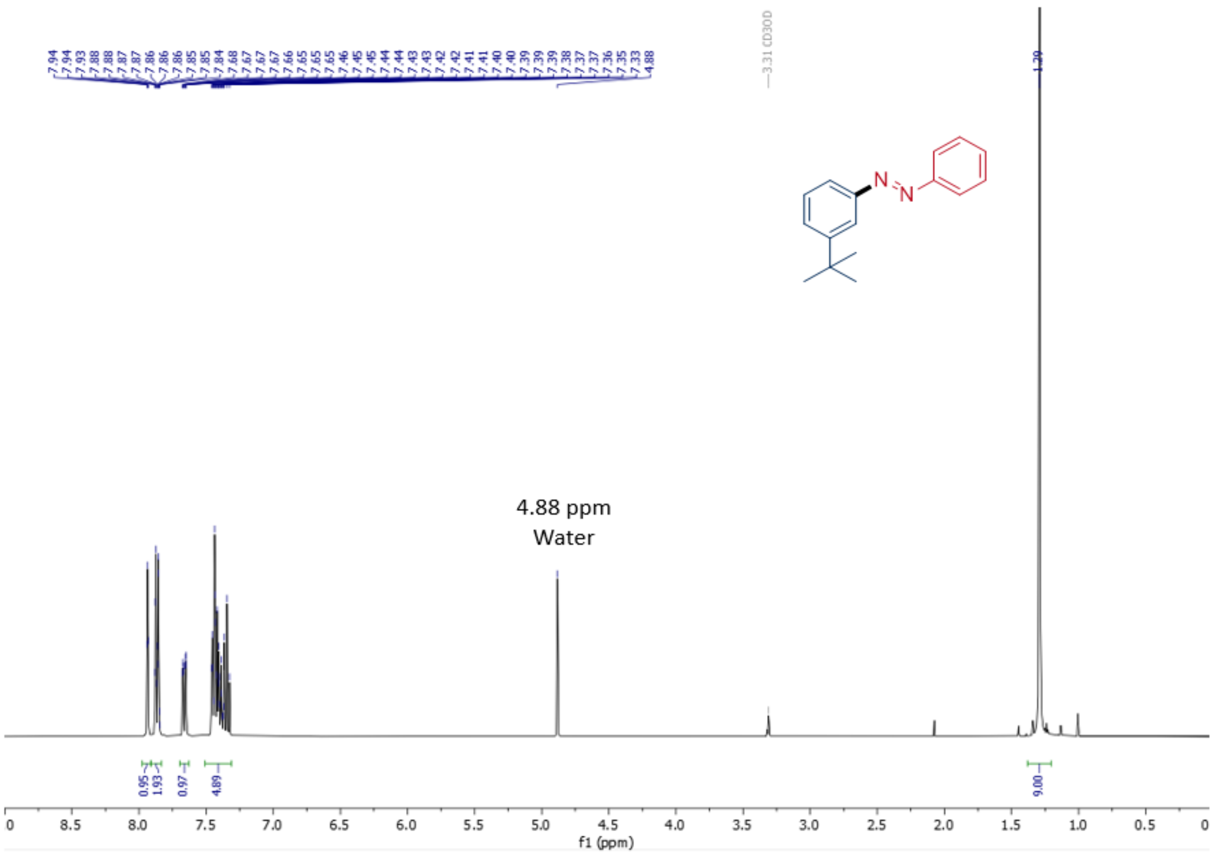


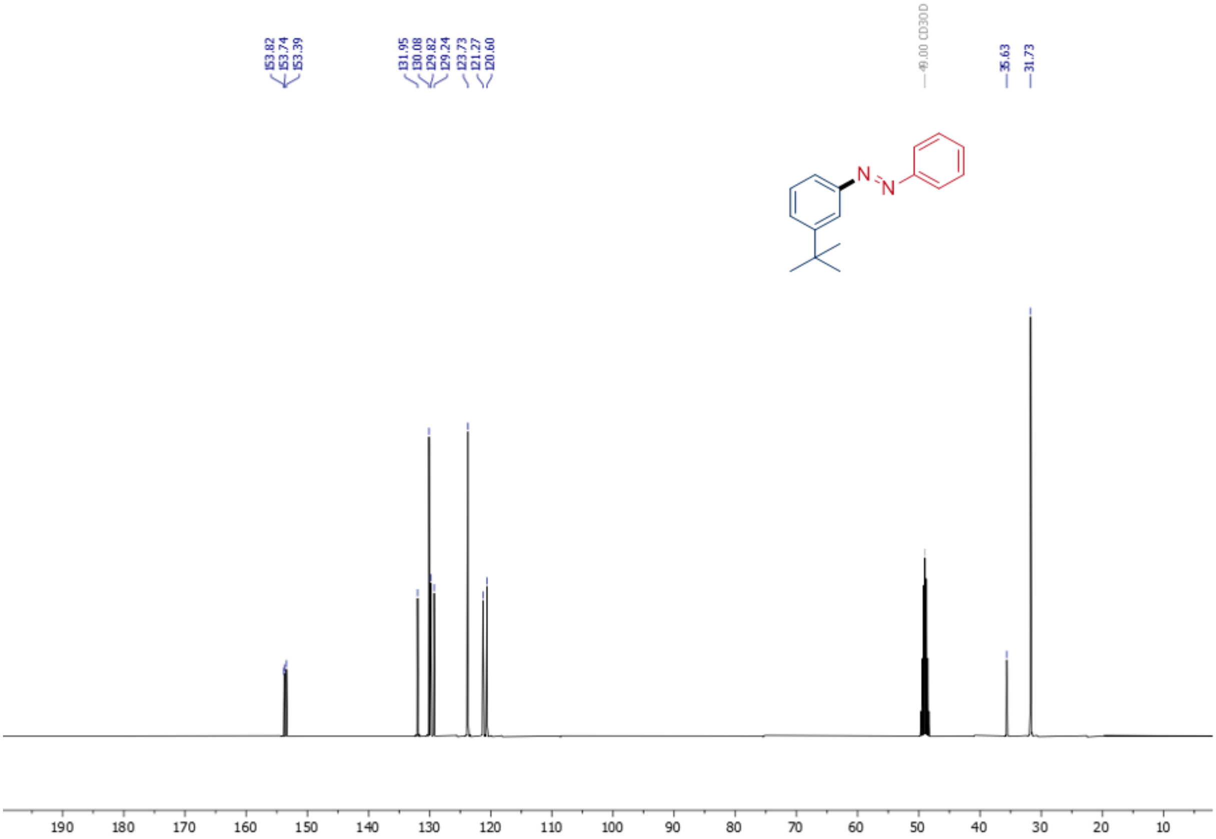

**$^{1}H$, $^{13}C$ NMR of 3o:**

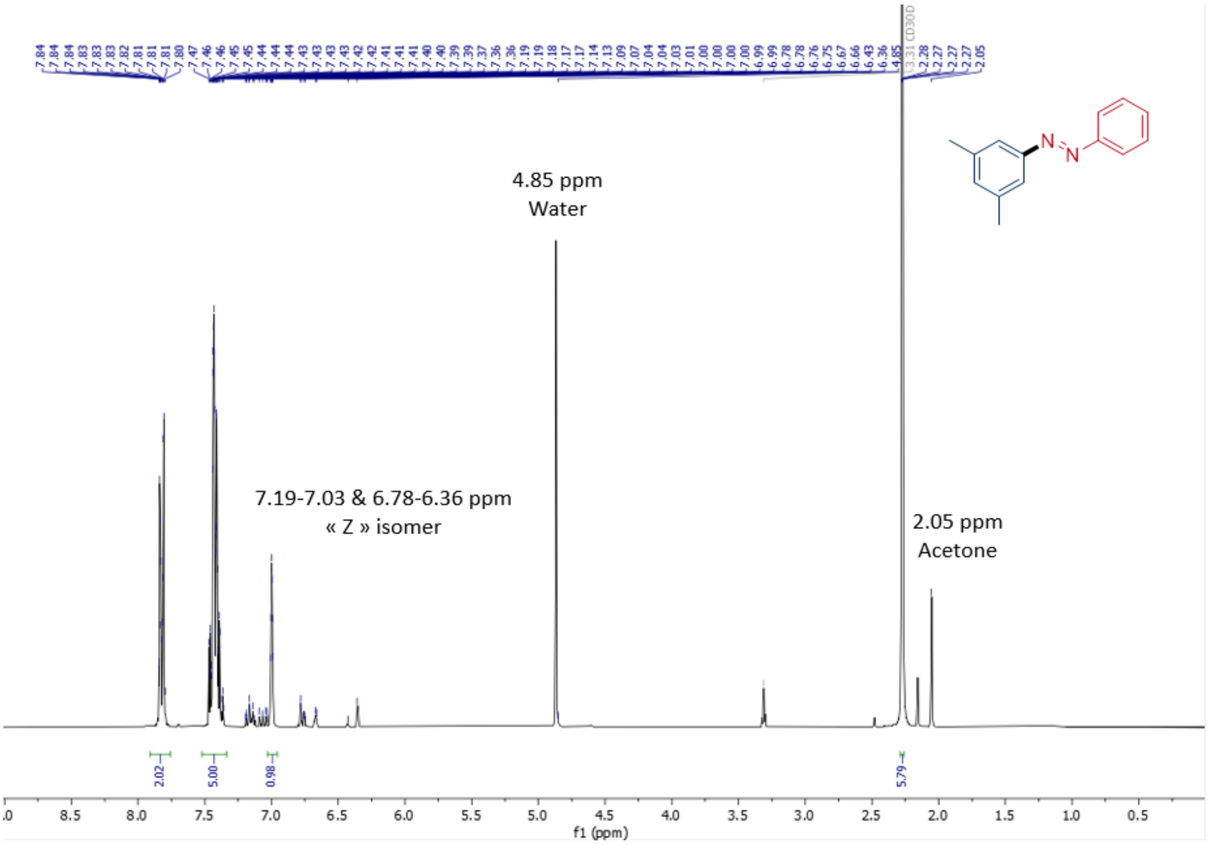


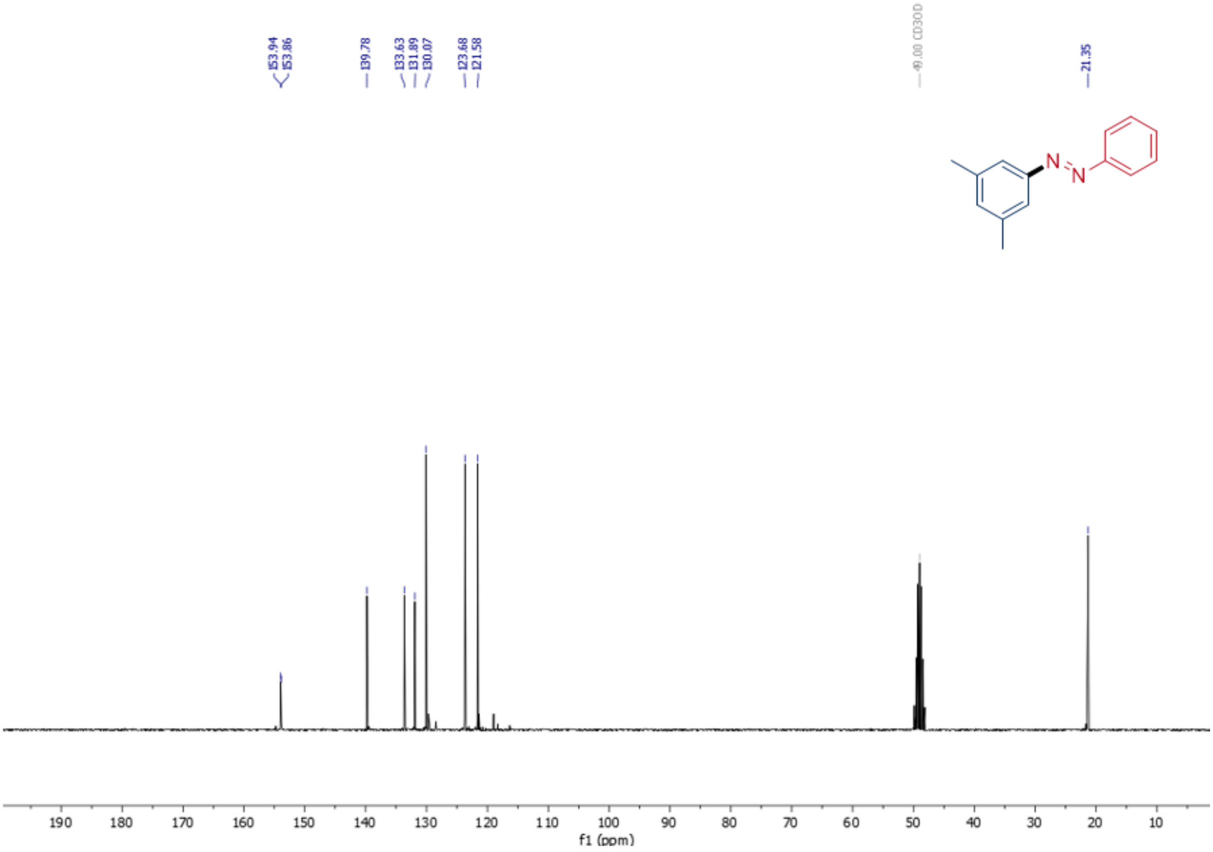

$^{1}H$, $^{13}C$ NMR of 3p:

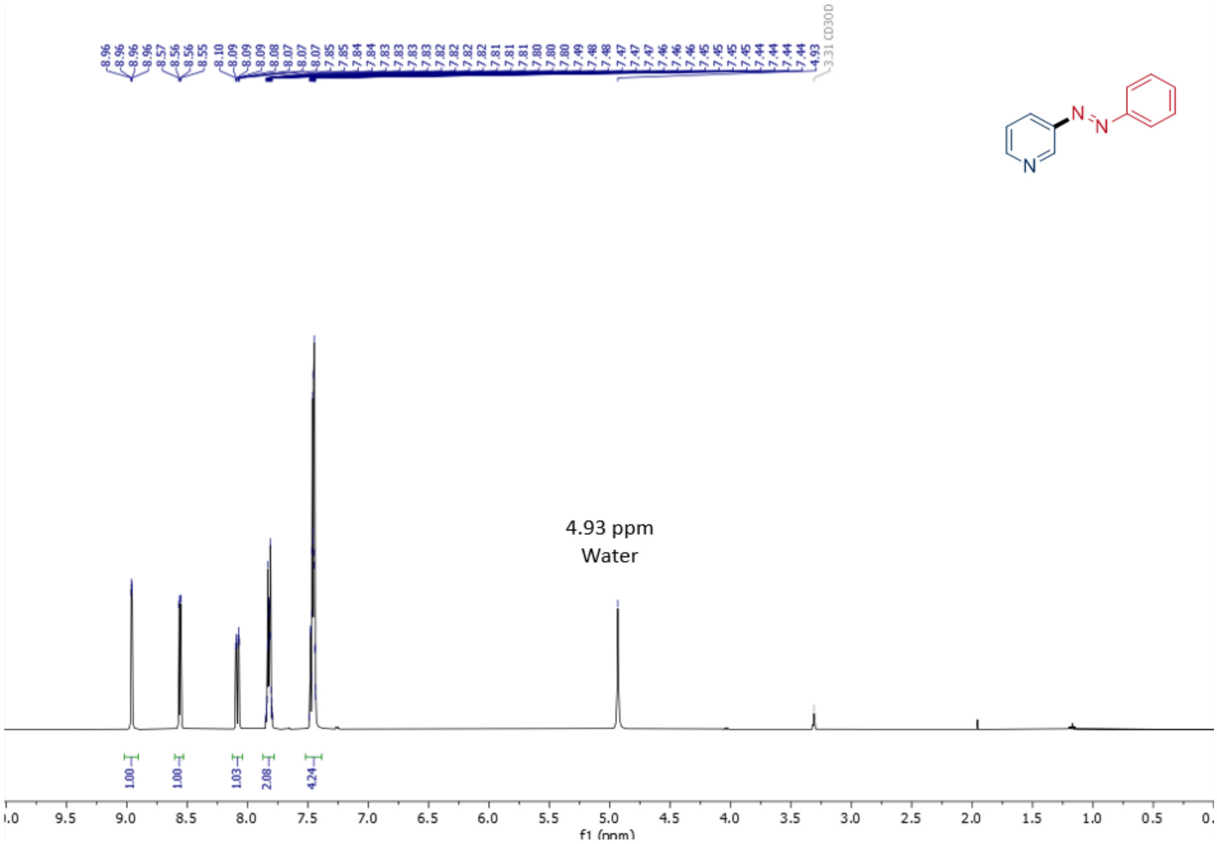


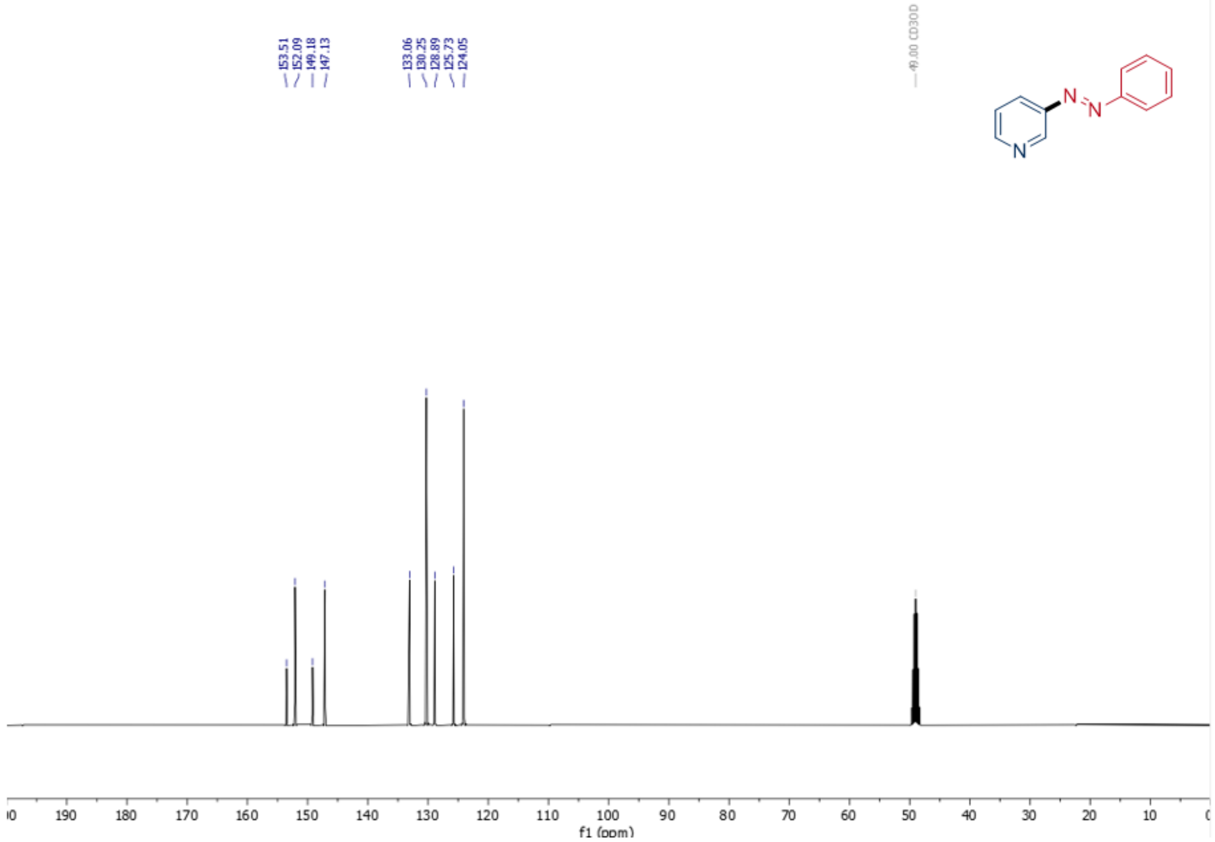

**$^{1}H$, $^{13}C$ NMR of 3q:**

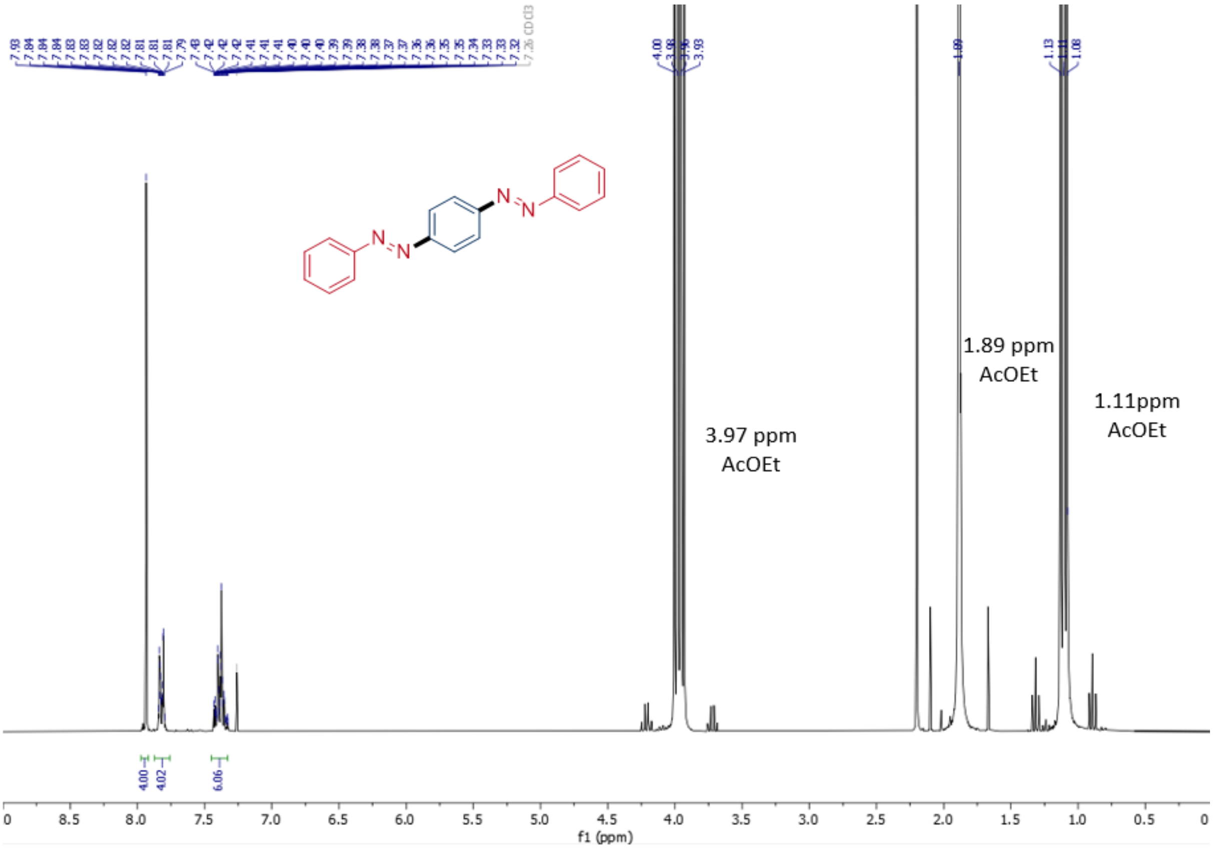


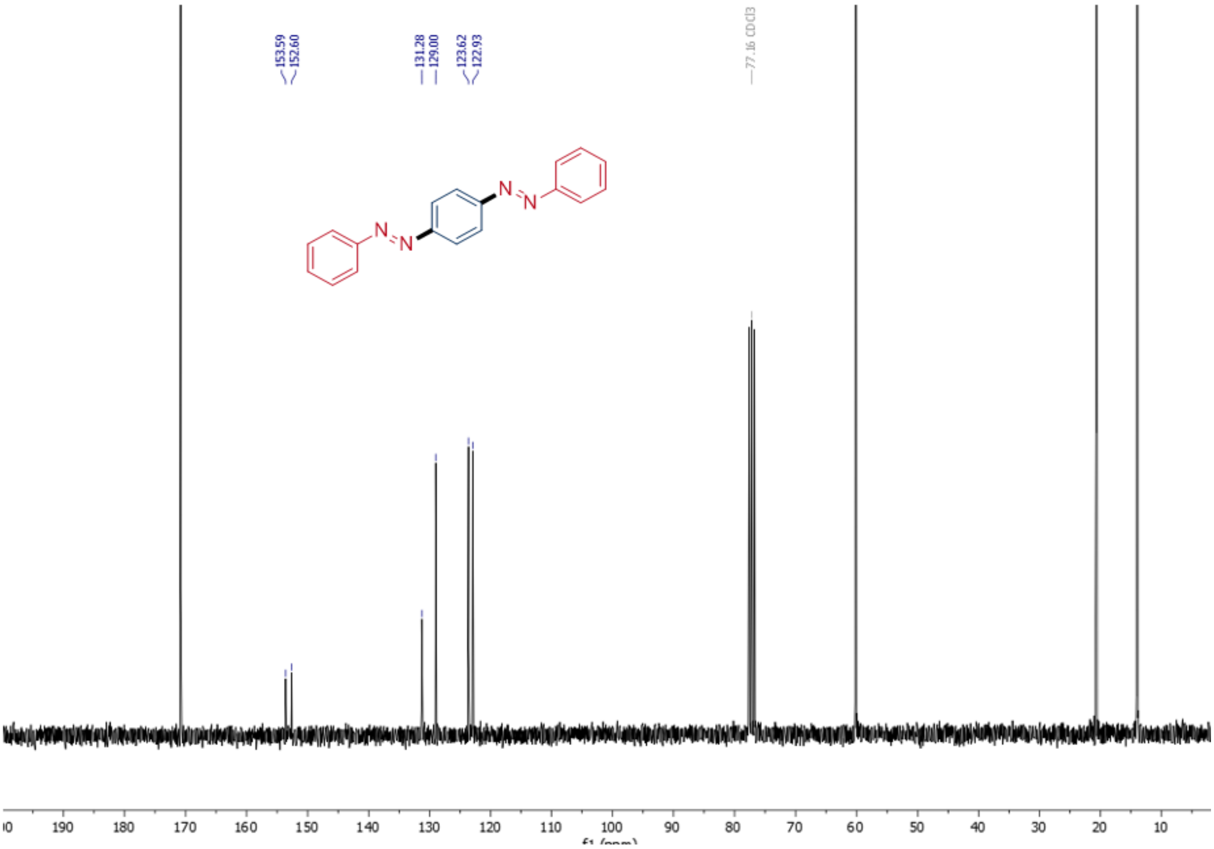

**$^{1}H$, $^{13}C$, $^{19}F$ NMR of 4a:**

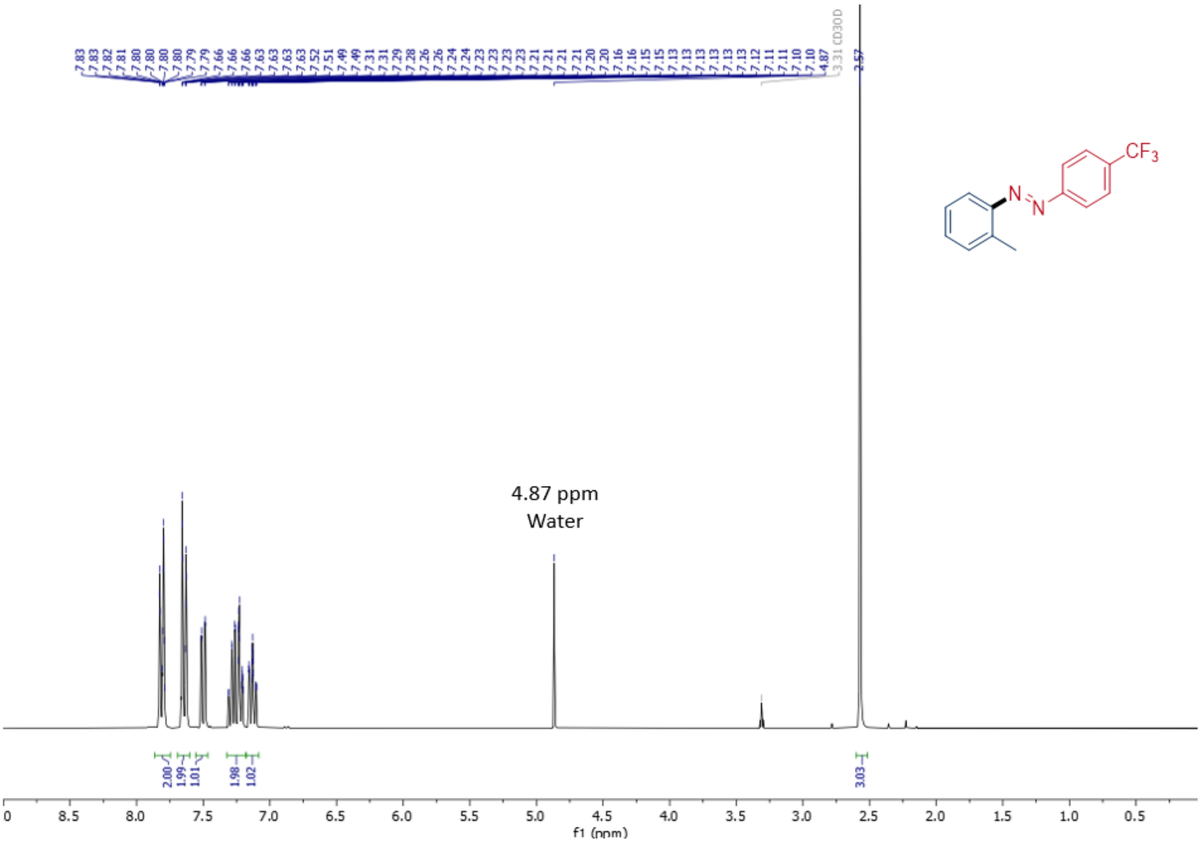


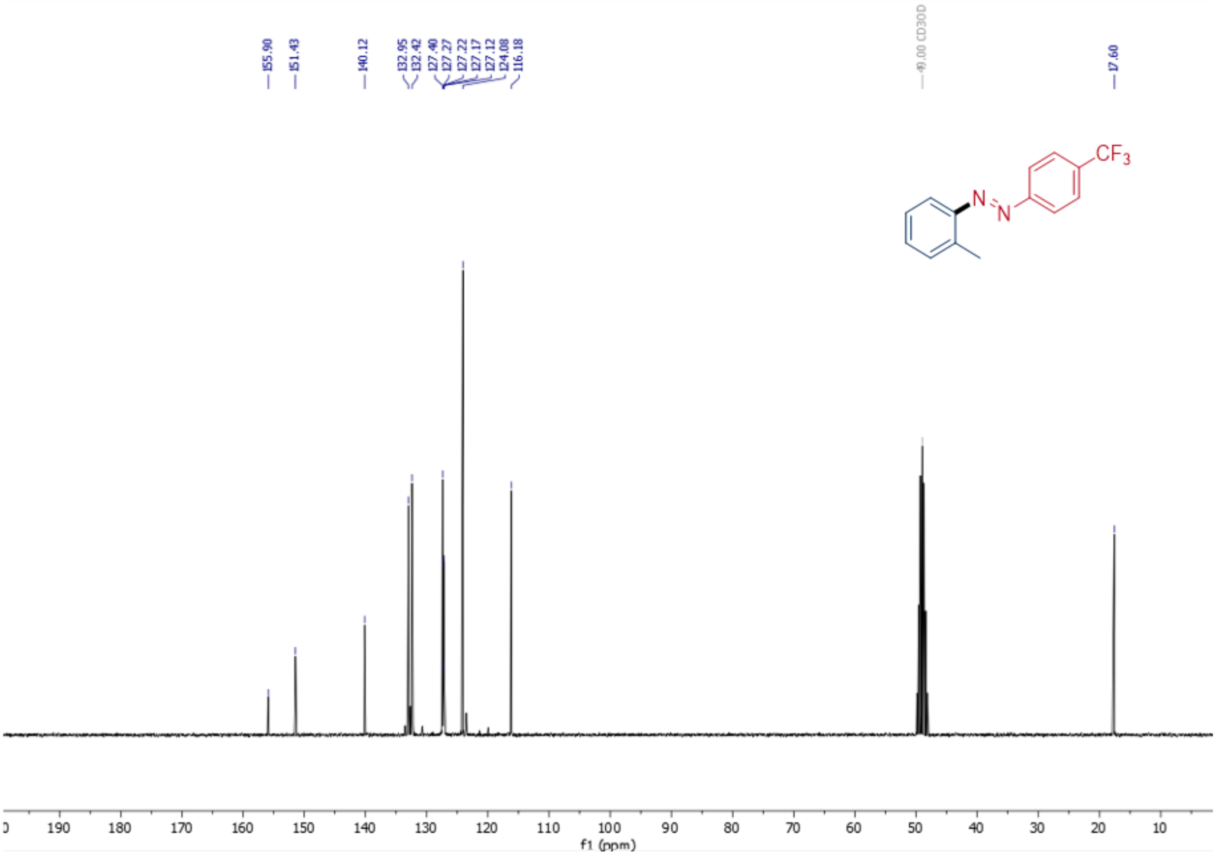

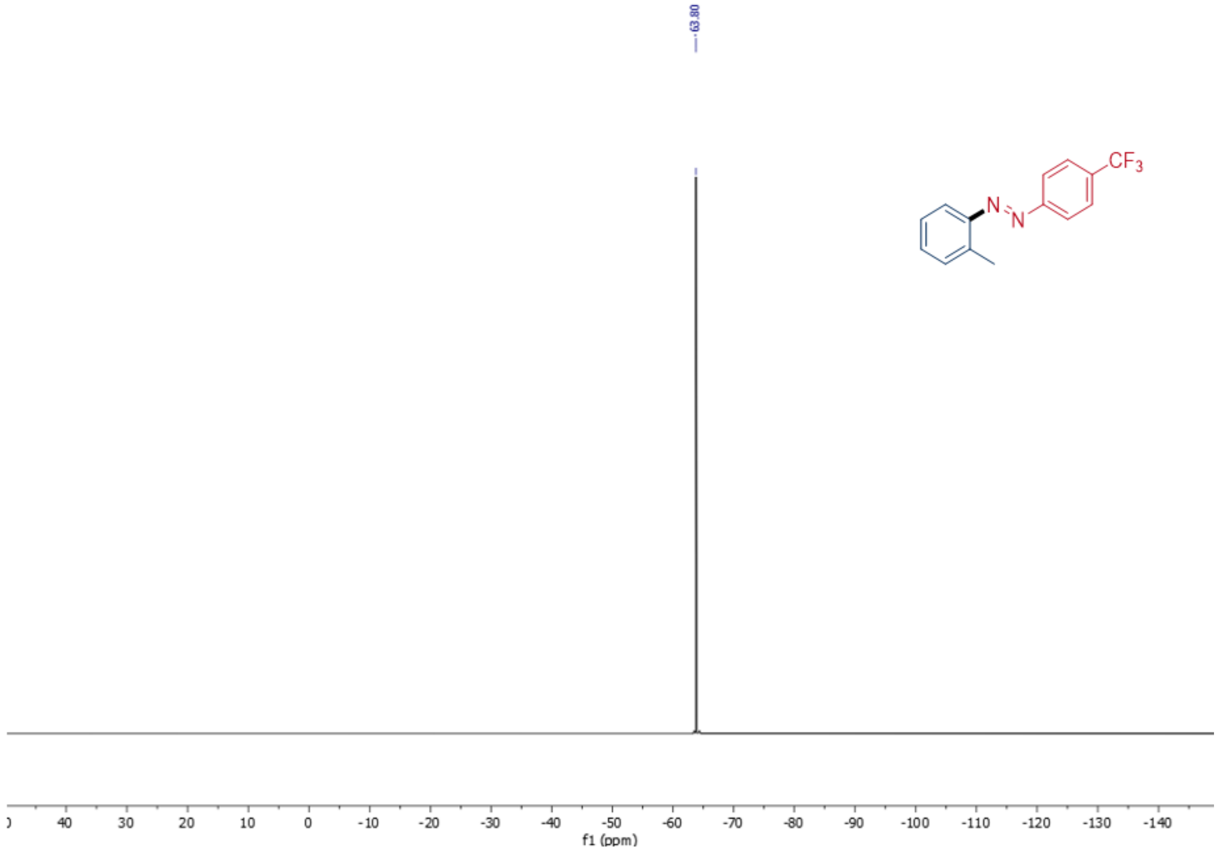
-63.80
CF3
N
N
40
30
20
10
0
-10
-20
-30
-40
-50
-60
-70
-80
-90
-100
-110
-120
-130
-140
f1 (ppm)

**$^1H$, $^{13}C$ NMR of 4b:**

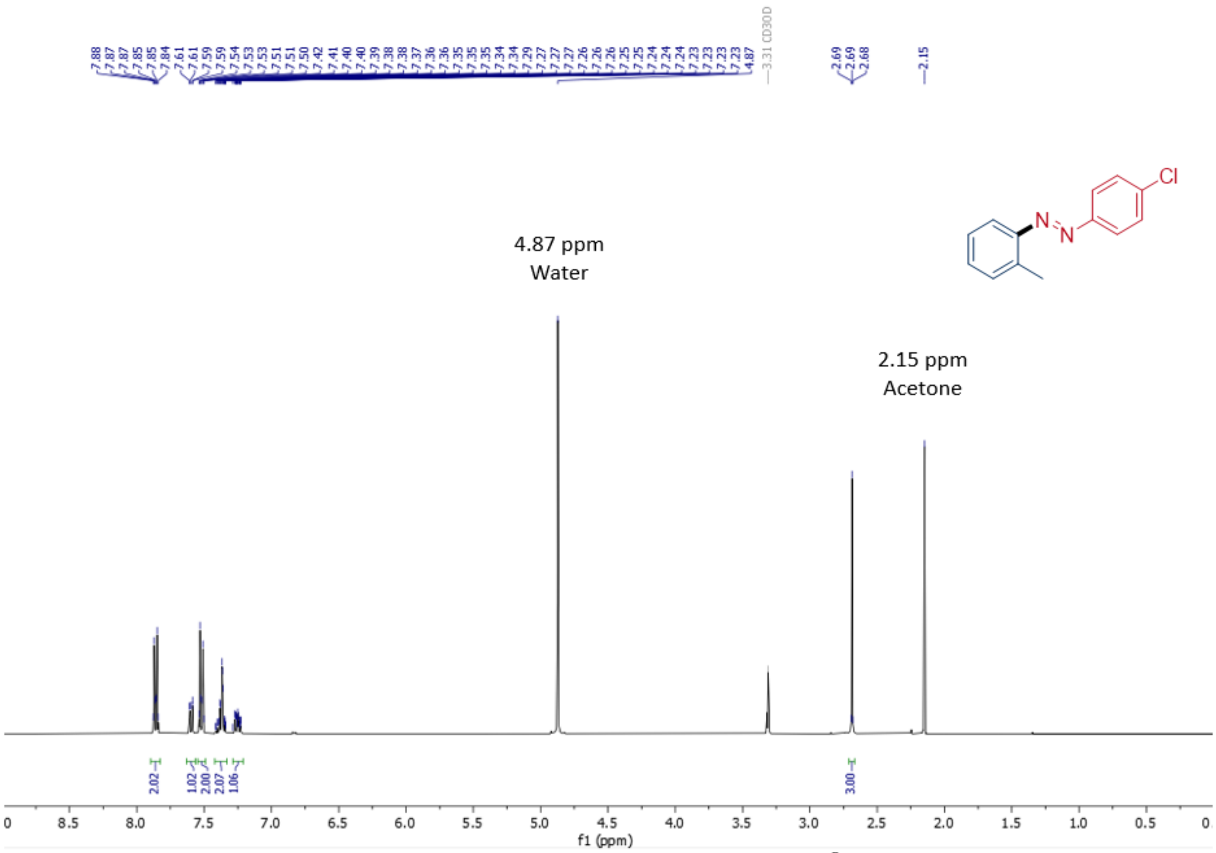


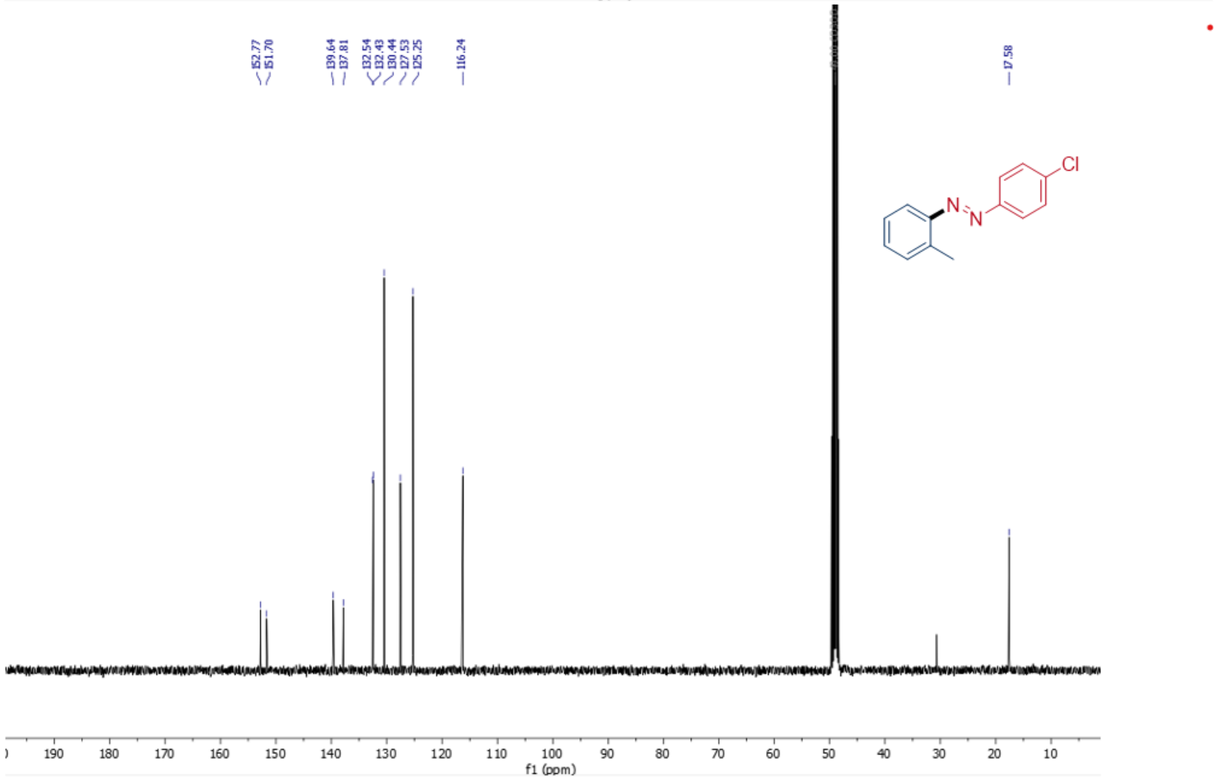

**$^{1}$H, $^{13}$C NMR of 4c:**

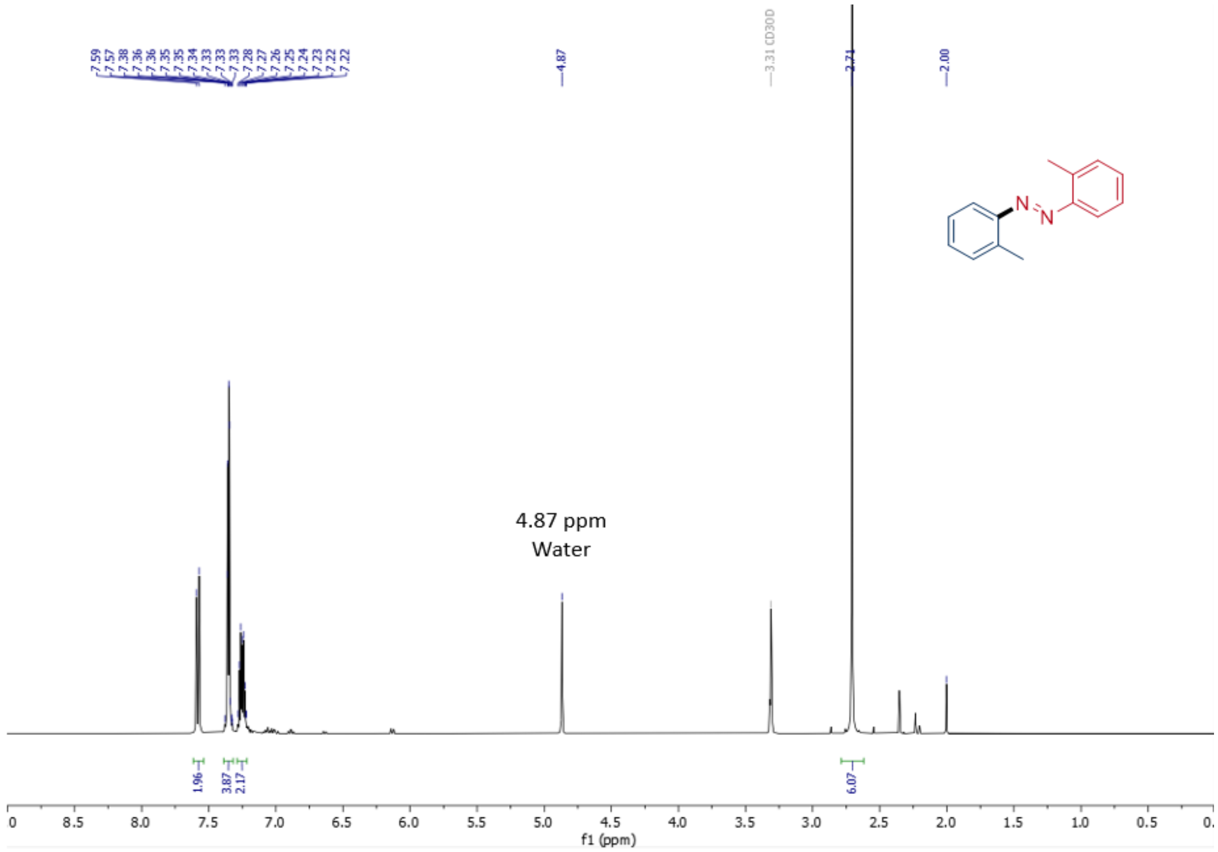


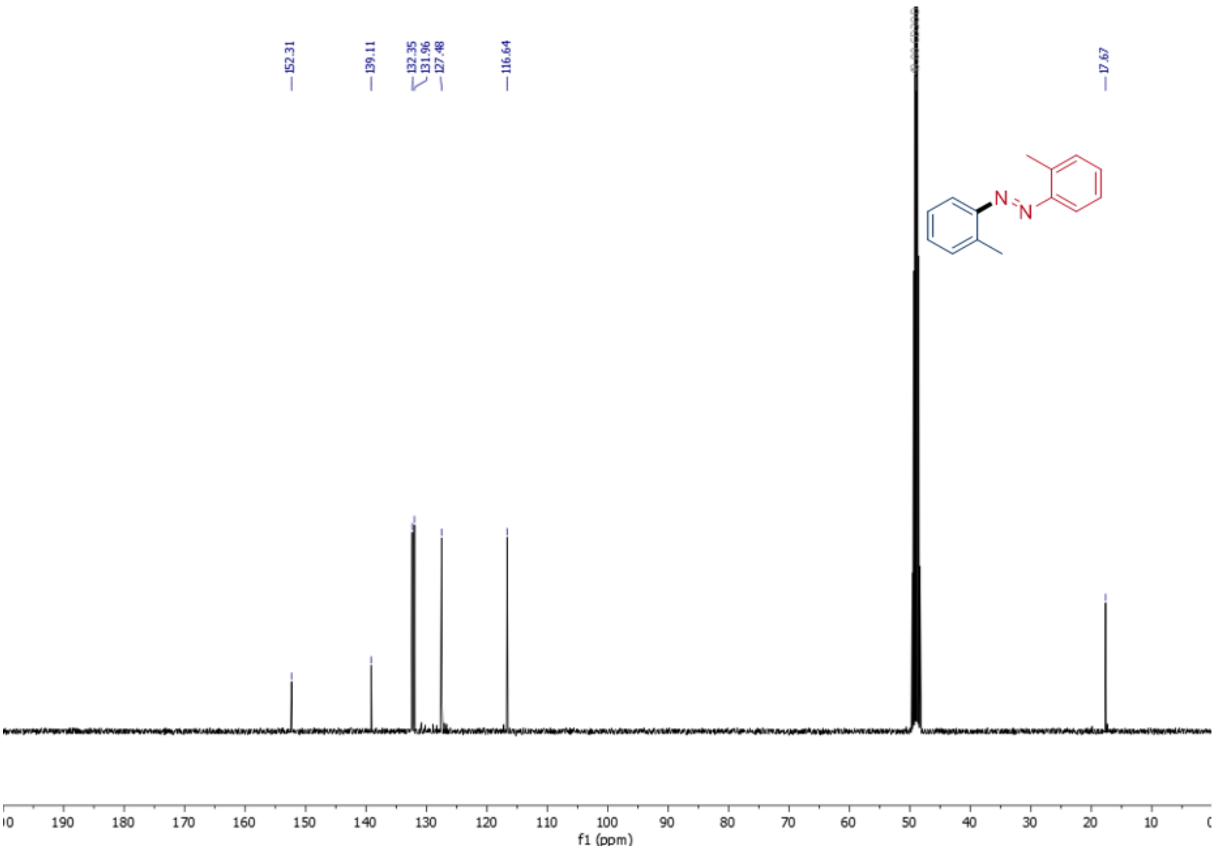

**$^{1}H$, $^{13}C$, $^{19}F$ NMR of 4d:**

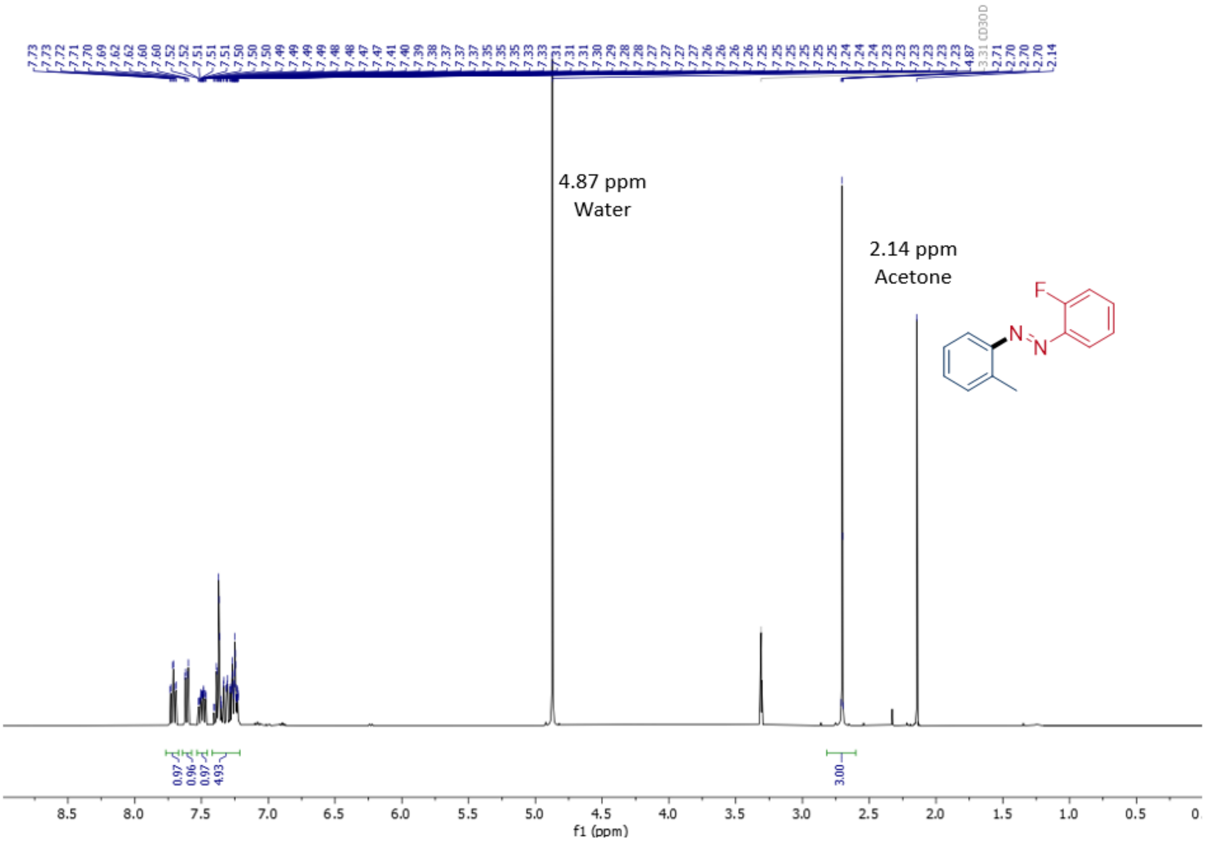


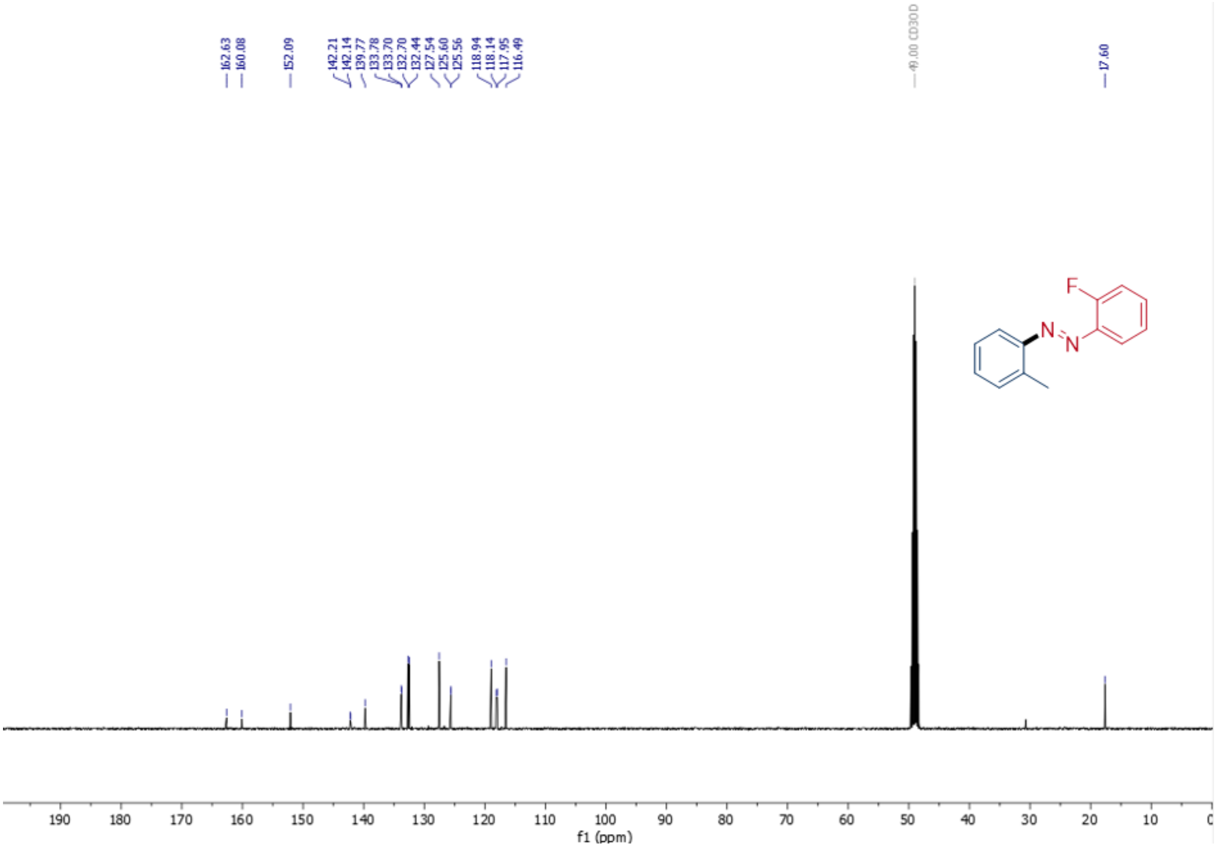

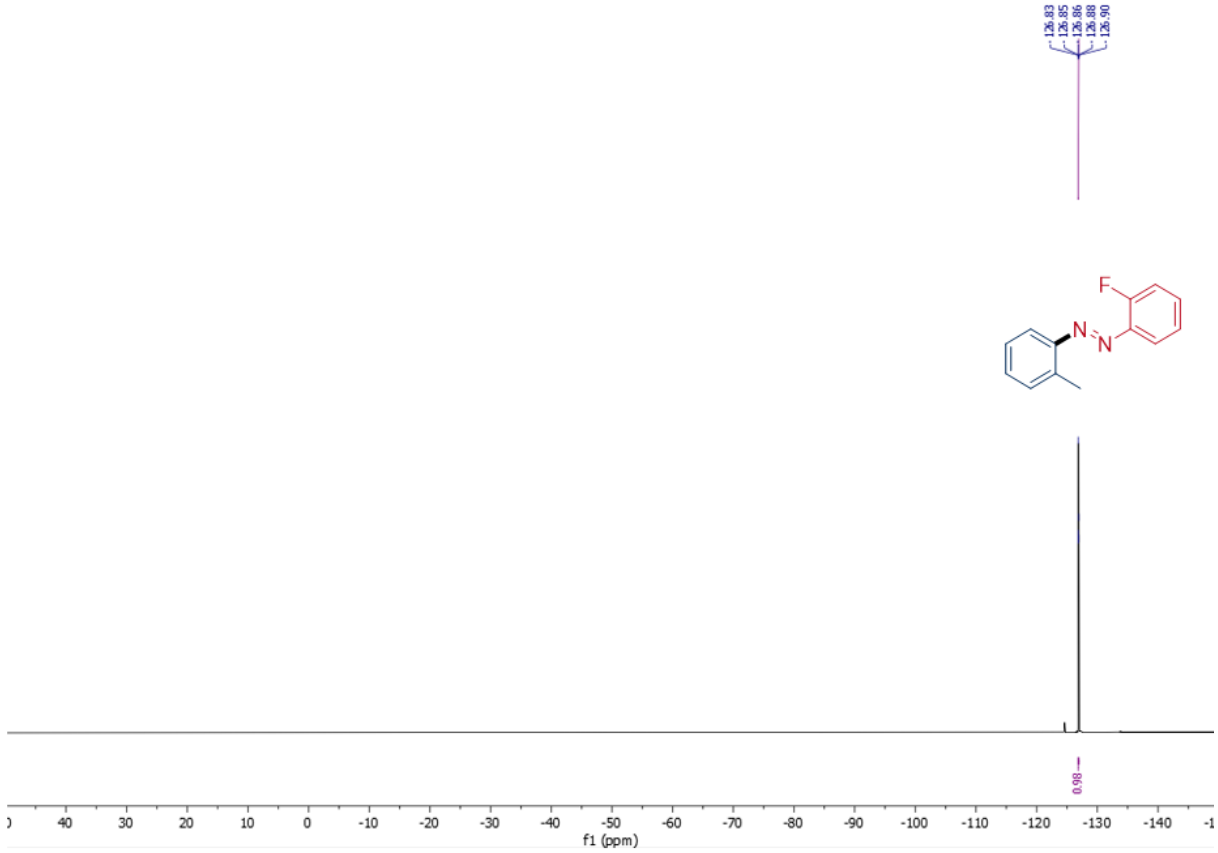
-126.83
-126.85
-126.86
-126.88
-126.90
F
N
N
0.98
40
30
20
10
0
-10
-20
-30
-40
-50
-60
-70
-80
-90
-100
-110
-120
-130
-140
f1 (ppm)

$^{1}H$, $^{13}C$, $^{19}F$ NMR of 4e:

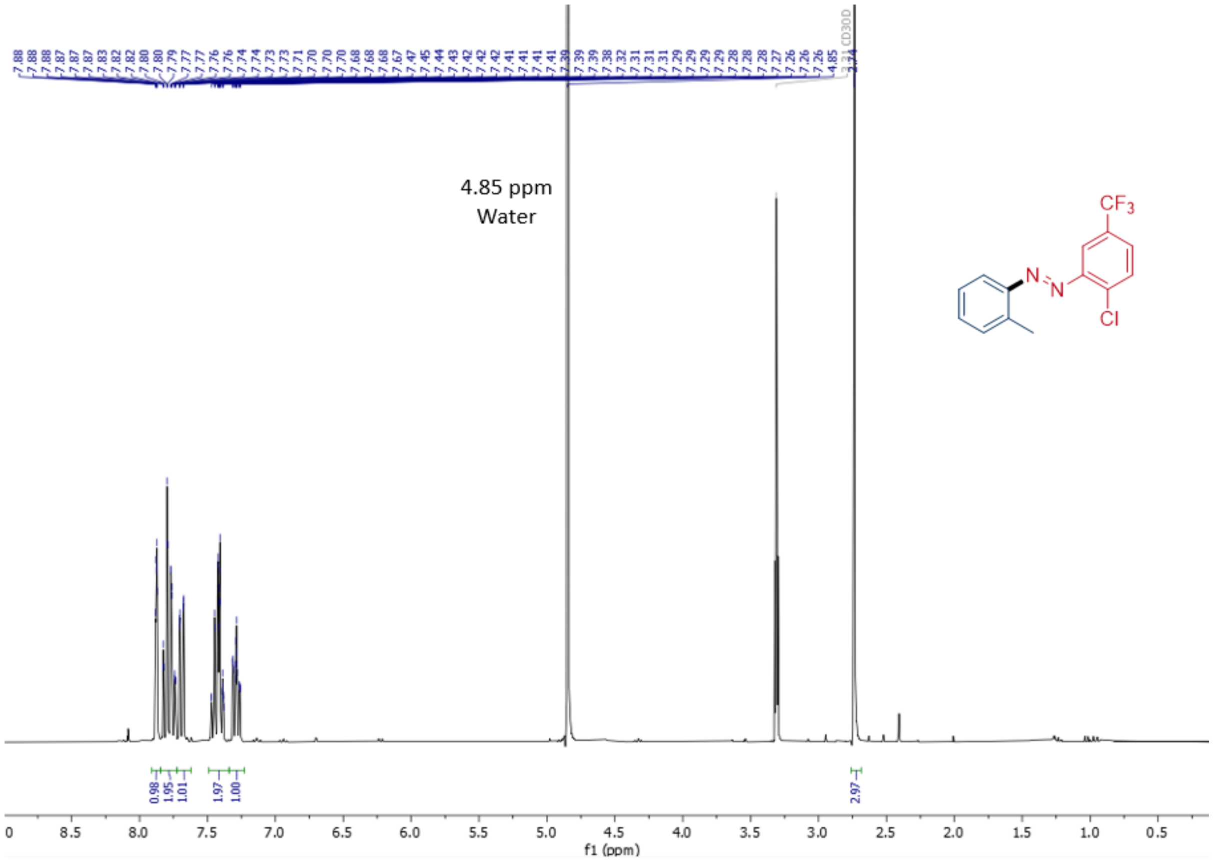


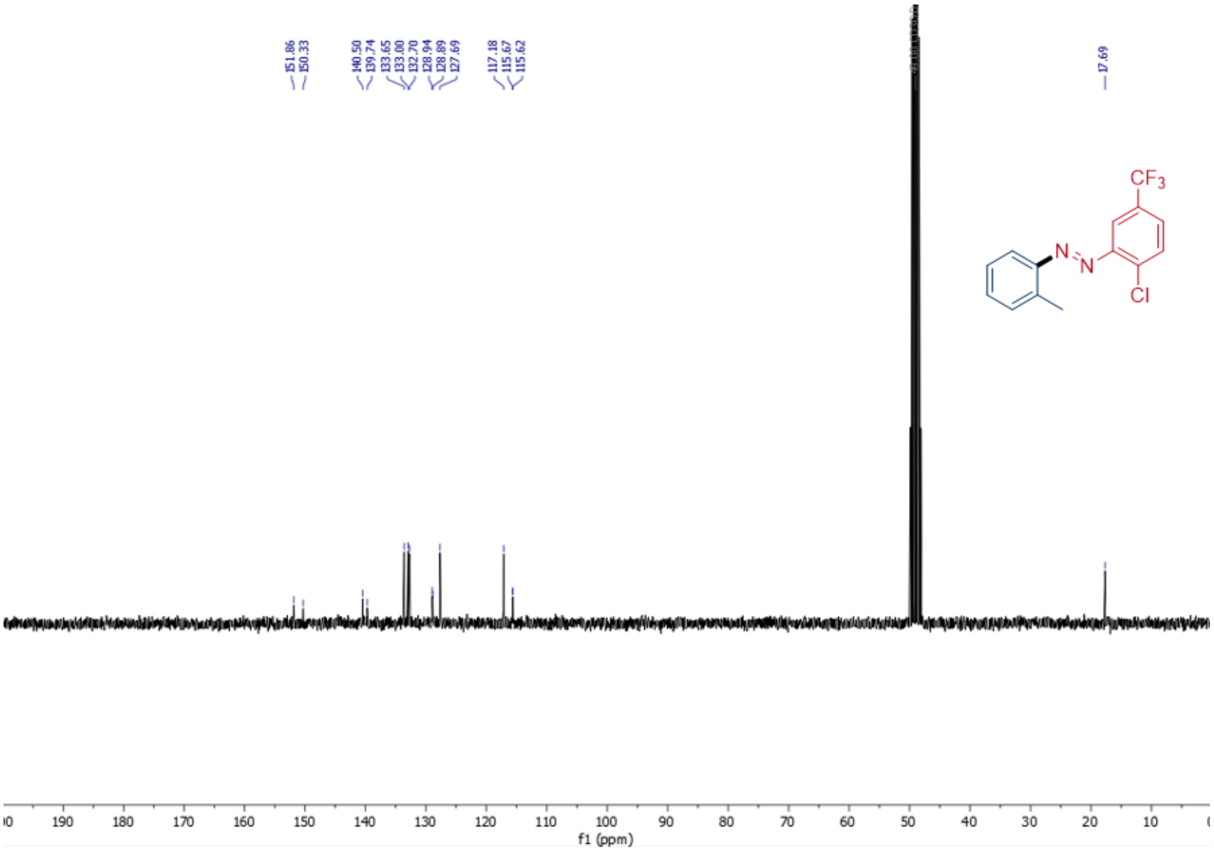

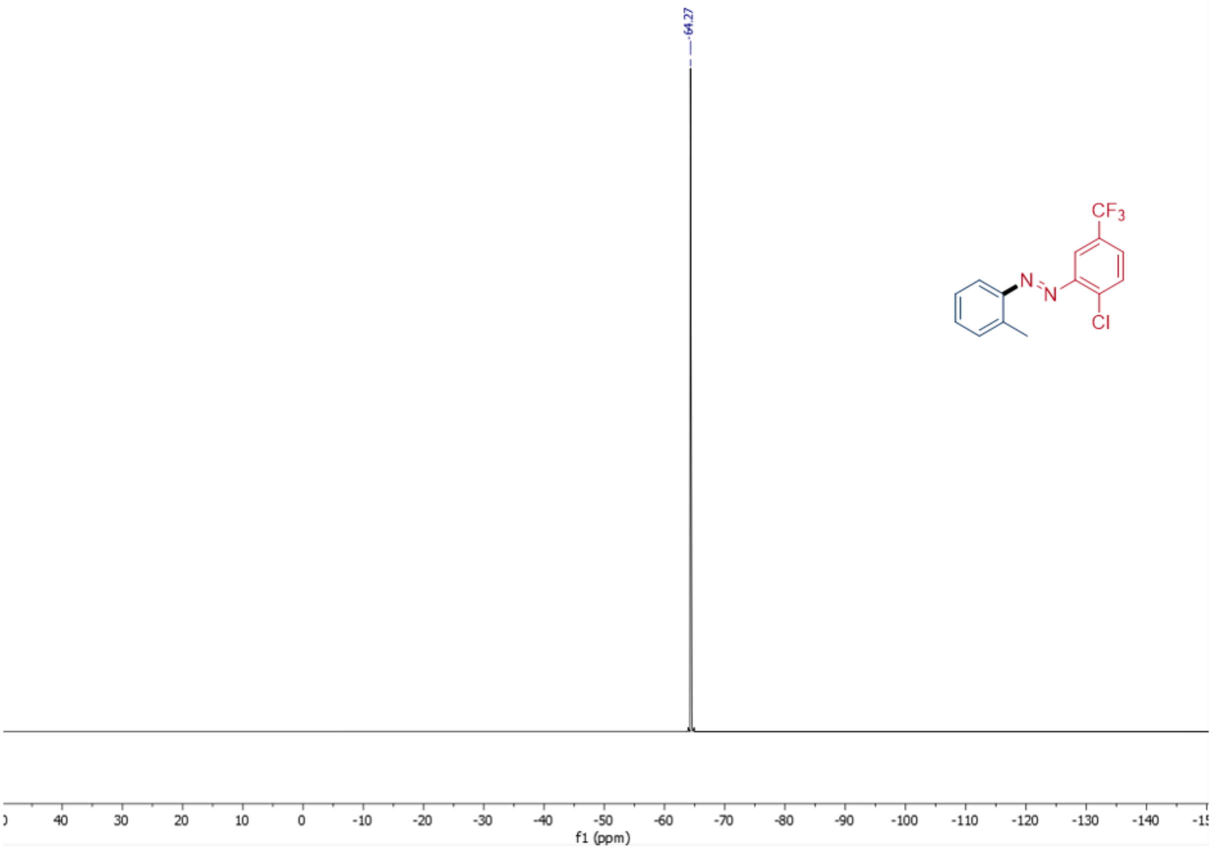
-64.27
CF3
N
N
Cl
40
30
20
10
0
-10
-20
-30
-40
-50
-60
-70
-80
-90
-100
-110
-120
-130
-140
f1 (ppm)

**$^{1}H$, $^{13}C$, $^{19}F$ NMR of 5a:**

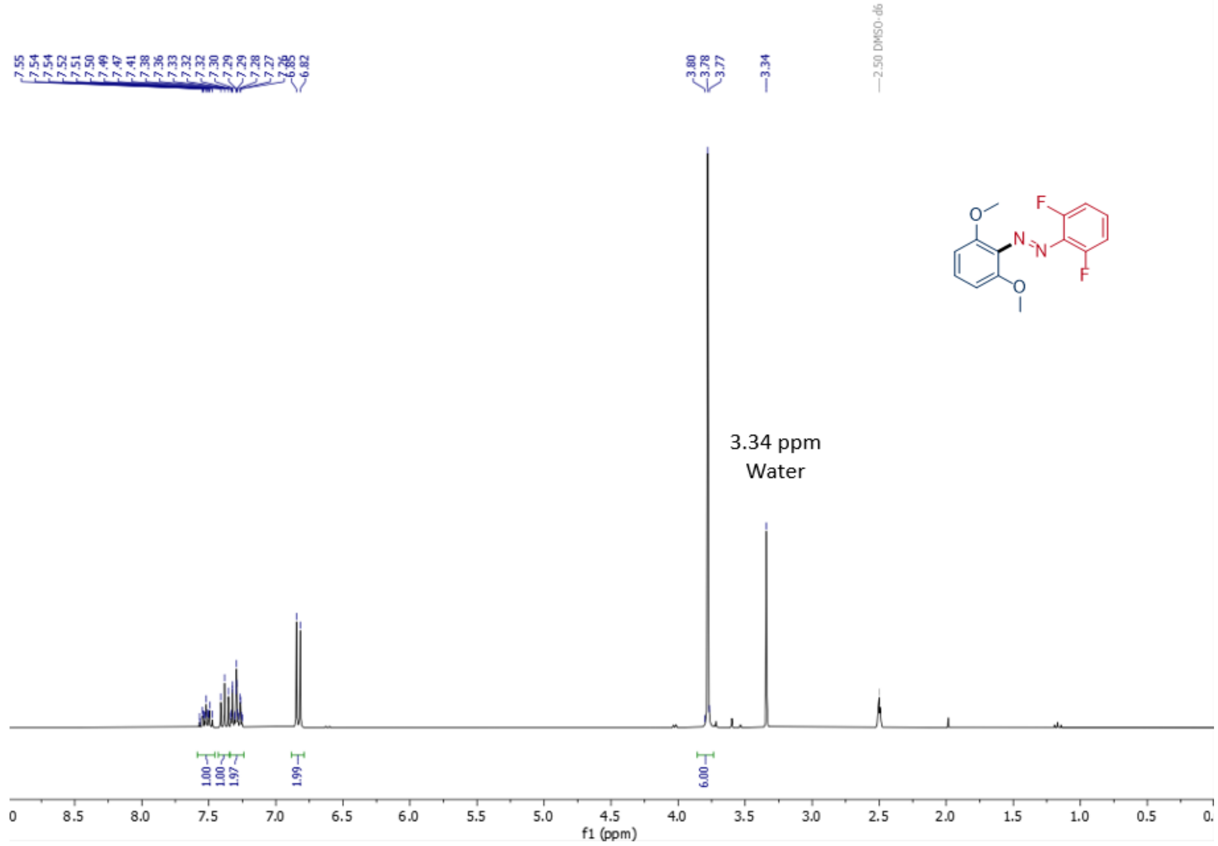


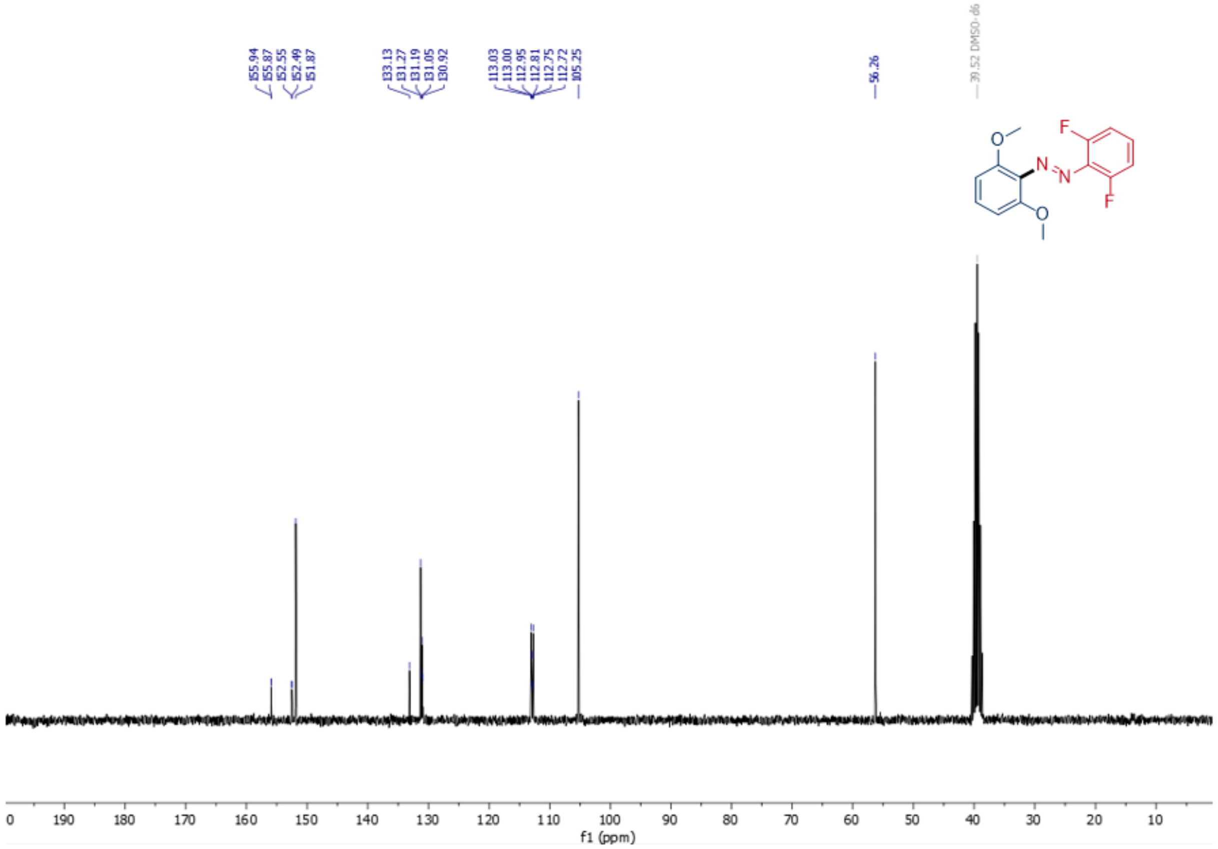

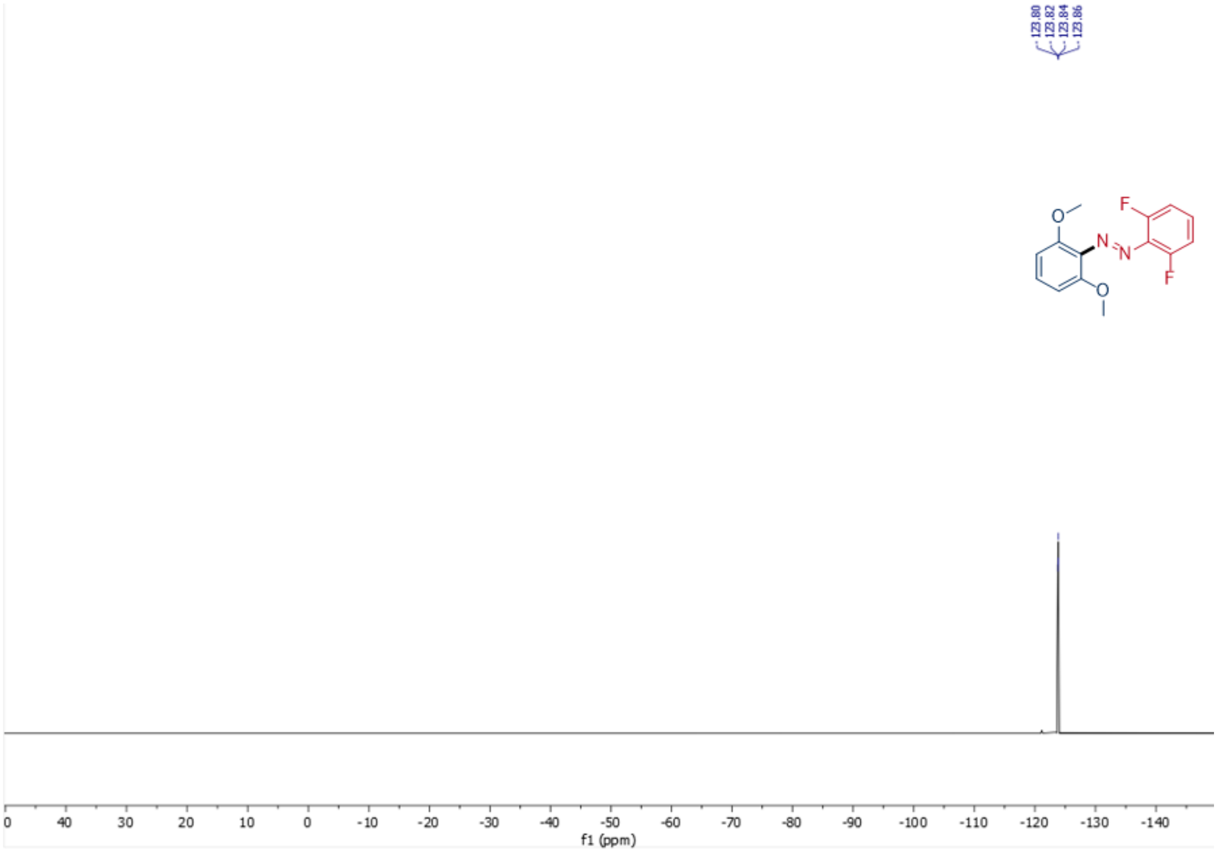
-123.80
-123.82
-123.84
-123.86
F
F
O
O
N
N
f1 (ppm)

**$^1H$, $^{13}C$, $^{19}F$ NMR of 5b:**

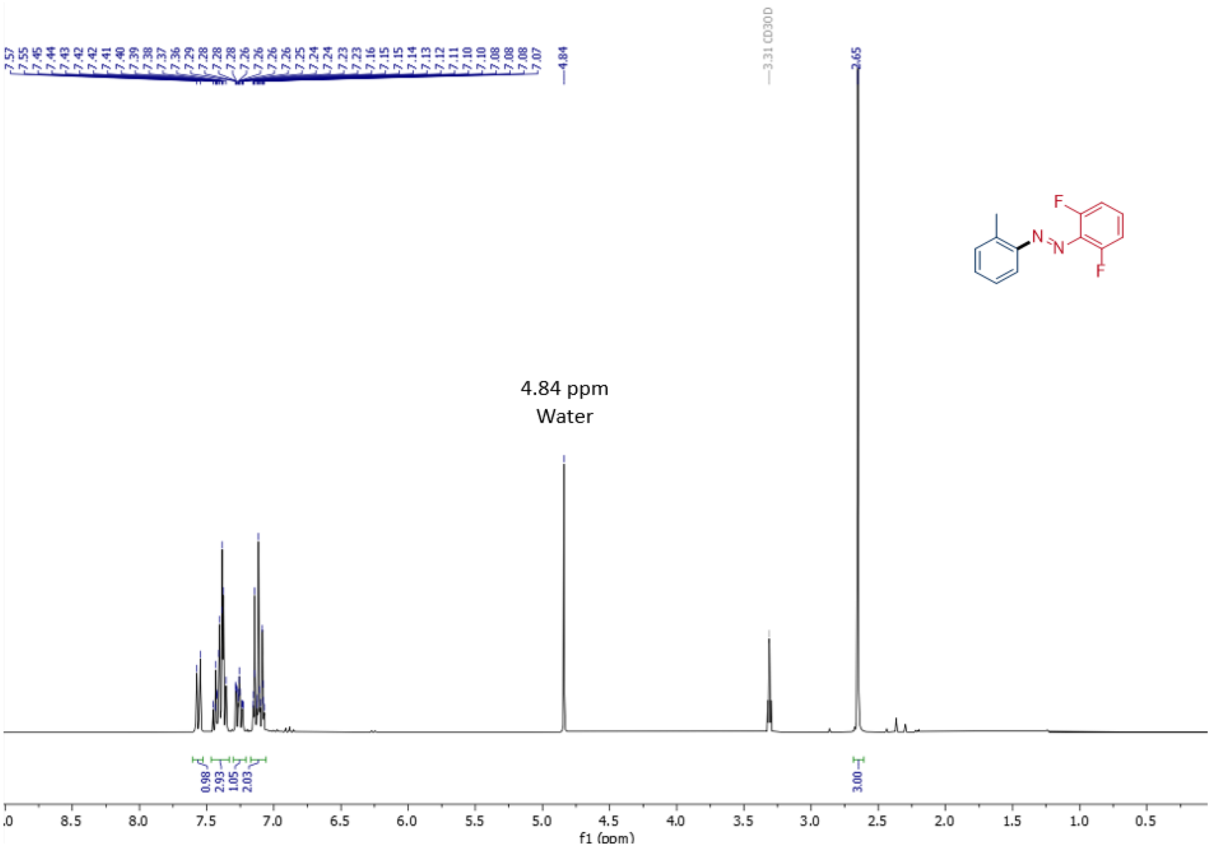


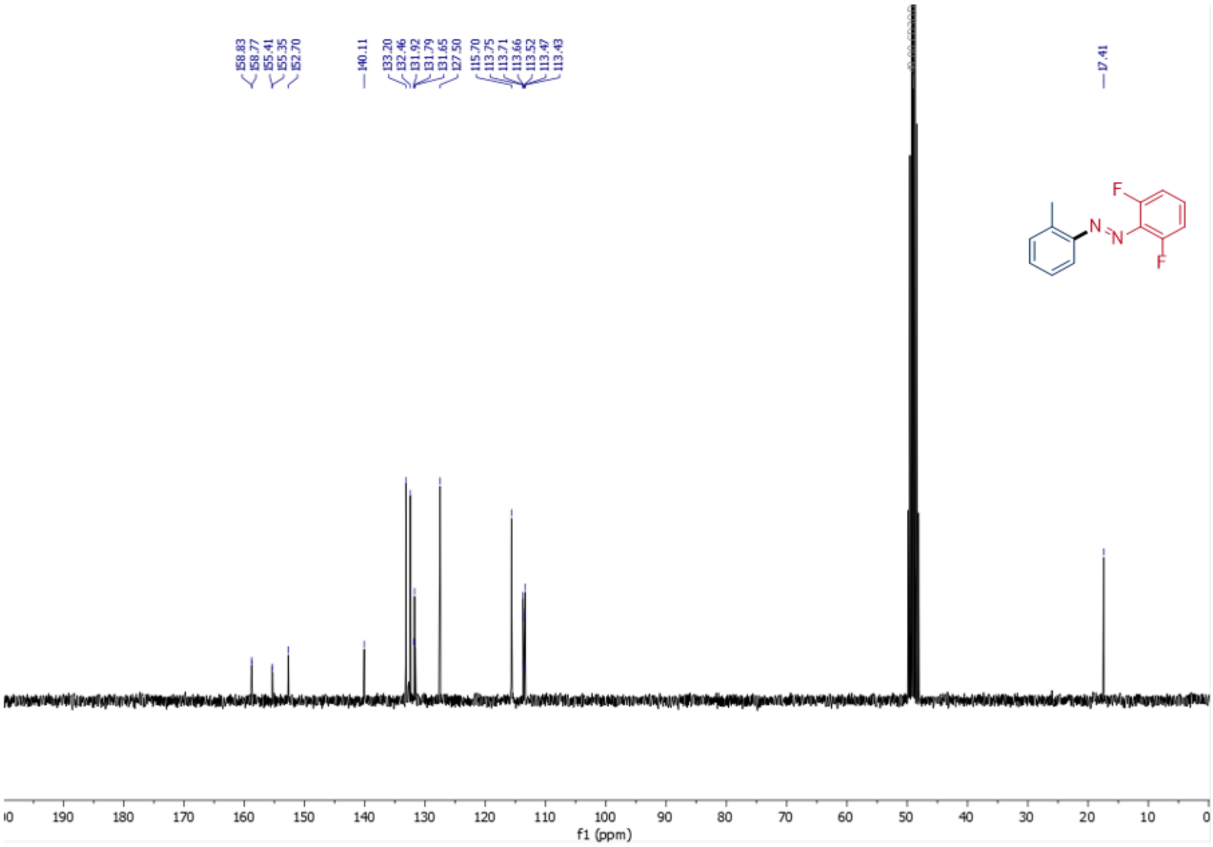

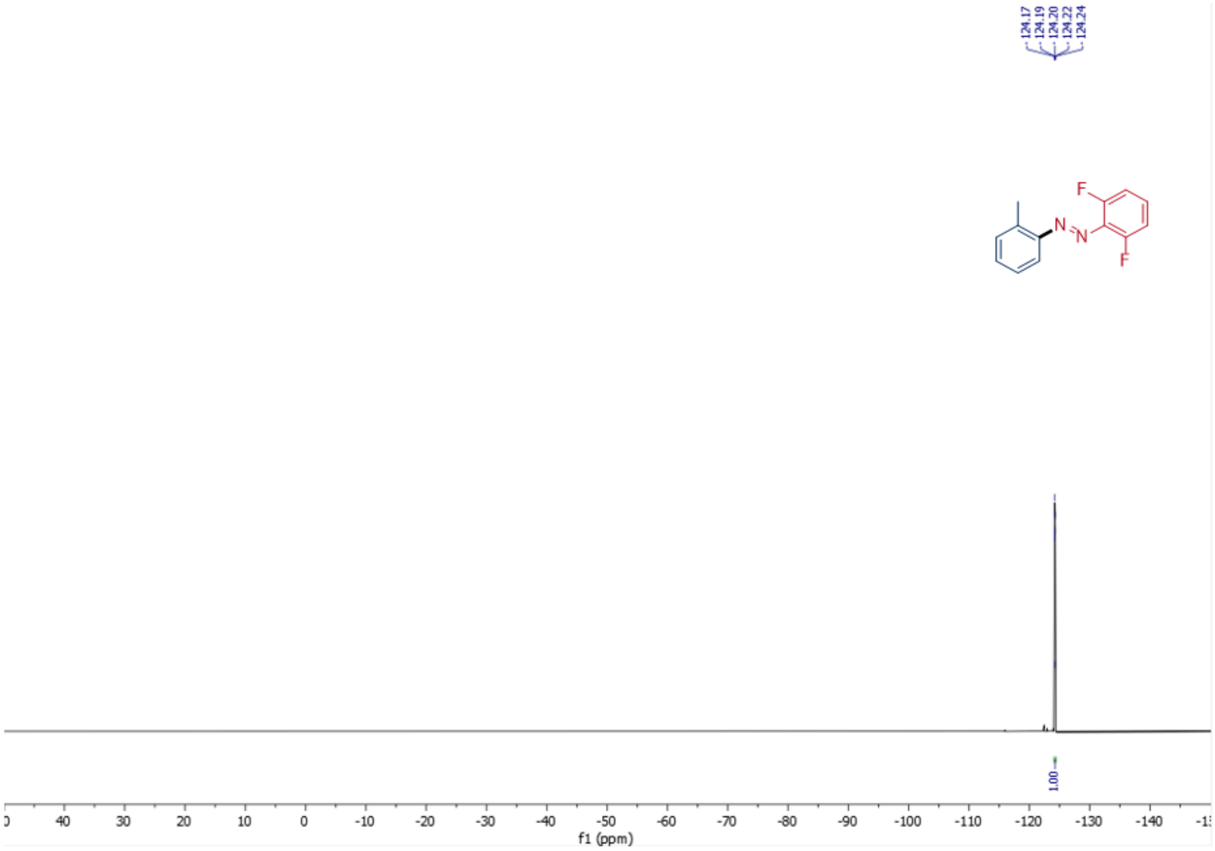

124.17
124.19
124.20
124.22
124.24
F
N
N
F
1.00
40
30
20
10
0
-10
-20
-30
-40
-50
-60
-70
-80
-90
-100
-110
-120
-130
-140
f1 (ppm)

**$^{1}$H, $^{13}$C NMR of 5c:**

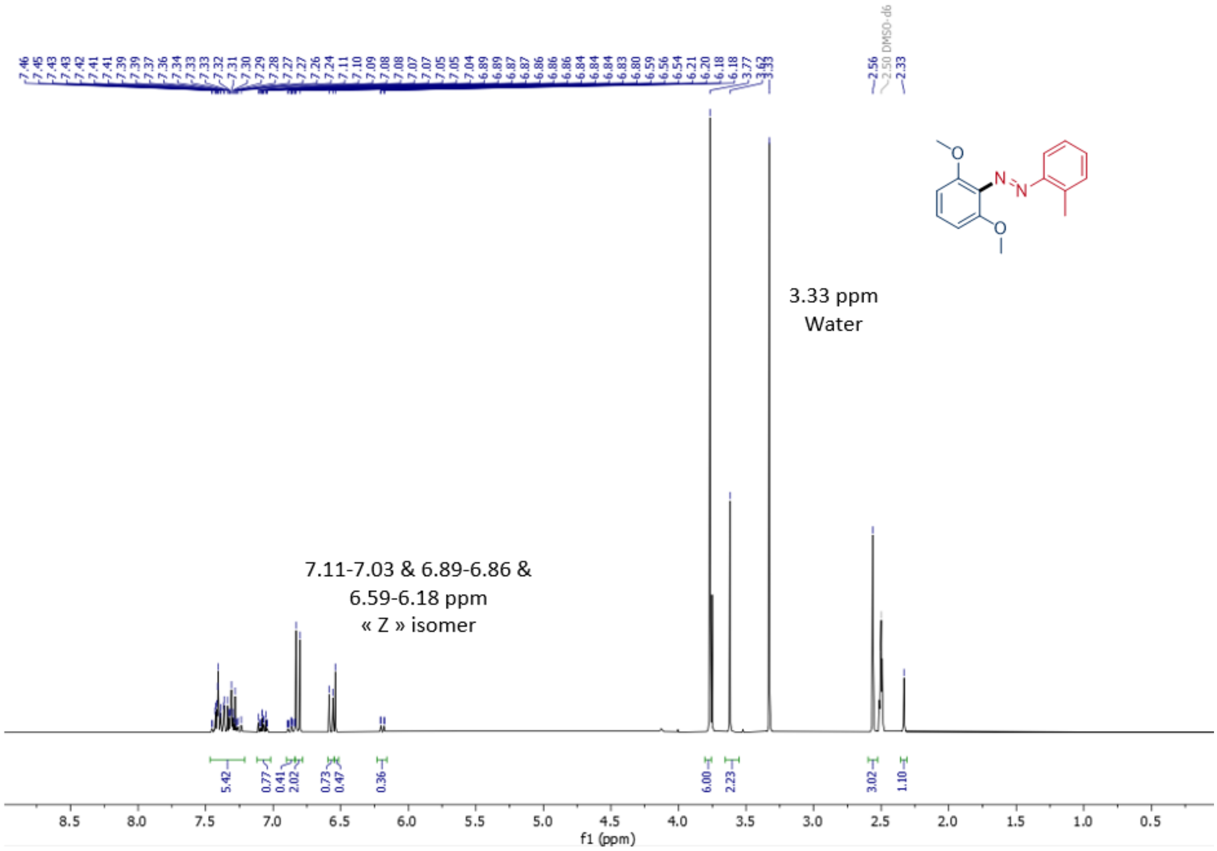


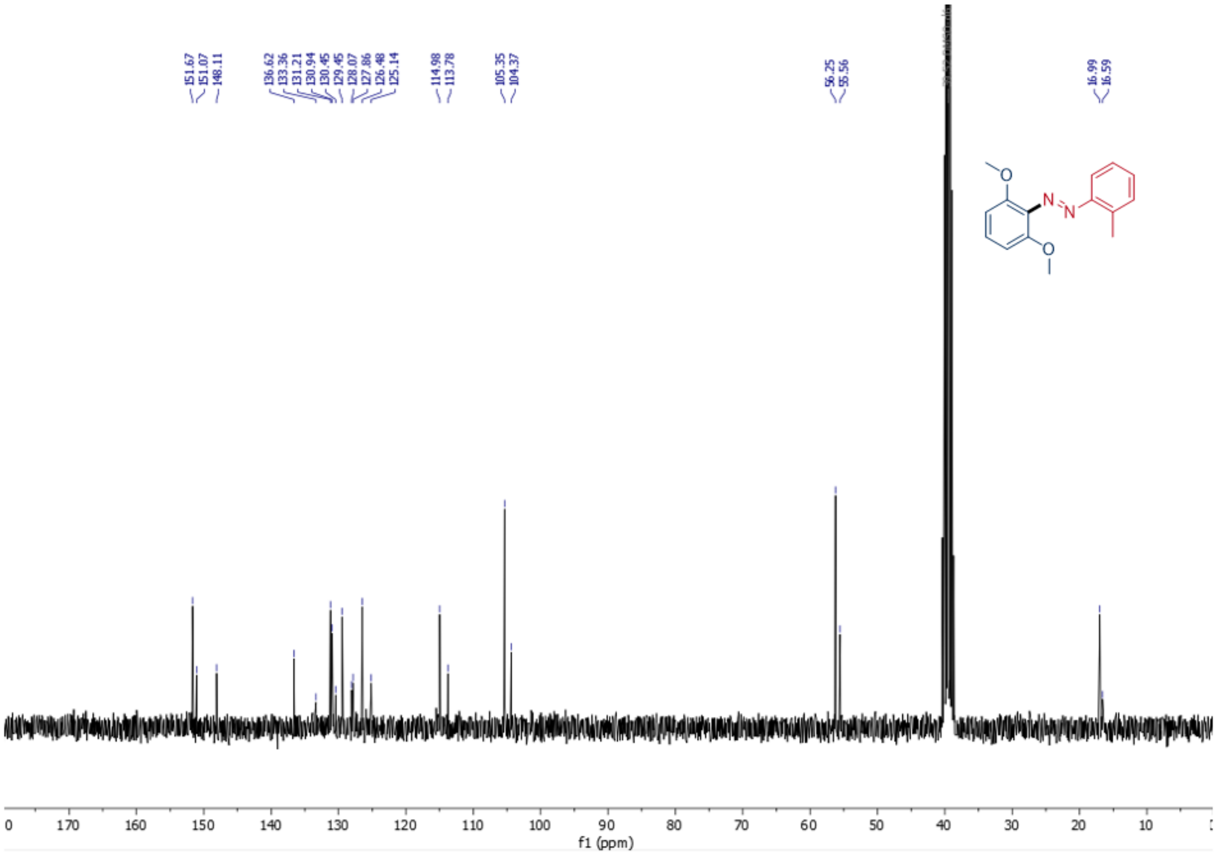

**$^1H$, $^{13}C$, $^{19}F$ NMR of 5d:**

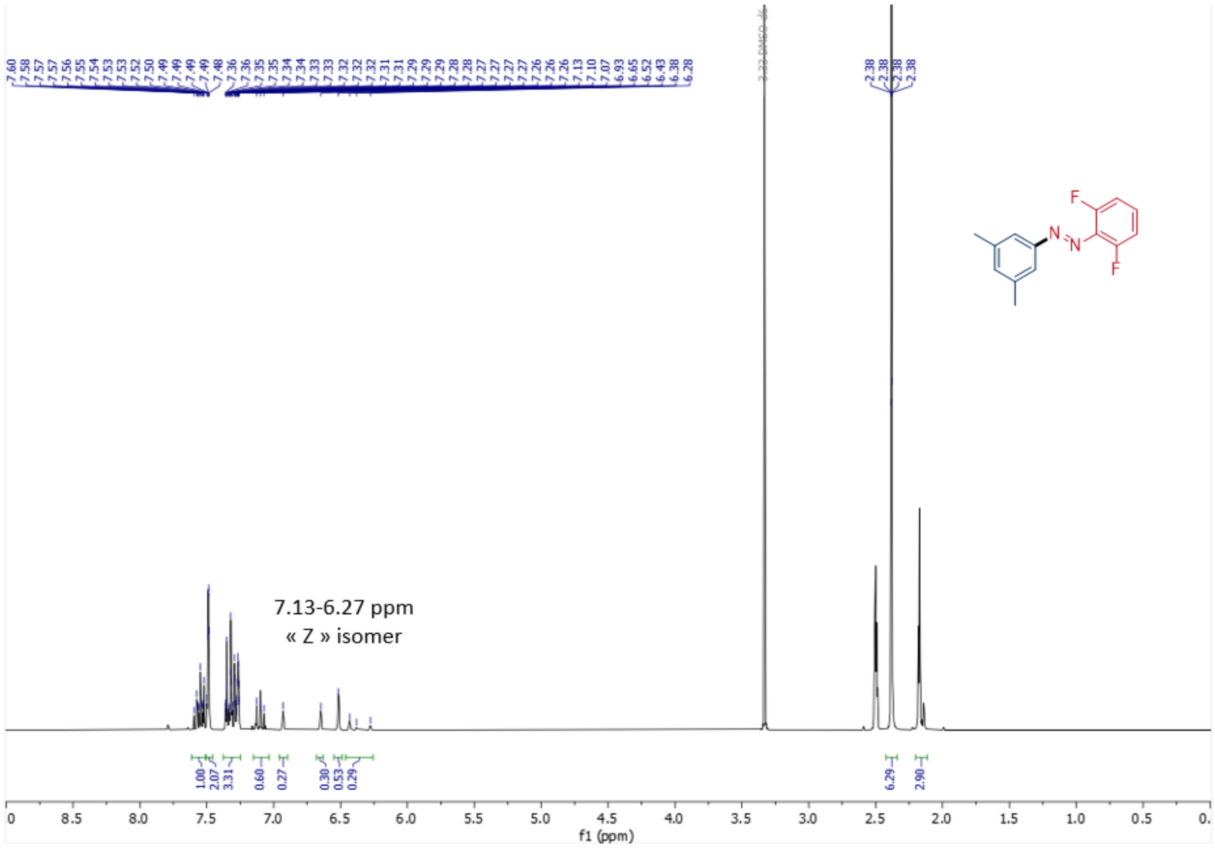


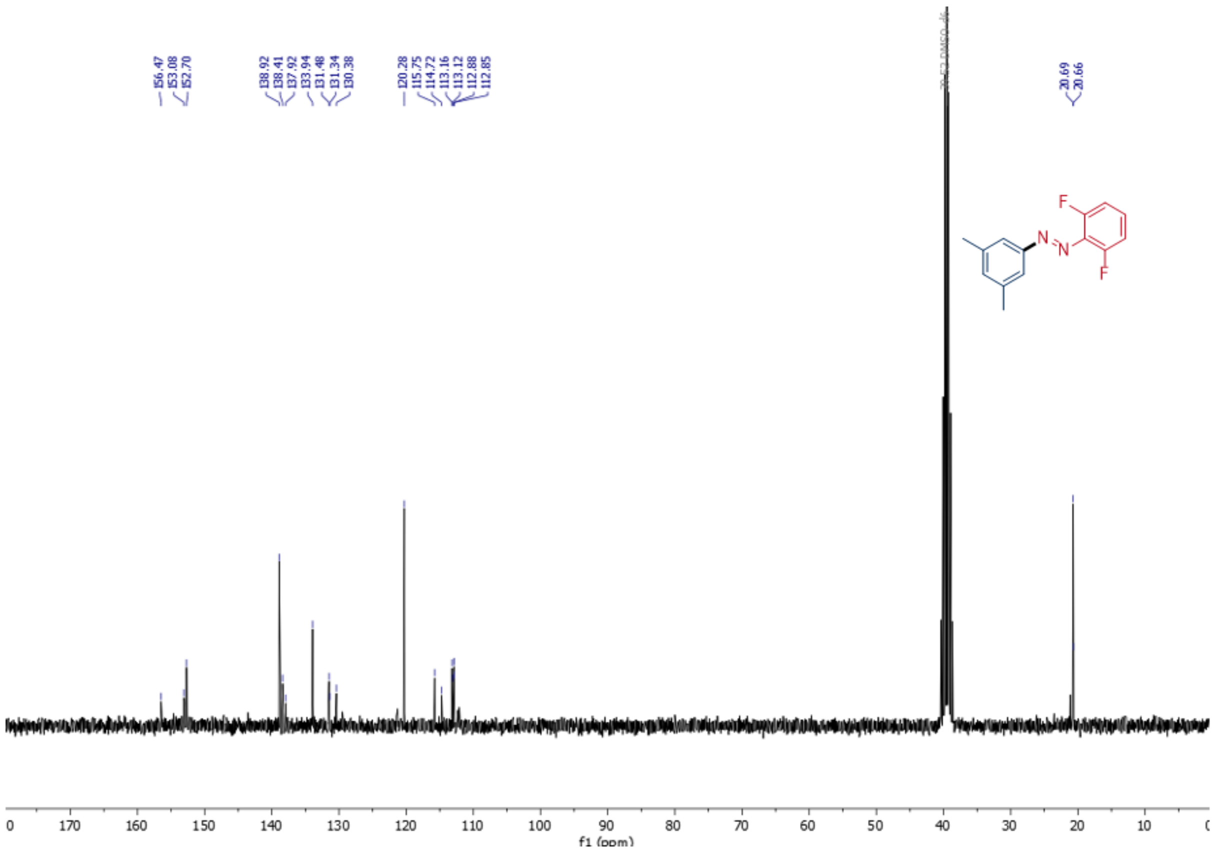

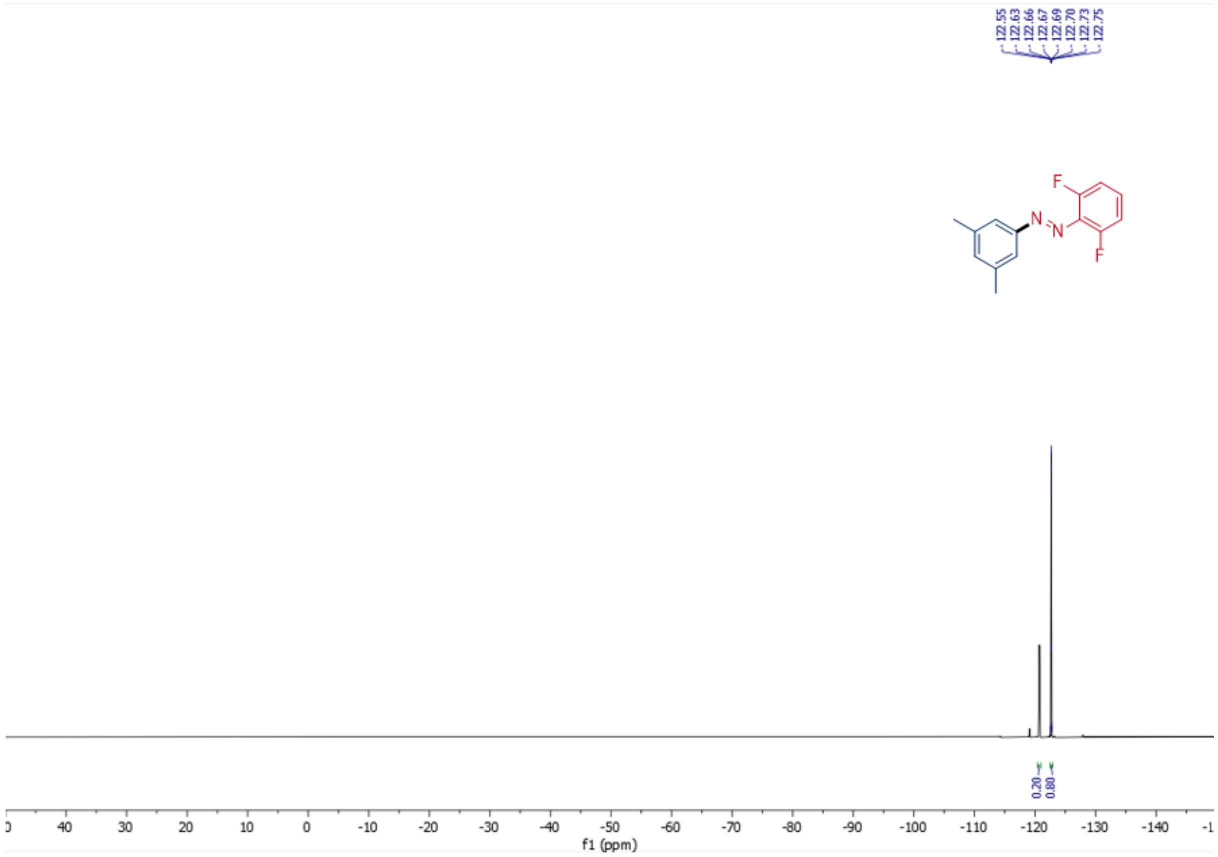
122.55
122.63
122.66
122.67
122.69
122.70
122.73
122.75
F
N
N
F
0.20
0.80
40
30
20
10
0
-10
-20
-30
-40
-50
-60
-70
-80
-90
-100
-110
-120
-130
-140
f1 (ppm)
